# Ninety-year trends reveal sharpest insect declines in the mid-twentieth century

**DOI:** 10.1038/s41559-026-03074-6

**Published:** 2026-06-02

**Authors:** Felix Neff, Kurt Bollmann, Yannick Chittaro, Martin M. Gossner, Felix Herzog, Fränzi Korner-Nievergelt, Glenn Litsios, Carlos Martínez-Núñez, Marco Moretti, Emmanuel Rey, Andreas Sanchez, Eva Knop

**Affiliations:** 1 Agricultural Landscapes and Biodiversity, Agroscope, Zurich, Switzerland; 2 Biodiversity and Conservation Biology, Swiss Federal Research Institute WSL, Birmensdorf, Switzerland; 3 info fauna, Neuchâtel, Switzerland; 4 Forest Health and Biotic Interactions, Swiss Federal Research Institute WSL, Birmensdorf, Switzerland; 5 Department of Environmental System Science, Institute of Terrestrial Ecosystems, ETH Zurich, Zurich, Switzerland; 6 Swiss Ornithological Institute, Sempach, Switzerland; 7 Department of Environmental Sciences, Landscape Ecology and Ecosystems Conservation and Research Station Petite Camargue Alsacienne, University of Basel, Basel, Switzerland; 8 Department of Ecology and Evolution, Estación Biológica de Doñana EBD (CSIC), Seville, Spain; 9 Department of Evolutionary Biology and Environmental Studies, University of Zurich, Zurich, Switzerland

**Keywords:** Biodiversity, Climate-change ecology, Community ecology

## Abstract

Most studies on insect trends have focused on the past few decades, but major changes could have occurred earlier. If so, shifted baselines can result in misleading conclusions or poor conservation decisions. Here we analyse nine decades of insect trends in Switzerland and relate them to changes in land use and climate. By reconstructing continuous trends in species richness based on 1.2 million records of 595 saproxylic beetle and 216 butterfly species, we find that both groups declined from the 1930s to the 1960s. While saproxylic beetle richness stabilized and subsequently recovered, butterfly richness continued to decline until the 1980s and has not recovered. The strong mid-century decreases were linked to increases in agricultural mechanization, while the subsequent increases were linked to climate warming. Over the entire 90-year period, declines primarily affected specialist and cold-adapted species, while warm-adapted species have increased since the 1980s. Recent gains in saproxylic beetle richness might also reflect increased deadwood availability from windthrow and ‘biodiversity-friendly’ forest management. Our findings suggest that reducing adverse land use is key to maintaining and promoting insect diversity, and that shifted baselines and climate-change effects should be considered when setting restoration goals and priorities.

## Abstract (web summary)

Assessing the magnitude and drivers of recent biodiversity losses requires informative, long-term baseline data. Here the authors reconstruct the temporal trajectories of butterflies and saproxylic beetles in Switzerland from 1930 to 2021, shedding light on the role of climate and land-use change.

## Main

Alarming findings on a massive insect decline^1^ have sparked research into temporal trends in insect diversity^2,3^. However, most studies have covered a maximum of a few decades^4,5^, despite increasing anthropogenic pressure on insect diversity throughout the entire twentieth century^5^. It is therefore hypothesized that these comparatively short time series fail to capture the most detrimental changes resulting from global change because they miss an important time span^6^. The few studies that cover more than a few decades are often limited to single time points (for example, data from the twentieth century compared with data from the twenty-first century^7^), to several discrete time windows over the twentieth century^8^, or to a small number of study sites or single taxonomic groups when analysing continuous data^9–12^. Most of these studies found support for long-term declines in insect diversity. A continuous long-term time series of insect diversity, covering a large spatial scale and different insect groups, is still lacking, but will help to better understand community changes over more than 40 years—including the time of large-scale agricultural intensification in the second half of the twentieth century^13^. Furthermore, continuous long-term time series of insect diversity can provide a quantification of the biases due to the shifting baseline phenomenon in studies that start later^5,14^, which will be key for prioritizing insect conservation action.

**Fig. 1:**
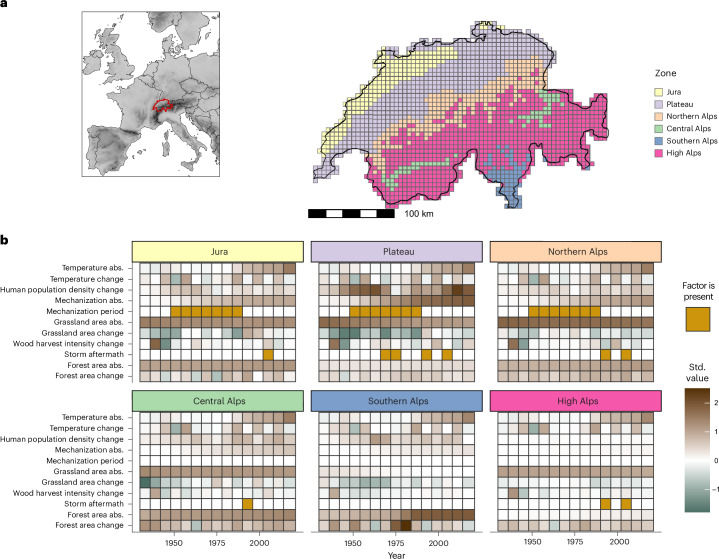
Study region and changes in environmental conditions since 1930. **a**, The study country, Switzerland, is located along the Alps in Central Europe and was divided into six biozones based on biogeographic regions and elevation (indicated by different colour shadings). Species records were analysed at the resolution of 5 km × 5 km squares (overlaid grid). **b**, Variables indicating environmental conditions and changes therein for the period 1930 to 2021, shown separately for each of the six biozones. Values are shown for intervals of 8 years (1930–1937, 1936–1943 and so on), in which variables of absolute (abs.) values (for example, temperature abs.) are centred intercepts and variables of change (for example, temperature change) are slope coefficients from linear regressions. Two variables (mechanization period, storm aftermath) are two-level factors, in which the presence of the factor is indicated in orange. For comparability, continuous variables were standardized (std.) to standard deviation of 0.5 and a minimum of 0 (the latter only for variables with exclusively positive values). See Supplementary Figs. 3–8 for details.

Here we reconstructed the temporal trajectories of insect species distributions in Switzerland between 1930 and 2021, based on 1.2 million species records and occupancy-detection models^15^. We focused on two functionally distinct groups: saproxylic beetles and butterflies. Saproxylic beetles depend on deadwood and comprise various trophic levels, including decomposers, fungivores and predators^16^. They are predominantly found in forests and other wooded areas, where they play a vital role in decomposing deadwood^17^, and many species, particularly longhorn and jewel beetles, feed on pollen as adults. Butterflies are important pollinators as adults and herbivores as caterpillars^18,19^. They are distributed in various habitats, but most species in the study region are found in open, agriculturally managed habitats^20^. We estimated the distribution of 595 saproxylic beetle species (out of approximately 1,000 species in Switzerland) and 216 butterfly species (out of approximately 230 species) in terms of the mean occupancy of 5 km × 5 km squares (that is, mean number of occupied squares) across the entire study period (1930–2021, aggregated in 2-year intervals) and for 6 biozones (Fig. 1a). These biozones comprise a wide range of environmental conditions and trajectories therein (Fig. 1b) and were derived from biogeographic regions^21^ and elevation (the high Alps biozone includes all squares with a mean elevation above 1,400 m above sea level (a.s.l.)). We compared the estimated temporal changes of species’ mean occupancies to red list status and a temporally restricted standardized butterfly monitoring scheme and found strong congruence (Supplementary Figs. 1 and 2). Based on mean occupancy per species, we estimated the average species richness per square for both insect groups in each biozone and 2-year interval (sum of mean occupancies). Furthermore, to obtain an overall trend, we aggregated the data for Switzerland as a whole.

## Results and discussion

### Temporal changes of species richness

**Fig. 2:**
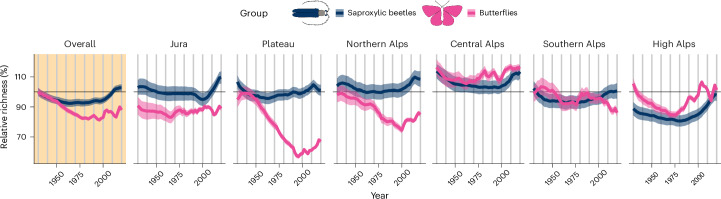
Change in saproxylic beetle and butterfly species richness since 1930 distinguished by biozones. Trajectories of average species richness per 5 km × 5 km square, shown relative to the value for the whole of Switzerland in 1930 (first panel on the left), for saproxylic beetles and butterflies. The first panel (yellow shading) shows values for all of Switzerland; the other panels show values for individual biozones (Fig. 1a). Lines represent means; shaded areas indicate 95% CIs. Vertical lines mark decade boundaries.

From 1930 to the 1960s, the relative average species richness in Switzerland declined continuously for both saproxylic beetles and butterflies (Fig. 2). The strongest declines were observed in the 1930s for saproxylic beetles, with an estimated decrease in relative richness of −3.1% (95% credible interval (CI): −4.9% to −1.2%) per decade, and in the 1950s for butterflies, with a decrease of −4.5% (95% CI: −6.4% to −2.6%) per decade. For saproxylic beetles, these declines were followed by increases, most notably in the 2000s with an estimated increase of 3.9% (95% CI: 2.2% to 5.5%) per decade (Fig. 2). For butterflies, however, the decline continued and began to level off only in the 1980s (Fig. 2). Since then, butterfly richness has fluctuated, maintaining roughly the level observed in the 1980s. Consequently, the richness of saproxylic beetles was estimated to be similar at the end of the study period (2020–2021) compared with the beginning (1930–1931), whereas butterfly richness decreased by −12.0% (95% CI: −14.3% to −9.5%). The decline in butterfly richness is smaller than the declines reported for insects over shorter time periods^1,3,22^. However, this may be attributed to differences in the spatial scale of analyses. Our estimates represent regional richness (5 km × 5 km squares), whereas previous studies mostly reported declines at the local plot scale.

The continuous decrease in saproxylic beetles until the 1960s and in butterfly richness until the 1980s has been hypothesized before^6^, but, to our knowledge, has not previously been analysed with such fine temporal resolution at a national scale. Furthermore, our results provide compelling evidence that shifted baselines are an important concern in short-term studies of temporal trends in insects^4^. For example, if we had considered only the past four decades of the present dataset, we would have estimated a Switzerland-wide increase in richness of 10.8% (95% CI: 8.1% to 13.5%) for saproxylic beetles and 6.5% (95% CI: 4.8% to 8.4%) for butterflies. These figures clearly differ from the 90-year estimates of +2.7% (95% CI: −0.2% to 5.7%) and −12.0% (95% CI: −14.3% to −9.5%), respectively. Thus, the limitation of shifted baselines should be acknowledged more when interpreting short time series^5,14^.

Trajectories of richness clearly differed between the biozones (Fig. 2). For saproxylic beetles, we observed similar declines in the early decades in all biozones, but recovery was less evident in the plateau and southern Alps biozones. For butterflies, there was a particularly strong and continuous decline in richness in the plateau and northern Alps biozones (Fig. 2). Over the full study period, butterfly species richness decreased by −29.2% (95% CI: −33.0% to −25.2%) in the plateau, where the majority of the Swiss population, infrastructure and crop production are located, and by −13.3% (95% CI: −17.8% to −8.5%) in the northern Alps. In the Jura and central Alps biozones, we observed the smallest changes in butterfly richness. While butterfly richness started to recover to some extent in all regions from the end of the twentieth century onwards, this was not the case in the southern Alps.

### Drivers of insect richness changes

Many studies have highlighted the link between changes in insect communities and climate and land-use changes^2,3,23,24^. Over the 90-year study period, climate warming was evident in all biozones from the 1980s onwards^25^ (Fig. 1b and Supplementary Fig. 3). Agricultural intensification was strongest in the lowland plateau biozone and the adjacent mountainous Jura and northern Alps biozones, becoming most pronounced from the 1950s to the 1980s (Fig. 1b and Supplementary Fig. 4). Wood harvest intensity, an indicator of forest management intensity and deadwood removal, was particularly high during World War II and increased slightly in the plateau, Jura and northern Alps biozones over the course of the twentieth century (Fig. 1b and Supplementary Fig. 5). Concurrently, the vital role of deadwood in forest ecosystems was increasingly recognized in forest management, leading to an increase in deadwood in forests from the 1990s onwards^26^, which was also promoted by several extreme storm events leading to windfall (Supplementary Fig. 5).

The differences in environmental conditions and changes therein between the biozones (Fig. 1b) can explain the differences in species richness trajectories between zones (Fig. 2). For example, the strong declines in butterfly species richness in the plateau and northern Alps biozones coincide with the zones that underwent the most substantial agricultural mechanization (Fig. 1b and Supplementary Fig. 4). We directly tested the influence of multiple drivers, particularly land use and climate change, on trends in richness of saproxylic beetles and butterflies. To this end, we divided the study period into 8-year intervals and calculated the relative species richness trend for each biozone in each interval. We then related these trends to the state and change of a set of environmental variables for the same intervals and biozones (Fig. 1b) using regression models.

**Fig. 3:**
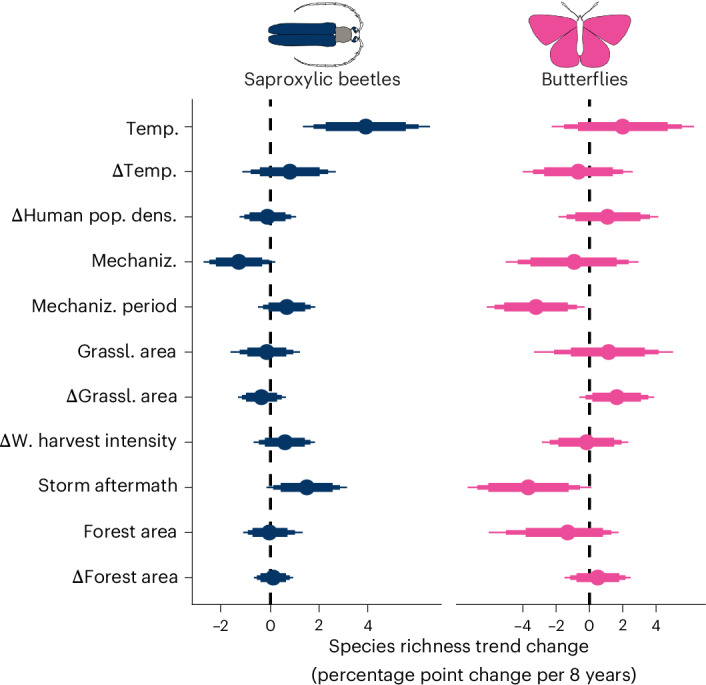
Regression model results linking relative richness trends to (changes in) environmental conditions. Posterior distributions of slope estimates from regression models relating the trend of relative richness in a biozone and 8-year interval to a set of environmental variables for the same zone and interval. Modelled slope estimates were transformed to represent the change in the species richness trend in an 8-year interval. For example, an effect size of 2 means that if the respective variable is increased by 0.5 s.d., the richness change in an 8-year interval (relative to the overall value in 1930) is 2 percentage points higher. Separate models were fitted for saproxylic beetles and butterflies. Continuous predictor variables were scaled to s.d. 0.5 before the analyses. Points show means; segments of varying thickness show 80%, 90% and 95% CIs. *n* = 90 observations (biozone–interval combinations) per model. Temp., temperature absolute; ΔTemp., temperature change; ΔHuman pop. dens., human population density change; Mechaniz., mechanization absolute; Mechaniz. period, mechanization period (factor); Grassl. area, grassland area absolute; ΔGrassl. area, grassland area change; ΔW. harvest intensity, wood harvest intensity change.

Absolute temperature, expressed as the average temperature anomaly over an 8-year interval, was positively related to species richness trends, particularly for saproxylic beetles (Fig. 3), indicating more positive richness trends in warmer time intervals. For saproxylic beetles, an increase in absolute temperature of 0.5 s.d. (0.34 °C) resulted in a 2.0 percentage point (95% CI: 0.67 to 3.3) increase in the species richness change per 8-year interval (richness relative to the average richness in Switzerland in 1930). The increase in the richness of saproxylic beetles in warmer climates has previously been observed and may reflect the higher survival and dispersal of saproxylic beetle species at higher temperatures^27^. When the model predictions are scaled to an absolute temperature increase of 2 °C, which occurred in Switzerland over the past 100 years^25^ (Supplementary Fig. 3), the estimated increase in richness trends of saproxylic beetles is 12.0 percentage points (95% CI: 3.9 to 19.0). Therefore, climate warming since the 1980s (Supplementary Fig. 3) has potentially contributed to the (partial) recovery of species richness in saproxylic beetles.

We quantified the degree of agricultural mechanization with the number of tractors reported in each biozone relative to its absolute area. We identified a clear period of mechanization between the 1950s and the 1980s in three biozones (Fig. 1b and Supplementary Fig. 4). Species richness trends were clearly related to agricultural mechanization (Fig. 3). An increase in absolute mechanization of 0.5 s.d. (0.92 tractors per km^2^) was associated with a −0.65 percentage point (95% CI: −1.4 to 0.096) reduction in richness trends per 8-year interval for saproxylic beetles. This negative relationship potentially reflects the close links between agriculture and silviculture in Switzerland, where many farmers are also forest owners^28^. Thus, agricultural mechanization may have largely coincided with silvicultural mechanization. For much of the twentieth century, increasing mechanization resulted in the intensification of forest management, which typically leads to the simplification of forest structures and a reduction in the amount of available deadwood^16,29^. These relationships would explain the generally negative relationship between mechanization and temporal trends in saproxylic beetle richness. For butterflies, periods of mechanization—the time intervals during the transition from low to high absolute mechanization between the 1950s and 1980s in three biozones (Fig. 1b and Supplementary Fig. 4)—were associated with a −3.2 percentage point reduction (95% CI: −6.2 to −0.30) in richness trends (Fig. 3). This emphasizes that agricultural intensification in the second half of the twentieth century was a major cause of the decline of butterflies, a group of insects that is strongly associated with open, agriculturally managed habitats such as semi-natural grasslands^30^. The strong increase in mechanization was accompanied by several changes in agricultural practices, such as the use of more efficient machinery (for example, modern rotary mowers), the replacement of permanent grassland with rotational grassland, the increased use of fertilizers and pesticides, and the structural homogenization of landscapes (for example, the removal of structural elements and larger field sizes)^13,31^. All these changes contributed to the negative effects on butterflies^32,33^. Therefore, over the past 90 years, mechanization and agricultural intensification have been detrimental to both butterfly and saproxylic beetle communities.

Following the growing awareness of environmental issues in the 1970s, biodiversity-friendly management practices were increasingly promoted in Switzerland towards the end of the twentieth century. In forests, near-to-nature management became widely adopted, which resulted, for example, in an increase in deadwood—a change that should have promoted many saproxylic beetle species^29^. Indeed, the change in forest management undoubtedly contributed to the recovery of saproxylic beetle richness in the second half of the study period (Fig. 2). In open lands, the introduction of agri-environmental measures in the 1990s and the subsequent increase in the amount and connectivity of suitable habitats in agricultural areas probably contributed to stabilizing butterfly richness trends^34^ (Fig. 2). While these management changes certainly contributed to trend stabilizations and reversals, quantifying these effects, particularly with regard to different measures and species groups, is beyond the scope of our study.

Finally, we found evidence that trends in saproxylic beetle species richness were associated with large storm events (for example, storm Vivian in 1990 and storm Lothar in 1999; Fig. 3). The trend in saproxylic beetle richness per 8-year interval was 1.5 percentage points (95% CI: −0.16 to 3.1) higher in intervals following large storm events. This can be explained by increased amounts of deadwood and better light conditions following storms, which benefit saproxylic insects^35^. Given the expected increase in storm frequency due to climate change^25^, some insect groups, such as saproxylic beetles, may benefit from increased food and habitat availability.

### Trait-dependent richness changes

**Fig. 4:**
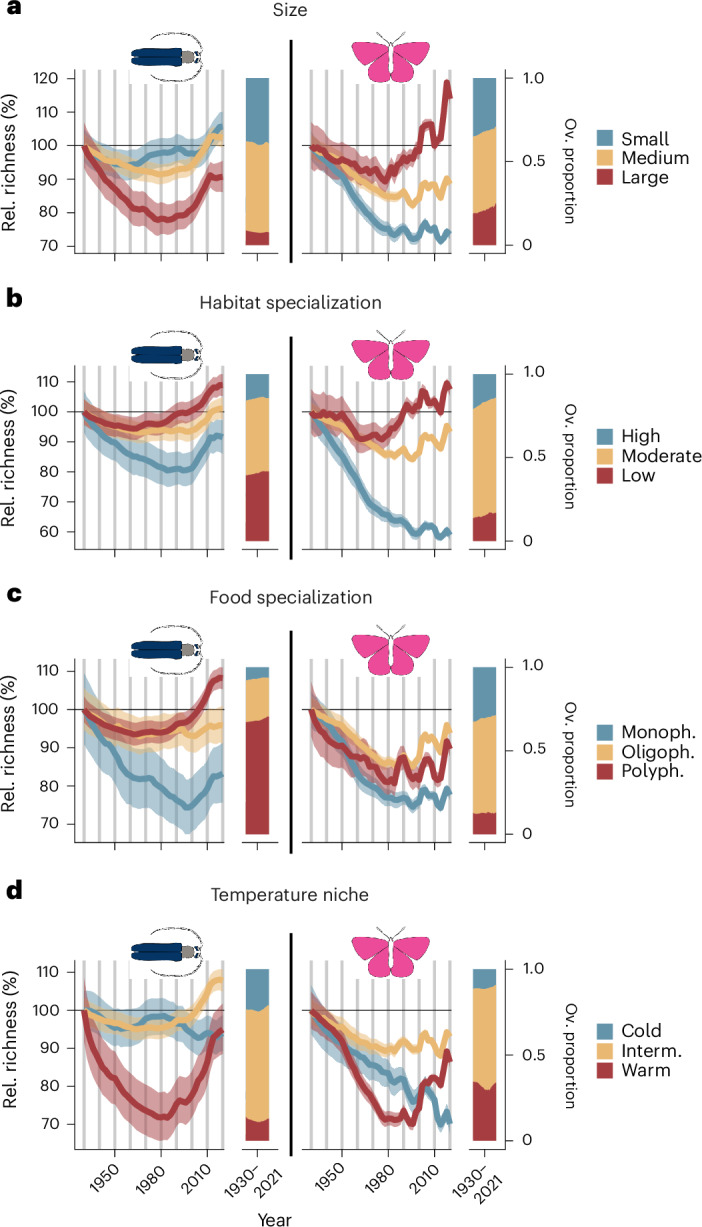
Change in saproxylic beetle and butterfly species richness since 1930, distinguished by traits. **a**–**d**, The line graphs show the trajectories of average species richness per 5 km × 5 km square relative to the 1930 value (per trait value) for saproxylic beetles and butterflies for the whole of Switzerland, separated by different traits: size (**a**), habitat specialization (**b**), food specialization (**c**) and temperature niche (**d**), and trait values (colours). Lines represent means; shaded areas indicate 95% CIs. Vertical lines mark decade boundaries. The bar plots on the right of each panel show how the proportions of species with different trait values within the overall richness have changed between 1930 (left) and 2021 (right). Rel., relative; Ov., overall; Monoph., monophagous; Oligoph., oligophagous; Polyph., polyphagous; Interm., intermediate.

Global change affects insect species unevenly, creating winners and losers^33^. Using a trait-based approach to analyse temporal trends in species richness provides insight into the causes of these changes and their functional consequences^24^. We found that changes in species richness varied among groups of species with different trait values (Fig. 4). The average richness of large saproxylic beetles was −9.2% (95% CI: −15.8% to −2.2%) lower at the end of the study period than in 1930, whereas the richness of small saproxylic beetles remained similar to that in 1930 (Fig. 4a). Large saproxylic beetles have been affected more by changes in forest management^36,37^, for example, due to their requirement for larger deadwood diameters—a resource that is more prevalent in old-growth forests, which have become rare^29,38^. By contrast, small butterfly species declined more than large species (a −26.4% (95% CI: −29.7% to −22.8%) decrease versus a 14.1% (95% CI: 6.3% to 22.5%) increase). Butterfly body size is a good indicator of dispersal ability^39^, and global change drivers such as land-use intensification are known to harm species with lower dispersal ability disproportionately^40^. Thus, the decline in small butterfly species over the past 90 years may be linked to their lower dispersal ability, which is becoming an increasingly significant disadvantage in a rapidly changing world.

**Extended Data Fig. 1:**
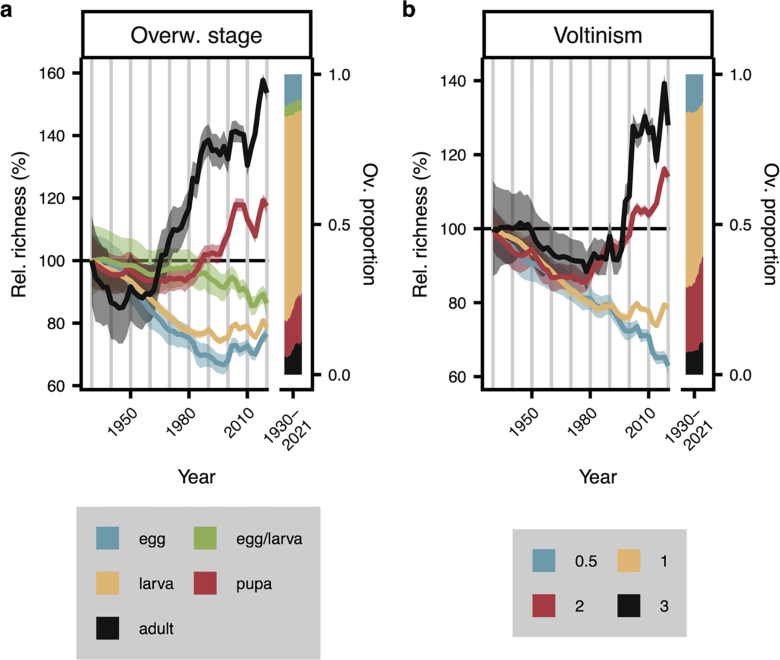
Change in butterfly species richness since 1930, distinguished by overwintering stage and voltinism (number of generations per year). The line graphs show the trajectories of average species richness per 5 km × 5 km square relative to the 1930 value (per trait value) for the whole of Switzerland, separated by overwintering stage (**a**) and voltinism (**b**). Lines represent means, shaded areas indicate 95% credible intervals. Vertical lines mark decade boundaries. The bar plots on the right of each panel show how the proportion of species with different trait values within the overall richness has changed between 1930 (left) and 2021 (right).

For the other traits considered in both species groups (habitat specialization, food specialization and temperature niche), the differences in overall changes between trait values largely matched between saproxylic beetles and butterflies (Fig. 4b–d). Over the full study period, the greatest decreases in richness were observed among habitat specialists, with the average richness of highly specialized species decreasing by −8.4% (95% CI: −14.9% to −1.3%) for saproxylic beetles and −41.0% (95% CI: −44.5% to −37.4%) for butterflies (Fig. 4b). At the same time, the average richness of habitat generalists remained largely constant across the study period. The clear decreases in the richness of habitat specialists, particularly at the beginning of the study period, are consistent with previous studies on saproxylic beetles^29,36^ and butterflies^9,24^. These decreases reflect the degradation and loss of suitable habitats for many species, such as old-growth forests^38^ and semi-natural grasslands^41^. Furthermore, land-use intensification and eutrophication have contributed to the decline of specialist species^40,42^. The important roles of land-use intensification and eutrophication in community restructuring are also reflected in the clear declines of butterfly species with fewer generations per year and that overwinter in earlier developmental stages (Extended Data Fig. 1), which are known to be more strongly affected by these drivers^40,43^.

Similar to the pattern observed for habitat specialization, the richness of food-specialized species decreased the most over the entire study period. The decrease in average richness of monophagous species was −16.6% (95% CI: −25.2% to −7.3%) for saproxylic beetles and −22.3% (95% CI: −26.1% to −18.4%) for butterflies (Fig. 4c). Decreases among food-specialized butterflies have previously been observed^22^. These decreases reflect the loss of food plants specific to these species, due to the impoverishment of plant communities in open habitats^44^, which is a consequence of land-use change and intensification^45^, increasing atmospheric nitrogen deposition^46^ and the homogenization of plant communities^47^. Approximately half of the studied monophagous saproxylic beetle species are specialized on *Pinus*, *Populus* or *Quercus*. Species from these genera probably decreased in number until the mid-twentieth century owing to more intensive forest management (*Pinus*, *Quercus*)^48^ and the loss of floodplain forests (*Populus*)^49^, which would explain the decline of many monophagous species. Decreases in specialist insects were clear and prevalent irrespective of insect group or specialization axis, reflecting losses of habitats and resources over the past century.

Finally, clear differences in temporal patterns emerged between species with different temperature niches (Fig. 4d). In both saproxylic beetles and butterflies, we observed the strongest decreases in warm-adapted species during the first 50 years, which can be attributed to their greater prevalence in the intensively used lowland regions. However, around 1980, this trend reversed, with the richness of warm-adapted species increasing in both insect groups. By 1980, the average richness of warm-adapted species had fallen by −28.1% (95% CI: −34.3% to −21.4%) for saproxylic beetles and −28.4% (95% CI: −32.1% to −24.5%) for butterflies. By 2020, these declines had somewhat recovered, resulting in decreases of only −4.9% (95% CI: −12.9% to 3.9%) for saproxylic beetles and −13.5% (95% CI: −17.8% to −9.1%) for butterflies over the entire study period. This increase after 1980 coincided with the acceleration of global warming, which began in the 1980s^25^ (Supplementary Fig. 3). Unlike warm-adapted species, the richness of cold-adapted species decreased more consistently (Fig. 4), particularly among butterflies, for which the average richness at the end of the study period was −29.8% (95% CI: −34.8% to −24.7%) lower than at the beginning. The trend reversal in the 1980s was also apparent for butterfly species with two or more generations (Extended Data Fig. 1b), which generally benefit from a warming climate^50^. Our findings support climate-driven restructuring of insect communities^23,24^, which seems to have become prevalent since the acceleration of climate warming in the 1980s.

**Extended Data Fig. 2:**
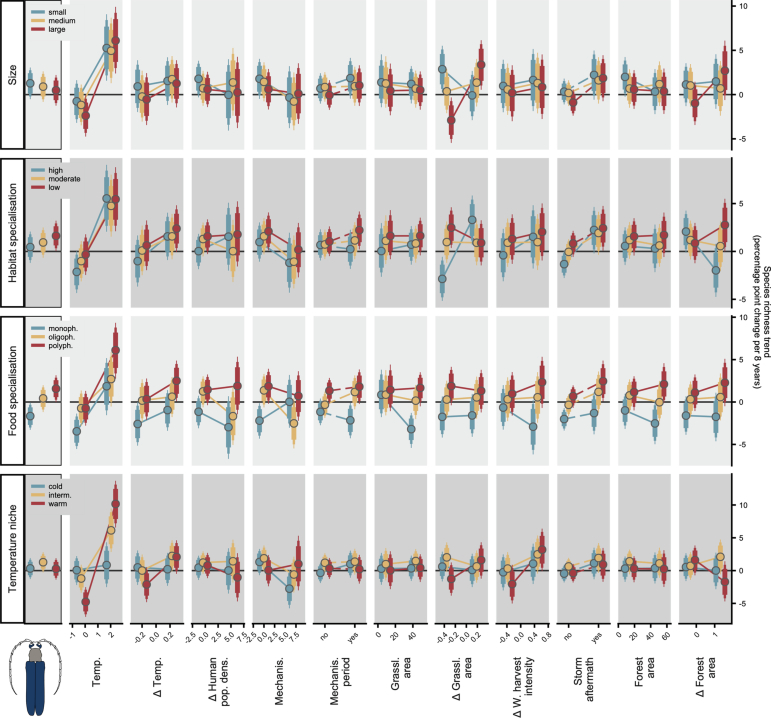
Predictions from models relating saproxylic beetle occupancy changes to environmental variables and traits. Regression model predictions for models relating the trend of relative richness of saproxylic beetles (separated by different trait values) in a biozone and eight-year interval to the environmental conditions and changes therein in the same zone, eight-year interval and to different trait values. Also, models included interactions between all environmental variables and the trait values. One model was fitted for each trait (rows). The predictions show the expected richness trend. The panels at the very left show the main effect of the different trait values, the remaining panels illustrate the interactive effects between trait value and environmental variables. Points show means, segments of varying thickness show 80%-, 90%- and 95%-credible intervals. *n* = 270 observations (biozone-interval-traitvalue combinations) per model. Temp.: temperature absolute, Δ Temp.: temperature change; Δ Human pop. dens.: Human population density change, Mechanis: mechanisation absolute, Mechanis. period: Mechanisation period (factor), Grassl. area: grassland area absolute, Δ Grassl. Area: grassland area change, Δ W. harvest intensity: wood harvest intensity change.

**Extended Data Fig. 3:**
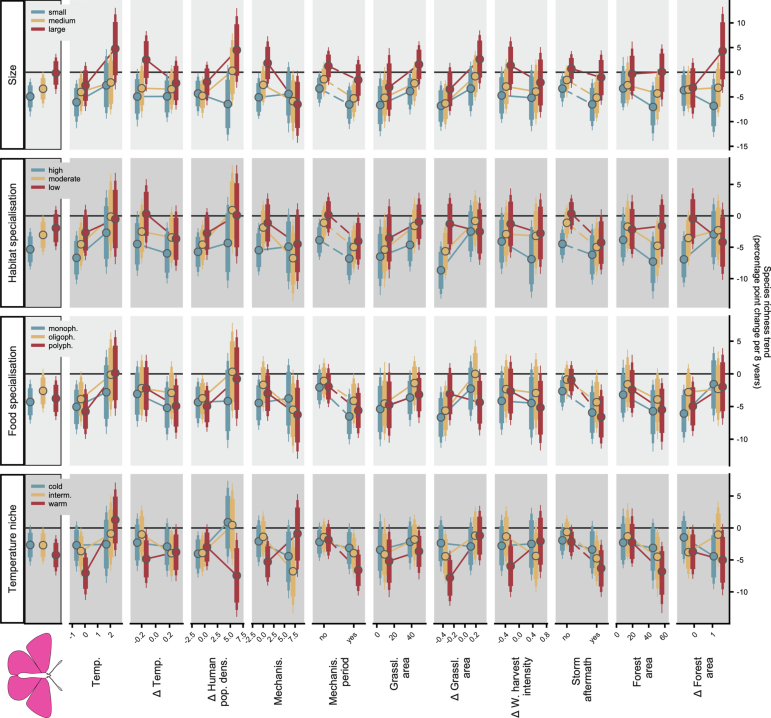
Predictions from models relating butterfly occupancy changes to environmental variables and traits. Regression model predictions for models relating the trend of relative richness of butterflies (separated by different trait values) in a biozone and eight-year interval to the environmental conditions and changes therein in the same zone, eight-year interval and to different trait values. Also, models included interactions between all environmental variables and the trait values. One model was fitted for each trait (rows). The predictions show the expected richness trend. The panels at the very left show the main effect of the different trait values, the remaining panels illustrate the interactive effects between trait value and environmental variables. Points show means, segments of varying thickness show 80%-, 90%- and 95%-credible intervals. *n* = 270 observations (biozone-interval-traitvalue combinations) per model. Temp.: temperature absolute, Δ Temp.: temperature change; Δ Human pop. dens.: Human population density change, Mechanis: mechanisation absolute, Mechanis. period: Mechanisation period (factor), Grassl. area: grassland area absolute, Δ Grassl. Area: grassland area change, Δ W. harvest intensity: wood harvest intensity change.

To investigate the relationship between species traits and drivers of global change further, we used regression models similar to those used above, but with a separate model for each trait, which incorporated an interactive effect between environmental variables and trait values. These interactions would show that species characterized by certain trait values are more or less sensitive to specific environmental conditions or changes therein. For instance, cold-adapted species should be more adversely affected by climate warming^23^. Indeed, we found that the generally positive relationship with absolute temperature did not apply to cold-adapted saproxylic beetle and butterfly species (Extended Data Figs. 2 and 3). Furthermore, we found this interactive effect not only for absolute temperature, but also for temperature change. Warm-adapted species in both groups showed more positive trends during intervals of greater temperature increase (Extended Data Figs. 2 and 3). Interestingly, the negative relationship with absolute mechanization did not apply to warm-adapted saproxylic beetle and butterfly species (Extended Data Figs. 2 and 3). Two main processes that can result in warmer microclimatic conditions more suitable for warm-adapted species may explain this. Firstly, higher mechanization of agricultural land use is associated with a lower proportion of structural landscape elements, such as hedges^31^. Secondly, mechanization in forest management may result in younger stands with lower canopy closure owing to regular management activities^29^. Overall, our findings clearly show that species adapted to intermediate and warm temperatures could benefit directly from a warming climate, as well as potentially from land-use change. By contrast, species adapted to colder conditions could not benefit or were harmed.

By reconstructing the temporal trajectories of species richness for functionally distinct groups of insects across a large environmental gradient and over almost a century, we provide insights into insect declines during a crucial time period, which has largely been missing from previous analyses. Our results show that insect declines were sharpest around the mid-twentieth century, shifting the baseline for studies on changes in insect diversity that start in the late twentieth century. This should be more explicitly acknowledged when interpreting insect trends over just the past few decades. While the mechanization of agricultural management between the 1950s and 1980s—potentially accompanied by mechanization of forest management—was associated with pronounced declines in insect richness, changes towards more biodiversity-friendly management in recent decades have probably contributed to the stabilization of butterfly richness trends and the recovery of saproxylic beetle richness. Further transitioning to biodiversity-friendly management, particularly in agriculture, will be key to increasing insect richness and restoring it to levels before large-scale agricultural mechanization. The ongoing decline of specialist butterfly species in recent decades highlights that specialist insect species should be a particular focus of conservation efforts, for example, through the restoration of suitable habitats and landscape connectivity^34^. At the same time, climate warming has clearly restructured insect communities since the 1980s. Targeted efforts will be required to conserve cold-adapted species, for example, by protecting their alpine habitats. Managing landscapes in a changing world to support a high diversity of functionally distinct insect groups will be challenging, but necessary for maintaining and promoting functional ecosystems in the future.

## Methods

We performed all statistical analyses with R version 4.0.2 (ref. ^51^) and higher and Stan version 2.27.0 (ref. ^52^) and higher. Besides the explicitly mentioned packages, the R packages cowplot^53^, data.table^54^, DHARMa^55^, ggh4x^56^, ggpubr^57^, giscoR^58^, lubridate^59^, ncdf4^60^, posterior^61^, raster^62^, sf^63^, sfheaders^64^, stars^65^, tidyverse^66^ and zoo^67^ were used for data handling, data analysis and plotting. To summarize posterior distributions from Bayesian models, we used means and CIs. We calculated CIs with the package bayestestR^68^.

### Study region

The study includes species records from Switzerland, which is situated along the Alps in Central Europe (Fig. 1a). Switzerland covers 41,285 km^2^ and an elevational gradient ranging from 193 m to 4,634 m a.s.l. Based on biogeographic regions^21^ and elevation, we divided the country into six biozones, which were characterized by distinct ecological communities, environmental conditions and trajectories over the past century (Fig. 1a). Out of the six biogeographic regions of Switzerland, which are defined from the distribution of flora and fauna while accounting for institutional borders^21^, we pooled the two central alpine regions to have higher numbers of records per biozone. The five resulting biozones were the following: the mountainous Jura biozone in the northwest is characterized by high shares of managed grasslands and also forests, and experienced a significant intensification of agriculture in the past century (Fig. 1b and Supplementary Figs. 4, 6 and 7). The lowland plateau biozone, where most of the human population and intensive agriculture are based, has comparatively low forest cover. It experienced strong increases in human population and agricultural intensity in the past century (Fig. 1b and Supplementary Figs. 4 and 6–8). The mountainous northern Alps biozone includes the northern Prealps, with high shares of grassland-based agriculture, which was significantly intensified in the past century (Fig. 1b and Supplementary Figs. 4 and 6). The central Alps biozone includes the inner alpine valleys with more continental climate, where managed grasslands and forests are prevalent, while agricultural intensification was less evident compared with the three northern biozones (Fig. 1b and Supplementary Figs. 4, 6 and 7). The southern Alps biozone entails the southern Prealps, which are climatically influenced by Mediterranean climate. Forest shares increased strongly in this biozone in the past century, while managed grasslands have clearly decreased and agricultural intensification was less evident than in the regions north of the Alps (Fig. 1b and Supplementary Figs. 4, 6 and 7). To account for elevational differences in insect communities and environmental conditions, we defined an additional high Alps biozone as the area that lies above 1,400 m a.s.l. We chose the elevational threshold such that biozones covered contiguous areas with similar environmental conditions and also entail high-enough species record numbers for analyses. The high Alps biozone is the least densely populated zone and agricultural use is clearly lower compared with that of the low-elevation biozones, except for relatively high shares of grasslands that are used as summering pastures. Forest area is comparably low, as a large share of the area is located above the tree line (Fig. 1b and Supplementary Figs. 4 and 6–8).

### Species records data

We retrieved records data from 1930 to 2021 of 1,439 beetle species of 52 taxonomic families that contain saproxylic species (Supplementary Table 1) and of 241 butterfly species (including few species aggregates; butterfly refers here to Papilionoidea and Zygaenidae moths) (Supplementary Table 2) from the database curated by info fauna (The Swiss Faunistic Records Centre; metadata available from the Global Biodiversity Information Facility (GBIF) through 10.15468/cjmjy2 and 10.15468/atyl1j). This database contains species records from projects such as research projects or red list inventories as well as from naturalists, museum collections and private collections. We included only records for which precision of the spatial information was at least 2,500 m. We used the spatial information to attribute each record to a 5 km × 5 km square (henceforth ‘square’), which we used as spatial observational unit in the analyses (Fig. 1a). We retrieved 375,713 beetle records and 2,165,090 butterfly records. Besides information on species and on location, each record contained information on the observer or project identity, on whether the observation originated from a digitized museum collection, and on the trapping method if records originate from trapping activities. The latter was available and relevant only for beetles, which are regularly collected using traps, while butterflies are generally observed directly in the field. We pooled trapping methods into the following categories: emergence traps, flight-interception traps, ground traps, light traps and other traps. To account for low numbers of records in early years (Supplementary Fig. 9), we used intervals of two consecutive years (1930–1931, 1932–1933 and so on) as temporal units in the models for occurrence probability (see below).

Next, we attributed each record to a visit, which we used as the spatiotemporal replication unit in the models for detection probability (see below). For butterflies, we defined a visit by a unique combination of square, observation date and observer (project, if available; otherwise, person). For saproxylic beetles, a considerable amount of data was based on samples from traps (25.2% of the records of the final dataset). For these, information on the exact sampling date was often missing because traps were active over several days or weeks. Furthermore, several traps might have been active within a single square. Thus, we chose a more elaborate definition of a visit for saproxylic beetle records from trapping. On the basis of the spatial precision of each record (ranging between 1 m and 2,500 m) and the spatial and temporal information, we built spatiotemporal clusters of records (that is, records from within a square that overlap temporally and spatially based on the recorded precision). We defined a visit as a unique combination of square, spatiotemporal cluster, observer (project, if available; otherwise, person) and trapping method. If records were not from trapping, we used the same definition of a visit as for butterflies (that is, unique combination of square, observation date and observer).

Before the analyses, we excluded from both datasets records from squares for which only records from a single 2-year interval were available^69^ (587 beetle records, 109 butterfly records). Furthermore, the datasets contained many instances of multiple records per species and visits, which we excluded from analyses (81,517 beetle records, 1,181,366 butterfly records). Finally, in recent years, there was a clustering of observers (projects or naturalists) with extraordinarily high numbers of species recorded per visit evident from the beetle dataset. This resulted in an increase of the average number of species recorded per visit in recent years, which might lead to biased analyses (Supplementary Fig. 10). Thus, we excluded the records from observers with an average number of species per visit above the 97.5% quantile (after exclusion of observers with only one visit) (57,444 records), which removed the bias in average species number per visit in recent years (Supplementary Fig. 10). The final datasets contained 236,165 beetle records (from 1,500 squares) and 983,615 butterfly records (from 1,719 squares) (Supplementary Fig. 9).

### Occupancy-detection models

Based on these species records, we estimated the mean occupancy (that is, the average number of squares occupied) per 2-year interval and biozone for each species using occupancy-detection models^15,70^. We followed the approaches used in refs. ^24,69^. In occupancy-detection models, the occurrence and detection probabilities are modelled simultaneously with two hierarchical models to correct for observation bias in the records data. We fitted separate models for all species recorded in at least 25% of 2-year intervals. We did not fit models for non-saproxylic beetle species (Supplementary Table 1), although these were used to determine non-detections of other beetle species (see below), and for some butterfly species recorded with insufficient taxonomic identification (Supplementary Table 2). In total, we fitted 595 models for saproxylic beetle species and 216 models for butterfly species, covering a large part of the extant species in Switzerland (approximately 1,000 strictly saproxylic species and 230 butterfly species have been recorded).

The model for occurrence probability included fixed and random terms for elevation, the 12 fine biogeographic regions of Switzerland (defined from floristic and faunistic distributions, while following institutional borders^21^), the square and the 2-year interval. In the model for detection probability, we accounted for the thoroughness of the observation through the number of species recorded, the scope and expertise of the observer, the type of observation (for example, trap type; only for saproxylic beetles), the origin of the data (from a museum or not), the observer identity and the 2-year interval of the observation.

We implemented the occupancy-detection models in Stan and fitted them using cmdstanr^71^. On the basis of occurrence probability estimates, we determined mean occupancy per species, 2-year interval and biozone. These estimates were used for further analyses. While we based our modelling approach on approaches that were shown to be robust^24,69,72^, we further validated the estimated occupancies with additional available data. First, we compared linear 90-year trends per species to their red list status for species that were assessed in the available Swiss red lists^73,74^ (198 saproxylic beetle and 214 butterfly species). We found red list status and species trends to be clearly related (Supplementary Fig. 1). Second, we compared butterfly species trends based on occupancy-detection models with species trends based on a standardized, Switzerland-wide sampling scheme, which has been in place since 2003. For the overlapping 19 years, we found very high alignment between the trends based on the two different data sources (Supplementary Fig. 2). A more detailed description of the modelling approach can be found in Supplementary Methods.

### Species traits

To analyse how species richness trends depended on species traits, we categorized all study species according to a set of categorical traits. For saproxylic beetles, we extracted a set of morphological and ecological traits from ref. ^75^ and filled gaps based on refs. ^36,76^. We used data on body length (continuous), moisture preference (three-level factor: xerophilous, mesophilous, hygrophilous), sunlight preference (three-level factor: sciaphilous, unspecific, heliophilous), tree-group preference (five-level factor: coniferous, mostly coniferous, mixed, mostly deciduous, deciduous), deadwood-diameter preference (three-level factor: thin (that is, <10 cm), unspecific, thick (that is, >40 cm)) and on recorded hosts (deadwood tree species). We converted body length to a categorical body size (three-level factor: small, medium, large) based on equally distanced breaks (previous log transformation). From the four habitat-related traits, we determined a categorical habitat specialization trait for each species: we counted the number of specialized trait values (that is, xerophilous and hygrophilous for moisture preference, sciaphilous and heliophilous for sunlight preference, coniferous and deciduous for tree-group preference, thin and thick for deadwood-diameter preference). We defined species with specialized trait values in all four habitat-related traits as species with high habitat specialization (stenotopic), species with three specific trait values as species with moderate habitat specialization (oligotopic) and all other species as species with low habitat specialization (eurytopic). Furthermore, we categorized species by food specialization. We considered species recorded from plant hosts of more than one taxonomic family as polyphagous (low food specialization), species recorded from hosts of more than one genus as oligophagous (medium food specialization) and species recorded from hosts of only one genus as monophagous (high food specialization).

For butterflies, we extracted traits from ref. ^20^ and filled gaps based on some additional sources^77–79^ and expert knowledge. We used data on wing length, which we transformed to categorical body size (three-level factor: small, medium, large) based on equally distanced breaks (previous log transformation), on habitat specialization (three-level factor: high, moderate, low; reflecting the three levels stenotopic, oligotopic and eurytopic), and on food specialization (three-level factor: monophagous, oligophagous, polyphagous) as main traits. In addition, we included data on hibernation stage (five-level factor: egg, egg and larva, larva, pupa, adult) and voltinism (four-level factor: 0.5, 1, 2, 3 generations per year).

To estimate temperature preferences of all study species, we determined temperature niches for each species following the approach of the species temperature index^80^. To that end, we extracted records for all saproxylic beetle study species from the GBIF database (GBIF.org, 10.15468/dl.mak332). We restricted records to Europe and aggregated them at the Common European Chorological Grid Reference System (CGRS) grid. At the same grid, we aggregated mean temperature data (1970–2000) at 2.5 min spatial resolution from WordClim 2 (ref. ^81^). Then, we calculated each species’ temperature niche as the mean temperature of the CGRS grid cells that it was recorded from. For butterflies, we used temperature niches determined with the same approach in an earlier analysis^24^. We determined the categorical temperature niche (three-level factor: cold, intermediate, warm) based on equally distanced breaks within each of the two groups.

### Species richness

For both saproxylic beetles and butterflies, we used the posterior distributions of the mean occupancy per biozone and 2-year interval to identify variables of community-wide changes. First, we determined a proxy for average expected species richness per square and 2-year interval as the sum of all species’ mean occupancies per biozone and also for the whole of Switzerland. On the basis of 5,000 Monte Carlo simulations, we summarized the various posterior distributions with means and 95% CIs. Also, we used the same approach for species subsets defined by the different categorical traits to identify whether trajectories of species richness differed between species characterized by different traits. To enable better comparison of the results, we calculated richness values relative to the point estimate of species richness per functional group (and trait value if applicable) for the whole of Switzerland in 1930 (that is, percentage richness compared with the starting values in an average square in the whole study region). We report (changes in) these relative richness values throughout.

To analyse changes in relative species richness and their relationship to (changes in) environmental conditions, we divided the study period into 15 consecutive 8-year intervals (1930–1937, 1936–1943, …, 2014–2021). The overlap of consecutive intervals is a consequence of the 2-year intervals at which richness was estimated. For example, the first 8-year interval of richness estimates ends with 1936–1937 and the second starts with 1936–1937 to avoid gaps between consecutive intervals. The length of the intervals is short enough to track variability in environmental conditions (Supplementary Figs. 3–8) and long enough to cover insect community changes^3,82^. To check the sensitivity of our results to the choice of the length of the interval, we additionally used 9 12-year intervals (1930–1941, 1940–1951, …, 2010–2021). For each interval and zone, we determined the change in relative richness with linear models (relative richness ~ year). We extracted slope estimates for the year for all 5,000 Monte Carlo simulations and used point estimates (means) for further analyses. In addition, we used the same approach to determine richness trends for each interval and biozone for each species subset defined by the different categorical traits.

### Environmental variables

For the whole study period, we reconstructed environmental conditions and changes therein, with a special focus on the global change variables for which we expected the strongest relations to occupancy changes during the study period, that is, climate change and land-use change^3,24,83–85^. For land-use variables, we focused on variables related to forests and agriculturally managed open lands, which represent the major habitats of our two study groups (saproxylic beetles, butterflies). We quantified all variables for each biozone and for the same 8-year intervals (and 12-year intervals), for which occupancy changes were determined. An overview and detailed description of the variables and the underlying data are given in Supplementary Table 3.

In terms of climate change, we focused on mean annual temperature anomaly and how it changed, as average temperature is crucially linked to the distributions of insect populations^86^. Also, mean temperature has clearly changed over the studied period in Switzerland (Supplementary Fig. 3), which is why we expected it to be an important driver of community changes. To account for lag effects in range shifts^87^, we included both the change in mean annual temperature anomaly per 8-year interval (‘temperature change’) and the mean annual temperature anomaly of each 8-year interval (‘temperature absolute’) (Supplementary Table 3 and Fig. 3). As a general indicator of increasing anthropogenic pressures on the studied ecosystems, we included the change in human population density per biozone and 8-year interval (‘human population density change’; Supplementary Table 3 and Fig. 8). This variable may be indicative of several processes, such as increasing urban sprawl, which we expected to affect insect community changes^88^.

Open-land habitats such as grasslands, which are particularly important for butterflies^30^, are strongly shaped by agricultural use in Switzerland. We included the mean amount and the change of grassland area per biozone and 8-year interval as indicators of habitat availability and change therein (‘grassland area absolute’ and ‘grassland area change’; Supplementary Table 3 and Fig. 6). We not only accounted for changes but also considered absolute values, as we expected persistently low amounts of habitats to negatively affect populations, particularly owing to fragmentation^89^. During the studied period, agriculture was strongly intensified in Switzerland and elsewhere^13^. Among other changes, mechanization of agricultural management greatly changed agroecosystems^31,90^. We determined the number of tractors that were used in agriculture as a proxy for mechanization across the whole study period and used the mean amount and the change therein per biozone and 8-year interval as variables of agricultural intensification (‘mechanization absolute’ and ‘mechanization period’; Supplementary Table 3 and Fig. 4). We implemented mechanization period as a factor variable, as a clear transition period from the 1950s to the 1980s was apparent from the observed changes (Supplementary Fig. 4). Simultaneously to the increasing mechanization, other changes in agricultural practices such as increasing fertilizer and pesticide use or increasing farm and field sizes took place in Switzerland and elsewhere^13,31^, which we did not quantify directly.

Forest habitats are particularly important for saproxylic beetle species, which depend on deadwood or specific structures associated with deadwood to complete their life cycles. We included the mean amount and the change of forested area per biozone and 8-year interval as indicators of habitat availability and change therein (‘forest area absolute’ and ‘forest area change’; Supplementary Table 3 and Fig. 7). As for grasslands, we included not only change in forest area but also the absolute values to account for the persistent effect of both low or high habitat amounts. As a proxy of forest management intensity, which is known to affect saproxylic beetle communities, particularly through changes in deadwood availability^91^, we determined the amount of wood harvested per forest area. We chose to include only the change in wood harvest intensity (‘wood harvest intensity change’; Supplementary Table 3 and Fig. 5), as this proved to be most indicative of changes that we observed during the study period, such as the increase of wood harvest intensity in Switzerland during World War II (Supplementary Fig. 5). Increases in wood harvest intensity probably contributed to a significant reduction in the deadwood available to saproxylic beetle communities. In addition, we observed strong outliers in wood harvest intensity, which we could link to extraordinary storm events with high amounts of windthrow (Supplementary Fig. 5). These windthrow events and the resulting changes to deadwood amount and forest structure (for example, light conditions) are known to strongly affect forest insect communities such as saproxylic beetles^35^. For this reason, we included a factor denoting the 8-year intervals following a storm event with high amounts of windthrow (‘storm aftermath’; Supplementary Table 3 and Fig. 5).

To compare the different variables before and after modelling (see below), we scaled all continuous variables to a standard deviation of 0.5, which allows a better comparison of factors and continuous variables^92^.

### Regression models

We used regression models to relate the richness trends (average linear model slope) to the selected environmental change variables at the level of the 6 biozones and the 15 8-year intervals (1930–1937, 1936–1943, …, 2014–2021; the overlap is because of richness estimates for 2-year intervals) (*n* = 90). We fitted one model per studied group (saproxylic beetles, butterflies). Besides the 11 environmental change variables (Fig. 1b; all absolute pairwise correlation coefficients between continuous variables were below 0.7 (ref. ^93^)), which we included as fixed terms, we included random terms for the 8-year interval (15 levels) and the biozone (6 levels). To analyse whether species with different trait values responded differently to the various environmental changes, we used richness trends per 8-year interval and biozone for each categorical trait value (*n* = 270 for traits with 3 trait values). We restricted these analyses to the traits that are shared between the two insect groups (size, habitat specialization, food specialization, temperature niche). Then, we fitted additional models per insect group and trait with the same structure as above and additionally included the trait value and the interactions between the trait value and each environmental change variable as fixed terms. In addition, we added a random term for each 8-year interval and biozone combination (90 levels). To check whether our results are sensitive to the choice of the interval length (8 years), we used the same set of regression models, but for the longer 12-year intervals (*n* = 54 for overall models, *n* = 162 for trait models with 3 trait values). All main findings were qualitatively confirmed with the longer intervals (Supplementary Figs. 11–13).

We implemented all regression models in the package brms^94^. We used Student’s *t*-distribution as response distribution to account for underdispersion of residuals. The models ran on 4 Markov chain Monte Carlo chains with 20,000 iterations each (including 10,000 warm-up iterations). Priors are given in Supplementary Table 4. We quantified model results through the posterior distributions of different model predictions.

### Reporting summary

Further information on research design is available in the Nature Portfolio Reporting Summary linked to this article.

## Supplementary information

Supplementary Information Supplementary Tables 1–4, Figs. 1–13, Methods and References.

Reporting Summary

Peer Review File

## Data Availability

The raw records data are protected by a code of conduct, which is common to all Swiss national data centres. An anonymized version of the records data, which can be used to reproduce the occupancy-detection models, is available via Zenodo at 10.5281/zenodo.17256301 (ref. ^95^). The average occupancy estimates per species, 2-year interval and biozone are available via Zenodo at 10.5281/zenodo.17255265) (ref. ^96^). Data on traits as well as on drivers and other data necessary to reproduce the main analyses are available via Zenodo at 10.5281/zenodo.17256301 (ref. ^95^).

## Code availability

All relevant codes used for the analyses are deposited in a GitHub repository (https://github.com/nefff1/insect_trends_1930) available via Zenodo at 10.5281/zenodo.17256301 (ref. ^95^).
