# Supplementary material for: Ninety-year trends reveal sharpest insect declines in the mid-twentieth century
Source: Nat Ecol Evol. 2026 Jun 2;10(6):1103–13. doi: 10.1038/s41559-026-03074-6 (PMC13253333; doi:10.1038/s41559-026-03074-6)
Supplement: Supplementary file 1 — Supplementary Tables 1–4, Figs. 1–13, Methods and References. [file 41559_2026_3074_MOESM1_ESM.pdf]

nature ecology & evolution

Supplementary information https://doi.org/10.1038/s41559-026-03074-6

Ninety-year trends reveal sharpest insect declines in the mid-twentieth century

In the format provided by the authors and unedited

Table of contents

Supplementary Methods 2

Supplementary Tables..7

Supplementary Figures 28

Supplementary References...41

Supplementary Methods

Occupancy-detection models

Our occupancy-detection modelling approach largely followed the approaches used in Bowler et al. (2021) and Neff et al. (2022). In the latter study, occupancy-detection models were used in the same system. In the present study, we include a new insect group (while omitting others) and cover a much longer time range, which is why several adaptations to the model structures were necessary.

For the whole analyses, we treated the beetle and the butterfly datasets independently, i.e. no beetle data entered the butterfly analyses and vice versa. We fitted separate models for all species that were recorded in at least 25% of all two-year intervals. We did not fit models for non-saproxylic beetle species (Supplementary Table 1), which were however used to determine non-detections of other beetle species (see below), and for some butterfly species recorded with insufficient taxonomic identification (Supplementary Table 2). In total, we fitted 595 models for saproxylic beetle species and 216 models for butterfly species.

Occupancy-detection models are hierarchical models, in which two interconnected processes are modelled simultaneously: The ecological process, which determines the occurrence probability (i.e. probability that a species occurs in a square in a two-year interval), and the observation process, which determines the detection probability (i.e. probability that a species is detected in a visit given it is occurring). Here, the replication unit for the former is the combination of a square and a two-year interval (1500 × 46 = 69,000 for beetles, 1719 × 46 = 79,074 for butterflies), while the replication unit for the latter is a visit (95,800 for beetles, 201,972 for butterflies). In occupancy-detection models, both the occurrence and the detection probability

are modelled through logistic regressions, which can include a set of parameters that are relevant for the respective process. Here, we modelled occurrence probability $\psi_{i,t}$ of square i in the two-year interval t as follows:

$$\text{logit}(\psi_{i,t}) = \mu_o + \beta_{o1}\text{elevation}_i + \beta_{o2}\text{elevation}_i^2 + \alpha_{o1,i} + \alpha_{o2,i} + \gamma_{r(i),t} \tag{1}$$

where μ_o is the global intercept, elevation_i is the scaled elevation a.s.l. and $\alpha_{o1,i}$, $\alpha_{o2,i}$ and $\gamma_{r(i),t}$ are random effects for twelve fine biogeographic regions (defined from floristic and faunistic distributions, while following institutional borders (Bundesamt für Umwelt, 2022)), the square (up to 1500 or 1719 levels, respectively) and the two-year interval (46 levels). We used the twelve biogeographic regions instead of the six regions to better account for fine-scale variation in species occurrences. We modelled the random effects for the fine biogeographic regions and the square as

$$\alpha_{o1} \sim \text{Normal}(0, \sigma_{o1}) \tag{2}$$

and

$$\alpha_{o2} \sim \text{Normal}(0, \sigma_{o2}) \tag{3}$$

To account for dependencies in occurrence probabilities between consecutive two-year intervals, we modelled the random effect of the two-year interval with separate random walks per biozone (Outhwaite et al., 2018; Outhwaite et al., 2019). We implemented the random walks as follows:

$$\gamma_{r,t} \sim \begin{cases} \text{Normal}(0, 1.5^2) \text{ for } t = 1930/31 \\ \text{Normal}(\gamma_{r,t-1}, \sigma_{\gamma r}^2) \text{ for } t > 1930/31 \end{cases} \tag{4}$$

where

$$\sigma_{\gamma r} \sim \text{Cauchy}(0,1) \tag{5}$$

We modelled the detection probability $p_{i,t,j}$ of visit j by observer k at square i in the two-year interval t for saproxylic beetles as follows:

$$\begin{aligned}\text{logit}(p_{i,t,j}) = \; & \mu_d + \beta_{d1}\text{singlelist}_j + \beta_{d2}\text{listlength}_{\text{focal}_j} + \beta_{d3}\text{listlength}_{\text{nonfocal}_j} + \\ & \beta_{d4}\text{expert}_k + \beta_{d5}\text{project}_k + \beta_{d6}\text{expert}_{\text{project}_k} + \beta_{d7}\text{targeted}_{\text{project}_k} + \beta_{d8}\text{redlist}_k + \\ & \beta_{d9}\text{trap}_{emergence_j} + \beta_{d10}\text{trap}_{flight_j} + \beta_{d11}\text{trap}_{ground_j} + \beta_{d12}\text{trap}_{light_j} + \beta_{d13}\text{trap}_{other_j} + \\ & \beta_{d14}\text{museum}_j + \\ & \alpha_{d1,t} + \alpha_{d2,k}\end{aligned} \quad (6)$$

where μ_d is the global intercept, singlelist_j is a dummy of a two-level factor denoting whether only one species was recorded in the visit, $\text{listlength}_{\text{focal}_j}$ is the natural logarithm (after adding 1) of the number of species recorded in the focal taxonomic group, and $\text{listlength}_{\text{nonfocal}_j}$ is the natural logarithm (after adding 1) of the number of species recorded outside of the focal taxonomic group. We built taxonomic groups based on superfamilies and families (Supplementary Table 1), with the aim to have reasonably large groups of species which are more probable to be recorded in combination, e.g. if a naturalist preferably records cerambycid beetles. The parameters expert_k, project_k, $\text{expert}_{\text{project}_k}$, $\text{targeted}_{\text{project}_k}$ and redlist_k are dummies of a six-level factor, which categorises the observation: a common naturalist observation by a private person (reference level), an observation by an expert naturalist, an observation from a project, an observation from an expert project, an observation from a project targeted at the focal species or an observation from a Red-List inventory. We defined expert naturalist and expert projects as observers that contributed a significant share of the records. Expert naturalists and expert projects had to fulfil two criteria. They had to be (i) in the 2.5% quantile of all observers (naturalists or projects) arranged by their total number of records and (ii) they had to have made at least one visit with an exceptionally long species list (upper 2.5% quantile of all visits arranged by number of records) (Neff et al., 2022). The parameters $\text{trap}_{emergence_j}$, trap_{flight_j}, trap_{ground_j}, trap_{light_j} and trap_{other_j} are dummies from a six-level factor denoting the sampling method, which might either be none (reference level; no specific trapping

method indicated) or a trap type (emergence, flight interception, ground, light, other). The parameter museum_j denotes whether the record originates from a digitised museum record. $\alpha_{d1,t}$ and $\alpha_{d2,k}$ are random effects for the two-year interval (46 levels) and for the observer identity (observers with fewer than 15 visits were grouped; up to 369 levels for beetles and up to 1260 levels for butterflies, respectively), which we modelled as

$$\alpha_{d1} \sim \text{Normal}(0, \sigma_{d1}) \tag{7}$$

$$\alpha_{d2} \sim \text{Normal}(0, \sigma_{d2}) \tag{8}$$

For the detection probability of butterfly species, we chose a different model specification for the list length (number of species recorded in a visit) because the group is more homogeneous and contains fewer species compared to the saproxylic beetles. Also, we did not include trapping method, as butterflies were generally directly observed on site. The model looked as follows:

$$\begin{aligned} \text{logit}(p_{i,t,j}) = \; & \mu_d + \beta_{d1}\text{singlelist}_j + \beta_{d2}\text{shortlist}_j + \beta_{d3}\text{longlist}_j + \\ & \beta_{d4}\text{expert}_k + \beta_{d5}\text{project}_k + \beta_{d6}\text{expert}_{\text{project}_k} + \beta_{d7}\text{targeted}_{\text{project}_k} + \beta_{d8}\text{redlist}_k + \\ & \beta_{d9}\text{museum}_j + \\ & \alpha_{d1,t} + \alpha_{d2,k} \end{aligned} \tag{9}$$

where parameters are the same as in equation (6) and shortlist_j and longlist_j are dummies of a three-level factor, which denotes the number of species that were recorded during the visit (1 is the reference level; 2–3 is a short list; >3 is a long list).

For each analysed beetle and butterfly species, we fitted one occupancy-detection model. Species records entered the models as detection/non detection data $y_{i,t,j}$ at square *i* in year *t* at visit *j* for the focal species. We assumed non detection for each visit in which the focal species was not recorded. We only included visits that had a restricted taxonomic focus (targeted projects) for species that fell within that taxonomic focus. Furthermore, we did not include data from

biozones in which the focal species was never recorded. Detection/non detection data $y_{i,t,j}$, occurrence probability $\psi_{i,t}$ and detection probability $p_{i,t,j}$ were linked through

$$z_{i,t} \sim \text{Bern}(\psi_{i,t}) \tag{10}$$

and

$$y_{i,t,j}|z_{i,t} \sim \text{Bern}(z_{i,t}p_{i,t,j}) \tag{11}$$

where $z_{i,t}$ is the latent occupancy state of a square *i* at the two-year interval *t*, which can either be 1 (present) or 0 (absent). For each species, we used the posterior distribution of the average occupancy of squares per biozone and two-year interval for the subsequent analyses.

We implemented the occupancy-detection models in Stan and fitted them with the interface cmdstanr (Gabry et al., 2021). We used four Markov chain Monte Carlo chains with 2000 iterations each (including 1000 warm-up iterations). An overview of the priors of the various model parameters is given in Supplementary Table 4. We used the Rhat statistic to ensure that chains mixed well, which we determined for mean occupancy estimates per biozone and two-year interval with the package rstan (Guo et al., 2020). For saproxylic beetles (n = 143,198), 89.0% of Rhat values met the 1.1 threshold (99.9% of values < 1.49 and all values < 1.70). For butterflies (n = 47,768), 98.9% of Rhat values met the 1.1 threshold (99.9% of values < 1.36 and all values < 1.81).

Supplementary Tables

Supplementary Table 1 | Overview of beetle species for which records were considered in the analyses. Species are grouped by superfamily, family and focal group (latter was used in occupancy-detection models). For species in the column 'Analysed', occupancy-detection models were fitted to determine trajectories. Species in the column 'Not Analysed' were part of the analysed dataset that went into occupancy-detection models, but no models were fitted for these species. Either due to low sample sizes or because they are not categorized as being saproxylic. Numbers in brackets give the number of records that were included per species in the final dataset.

Superfamily	Family	Focal group	Analysed	Not Analysed
Bostrichoidea	Bostrichidae	Bostrichoidea	Bostrichus capucinus (135), Scobicia chevrieri (29), Xylopertha retusa (97)	Dinoderus brevis (1), D. japonicus (2), D. minutus (1), Lichenophanes varius (3), Psoa dubia (1), Rhyzopertha dominica (11), Sinoxylon anale (4), S. muricatum (2), S. perforans (5), Stephanopachys substriatus (5), Xylomeira tridens (1)
Bostrichoidea	Dermestidae	Bostrichoidea	Ctesias serra (124), Globicornis corticalis (21), G. nigripes (29), Megatoma undata (148), Trinodes hirtus (32)	Anthrenocerus australis (6), Anthrenus angustefasciatus (64), A. flavipes (5), A. fuscus (183), A. goliath (3), A. museorum (176), A. pimpinellae (95), A. scrophulariae (111), A. verbasci (293), Attagenus brunneus (2), A. pellio (266), A. punctatus (131), A. schaefferi (1), A. smirnovi (105), A. trifasciatus (42), A. unicolor (156), Dermestes ater (3), D. aurichalceus (8), D. bicolor (34), D. carnivorus (1), D. frischii (54), D. gyllenhalii (2), D. haemorrhoidalis (56), D. laniarius (112), D. lardarius (185), D. maculatus (12), D. murinus (53), D. mustelinus (1), D. olivieri (2), D. peruvianus (1), D. szekessyi (1), D. undulatus (237), Globicornis fasciata (3), G. luckowi (1), G. sulcata (1), G. tristis (1), Novelsis aequalis (1), Orphilus niger (44), Reesa vespulae (23), Trogoderma angustum (63), T. glabrum (128), T. granarium (2), T. versicolor (4)
Bostrichoidea	Nosodendridae	Bostrichoidea	–	Nosodendron fasciculare (10)

Bostrichoidea	Ptinidae	Bostrichoidea	Anobium hederae (36), A. inexspectatum (92), A. punctatum (209), Dorcatoma chrysomelina (46), D. setosella (41), Dryophilus pusillus (196), Episernus gentilis (27), E. granulatus (17), Ernobius abietinus (154), E. abietis (56), E. angusticollis (40), E. mollis (206), E. nigrinus (26), Gastrallus laevigatus (27), Grynobius planus (108), Hadrobregmus denticollis (133), H. pertinax (85), Hemicoelus canaliculatus (34), H. costatus (184), H. fulvicornis (228), Hyperisus plumbeus (63), Mesocoelopus niger (121), Microbregma emarginatum (85), Ochina ptinoides (55), Oligomerus brunneus (60), O. ptilinoides (21), Ptilinus pectinicornis (264), Ptinomorphus imperialis (217), Ptinus bidens (112), P. rufipes (81), P. subpilosus (90), Xestobium rufovillosum (35), Xyletinus ater (26)	Anitys rubens (3), Cacotemnus rufipes (6), C. thomsoni (1), Caenocara affine (4), C. bovistae (16), C. subglobosum (9), Dignomus irroratus (1), Dorcatoma androgyna (2), D. dresdensis (22), D. flavicornis (22), D. lomnickii (1), D. minor (4), D. punctulata (42), D. robusta (1), D. substriata (37), Dryophilus anobioides (10), Epauloecus unicolor (71), Episernus angulicollis (6), E. striatellus (13), E. taygetanus (2), Ernobius explanatus (1), E. freudei (1), E. kiesenwetteri (14), E. laticollis (10), E. longicornis (4), E. mulsanti (7), E. parens (1), E. pini (15), Gastrallus immarginatus (24), Gibbium psylloides (34), Homophthalmus rugicollis (9), Hyperisus declive (12), Lasioderma redtenbacheri (31), L. serricorne (99), Mezium affine (2), Nicobium castaneum (14), Niptus hololeucus (67), Ochina latreillii (9), Ozognathus cornutus (1), Priobium carpini (7), Pseudeurostus frigidus (46), Ptilinus fuscus (19), Ptinomorphus regalis (15), Ptinus aubei (8), P. bicinctus (1), P. calcaratus (1), P. capellae (2), P. catalonicus (30), P. coarcticollis (2), P. dubius (13), P. fur (316), P. latro (6), P. lichenum (16), P. pusillus (3), P. raptor (32), P. sexpunctatus (181), P. tectus (16), P. variegatus (1), Sphaericus pinguis (2), Stagetus pilula (6), Stegobium paniceum (312), Trigonogenius globosus (17), Xestobium austriacum (1), Xyletinus laticollis (22), X. longitarsis (8), X. vaederoeensis (3)

Buprestoidea	Buprestidae	Buprestidae	Acmaeoderella flavofasciata (71), Agrilus angustulus (1355), A. ater (76), A. biguttatus (478), A. convexicollis (123), A. cuprescens (244), A. cyanescens (586), A. derasofasciatus (246), A. graminis (91), A. integerrimus (83), A. laticornis (266), A. obscuricollis (103), A. olivicolor (249), A. pratensis (123), A. sinuatus (77), A. sulcicollis (680), A. suvorovi (101), A. viridis (628), Anthaxia candens (147), A. chevrieri (77), A. cichorii (62), A. fulgurans (37), A. godeti (152), A. helvetica (2084), A. hungarica (173), A. manca (67), A. morio (396), A. nitidula (1354), A. podolica (55), A. quadripunctata (2439), A. salicis (447), A. sepulchralis (123), Buprestis haemorrhoidalis (190), B. novemmaculata (136), B. octoguttata (132), B. rustica (496), Chalcophora mariana (39), Chrysobothris affinis (735), C. chrysostigma (314), C. solieri (101), Coraebus undatus (14), Dicerca alni (77), Lamprodila festiva (186), L. rutilans (183), Meliboeus fulgidicollis (42), Phaenops cyanea (439), Poecilonota variolosa (53), Ptosima undecimmaculata (97), Trachypteris picta (26)	Acmaeodera degener (2), Agrilus antiquus (3), A. auricollis (21), A. betuleti (14), A. croaticus (3), A. graecus (2), A. guerini (23), A. hastulifer (3), A. hyperici (45), A. lineola (2), A. pseudocyaneus (3), A. ribesi (13), A. roscidus (4), A. subauratus (10), A. viridicaerulans (14), Anthaxia dimidiata (1), A. istriana (20), A. millefolii (16), A. nigritula (2), A. nigrojubata (8), A. suzannae (22), Aphanisticus elongatus (47), A. emarginatus (3), A. pusillus (19), Coraebus elatus (36), C. fasciatus (41), C. rubi (17), Dicerca berolinensis (10), D. moesta (2), Eurythyrea quercus (3), Habroloma nanum (46), Lamprodila decipiens (16), L. mirifica (2), Melanophila acuminata (3), Meliboeus amethystinus (1), Phaenops formaneki (24), Sphenoptera laportei (1), Trachys fragariae (17), T. menthae (1), T. minutus (688), T. quercicola (21), T. scrobiculatus (62), T. troglodytes (65)

Chrysomeloidea	Cerambycidae	Cerambycidae	Acanthocinus aedilis (200), A. griseus (68), Acmaeops pratensis (103), A. septentrionis (105), Aegomorphus clavipes (292), Aegosoma scabricorne (384), Alosterna tabacicolor (2821), Anaesthetis testacea (294), Anaglyptus gibbosus (45), A. mysticus (586), Anastrangalia dubia (1914), A. reyi (144), A. sanguinolenta (2044), Anisorus quercus (30), Anoplodera rufipes (81), A. sexguttata (573), Arhopalus rusticus (401), Aromia moschata (1003), Asemum striatum (217), Callidium aeneum (147), C. coriaceum (168), C. violaceum (662), Cerambyx cerdo (556), C. scopolii (1375), Chlorophorus figuratus (358), C. glabromaculatus (46), C. herbstii (165), C. sartor (284), C. varius (432), Clytus arietis (3341), C. lama (526), C. tropicus (18), Cortodera femorata (153), Cyrtoclytus capra (35), Deilus fugax (77), Dinoptera collaris (1782), Ergates faber (106), Etorofus pubescens (74), Evodinus clathratus (247), Exocentrus adspersus (258), E. lusitanus (192), E. punctipennis (61), Gaurotes virginea (2209), Glaphyra umbellatarum (247), Grammoptera abdominalis (84), G. ruficornis (2595), G. ustulata (144), Herophila tristis (38), Hylotrupes bajulus (422), Judolia sexmaculata (182), Lamia textor (304), Leiopus nebulosus aggr. (989), Leptura aethiops (128), L. annularis (34), L. aurulenta (307), L. quadrifasciata (318), Lepturobosca virens (137), Menesia bipunctata (65), Mesosa curculionoides (52), M. nebulosa (381), Molorchus minor (1360), Monochamus galloprovincialis (87), M. sartor (251), M. sutor (557), Morimus asper (86), Neoclytus acuminatus (25), Oberea linearis (223), O. oculata (501), O. pupillata (301), Obrium brunneum (841), O. cantharinum (103), Oplosia cinerea (44), Oxymirus cursor (461), Pachyta lamed (46), P. quadrimaculata (1183), Pachytodes cerambyciformis (2857), Parmena balteus (159), P. unifasciata (102), Pedostrangalia revestita (35), Phymatodes rufipes (71), P. testaceus (1451), Pidonia lurida (611), Plagionotus arcuatus (752), P. detritus (83), Poecilium alni (132), Pogonocherus decoratus (61), P. fasciculatus (160), P. hispidulus (485), P. hispidus (494), P. ovatus (46), Prionus coriarius (434), Pseudovadonia livida (716), Purpuricenus kaehleri (185), Pyrrhidium sanguineum (658), Rhagium bifasciatum (369), R. inquisitor (1282), R. mordax (1197), R. sycophanta (266), Rhamnusium bicolor (44), Ropalopus clavipes	Acalolepta sejuncta (1), Acanthocinus reticulatus (13), Acmaeops marginatus (12), Agapanthia cardui (68), A. villosoviridescens (831), A. violacea aggr. (402), Anisarthron barbipes (3), Anoplophora chinensis (1), A. glabripennis (9), Arhopalus ferus (3), Brachypteroma ottomanum (15), Brachyta interrogationis (379), Calamobius filum (27), Callimus angulatus (27), Cerambyx miles (9), Chlorophorus trifasciatus (3), Clytus rhamni (12), Cortodera humeralis (14), Glaphyra marmottani (8), Gracilia minuta (19), Iberodorcadion fuliginator (136), Leioderes kollari (29), Moneilema armatum (1), Nathrius brevipennis (15), Necydalis major (23), Oberea erythrocephala (127), O. pedemontana (3), Opsilia coerulescens (395), Osphranteria coerulescens (1), Phytoecia affinis (146), P. caerulea (1), P. cylindrica (493), P. icterica (93), P. nigricornis (74), P. pustulata (59), P. rufipes (1), P. virgula (18), Poecilium glabratum (28), P. lividum (1), P. pusillum (7), Pogonocherus caroli (6), Purpuricenus globulicollis (14), P. temminckii (2), Ropalopus ungaricus (5), R. varini (1), Rusticoclytus pantherinus (5), Saperda punctata (6), S. similis (14), Stenopterus ater (1), Stenurella sennii (3), Stictoleptura erythroptera (11), Trichoferus campestris (4), T. pallidus (2), Xylotrechus arvicola (5)

			(66), R. femoratus (70), Rosalia alpina (644), Rusticoclytus rusticus (172), Rutpela maculata (5010), Saperda carcharias (264), S. octopunctata (163), S. perforata (18), S. populnea (369), S. scalaris (455), Saphanus piceus (41), Semanotus undatus (37), Spondylis buprestoides (371), Stenocorus meridianus (893), Stenopterus rufus (1043), Stenostola dubia (363), S. ferrea (49), Stenurella bifasciata (1140), S. melanura (4336), S. nigra (442), Stictoleptura cordigera (37), S. fulva (434), S. hybrida (428), S. maculicornis aggr. (977), S. rubra (2900), S. scutellata (59), Strangalia attenuata (178), Tetropium castaneum (712), T. fuscum (157), T. gabrieli (145), Tetrops praeustus (496), T. starkii (60), Tragosoma depsarium (23), Trichoferus holosericeus (26), Xylotrechus antilope (179), X. stebbingi (54)	
Cleroidea	Cleridae	Cleroidea	Clerus mutillarius (168), Korynetes caeruleus (57), K. ruficornis (100), Opilo mollis (210), Thanasimus femoralis (55), T. formicarius (794), Tilloidea unifasciata (96), Tillus elongatus (149)	Allonyx quadrimaculatus (23), Denops albofasciatus (1), Dermestoides sanguinicollis (6), Necrobia ruficollis (23), N. rufipes (33), N. violacea (134), Opilo domesticus (7), O. pallidus (11), Tarsostenus univittatus (6), Trichodes alvearius (813), T. apiarius (1035)
Cleroidea	Dasytidae	Cleroidea	Aplocnemus alpestris (72), A. impressus (120), A. nigricornis (44), A. virens (27), Dasytes aeratus (308), D. caeruleus (230), D. fusculus (26), D. niger (216), D. obscurus (137), D. plumbeus (669), D. subalpinus (21), D. virens (252)	Aplocnemus chalconatus (14), A. integer (4), A. tarsalis (8), Danacea ambigua (24), D. denticollis (26), D. montivaga (1), D. nigritarsis (172), D. pallipes (192), Dasytes alpigradus (97), D. gonocerus (71), D. lombardus (4), D. nigrocyaneus (3), D. pauperculus (3), D. subaeneus (3), D. thoracicus (6), Divales bipustulatus (34), Dolichosoma lineare (42), Enicopus pilosus (5), Psilothrix viridicoerulea (28), Trichoceble memnonia (6)

Cleroidea	Malachiidae	Cleroidea	Anthocomus equestris (167), A. fasciatus (183), Hypebaeus flavipes (47), Sphinginus lobatus (34)	Anthocomus humeralis (1), A. rufus (120), Attalus alpinus (2), A. analis (80), A. minimus (24), Axinotarsus marginalis (168), A. pulicarius (64), A. ruficollis (106), Cerapheles terminatus (29), Charopus concolor (54), C. docilis (2), C. flavipes (19), C. madidus (12), C. pallipes (6), Clanoptilus affinis (3), C. elegans (282), C. emarginatus (133), C. geniculatus (3), C. marginellus (66), Cordylepherus viridis (146), Cyrtosus ovalis (13), Ebaeus battonii (8), E. collaris (1), E. flavicornis (1), E. gibbus (6), E. pedicularius (7), E. thoracicus (56), Hypebaeus albifrons (39), Malachius aeneus (209), M. australis (70), M. bipustulatus (449), M. rubidus (59), M. scutellaris (9), Micrinus heteromorphus (11), M. inornatus (73), Nepachys cardiacae (8), Sphinginus coarctatus (11), Troglops albicans (68), T. silo (30)
Cleroidea	Phloiophilidae	Cleroidea	–	Phloiophilus edwardsii (1)
Cleroidea	Trogossitidae	Cleroidea	Nemozoma elongatum (126), Peltis ferruginea (100), Temnoscheila caerulea (56), Thymalus limbatus (78)	Nemozoma caucasicum (26), Peltis grossa (7), Tenebroides mauritanicus (43)
Cucujoidea	Alexiidae	Cucujoidea	–	Sphaerosoma globosum (66), S. pilosum (265), S. quercus (146), S. seidlitzi (1)
Cucujoidea	Biphyllidae	Cucujoidea	Diplocoelus fagi (66)	Biphyllus lunatus (2)
Cucujoidea	Bothrideridae	Cucujoidea	Oxylaemus cylindricus (28), Teredus cylindricus (33)	Anommatus besucheti (12), A. dentatus (6), A. diecki (52), A. duodecimstriatus (125), A. reitteri (6), A. scherleri (18), A. ticinensis (2), Bothrideres bipunctatus (3), Oxylaemus variolosus (13), Teredus opacus (1)
Cucujoidea	Cerylonidae	Cucujoidea	Cerylon deplanatum (20), C. fagi (186), C. ferrugineum (244), C. histeroides (355)	Cerylon impressum (1), Philothermus evanescens (1)
Cucujoidea	Cucujidae	Cucujoidea	Pediacus depressus (39), P. dermestoides (94)	–

Cucujoidea	Endomychidae	Cucujoidea	Endomychus coccineus (180), Mycetina cruciata (156), Symbiotes gibberosus (77)	Holoparamecus caularum (1), H. depressus (2), H. ragusae (1), H. singularis (10), Lycoperdina bovistae (62), L. succincta (5), Mycetaea subterranea (137), Symbiotes armatus (5), S. latus (7)
Cucujoidea	Erotylidae	Cucujoidea	Dacne bipustulata (288), Triplax russica (102), Tritoma bipustulata (407)	Cryptophilus integer s.l. (3), C. obliteratus (3), C. propinquus (3), Dacne rufifrons (26), Triplax aenea (8), T. lacordairii (17), T. lepida (38), T. melanocephala (2), T. rufipes (61), T. scutellaris (3), Tritoma subbasalis (1)
Cucujoidea	Laemophloeidae	Cucujoidea	Cryptolestes duplicatus (83), Laemophloeus monilis (57), Leptophloeus alternans (29), L. clematidis (44), L. juniperi (20), Notolaemus unifasciatus (20), Placonotus testaceus (118)	Cryptolestes abietis (2), C. corticinus (4), C. ferrugineus (112), C. pusillus (7), C. spartii (25), Laemophloeus kraussi (4), L. muticus (3), L. nigricollis (2), Lathropus sepicola (11), Notolaemus castaneus (11)
Cucujoidea	Monotomidae	Cucujoidea	Rhizophagus bipustulatus (281), R. cribratus (36), R. depressus (90), R. dispar (280), R. ferrugineus (136), R. nitidulus (48), R. parallelocollis (43), R. perforatus (193), R. picipes (69)	Monotoma angusticollis (22), M. bicolor (43), M. brevicollis (75), M. conicicollis (34), M. longicollis (74), M. picipes (314), M. quadricollis (2), M. quadrifoveolata (1), M. spinicollis (14), M. testacea (2), Rhizophagus aeneus (12), R. fenestralis (3), R. grandis (2)
Cucujoidea	Phloeostichidae	Cucujoidea	–	Phloeostichus denticollis (15)
Cucujoidea	Silvanidae	Cucujoidea	Silvanoprus fagi (99), Silvanus bidentatus (117), S. unidentatus (238), Uleiota planatus (474)	Ahasverus advena (104), Airaphilus elongatus (10), A. perangustus (1), Cryptamorpha desjardinsii (1), Dendrophagus crenatus (11), Oryzaephilus mercator (26), O. surinamensis (47), Psammoecus bipunctatus (150)
Cucujoidea	Sphindidae	Cucujoidea	Aspidiphorus lareyiniei (28), A. orbiculatus (188), Sphindus dubius (89)	–
Curculionoidea	Anthribidae	Rest	Anthribus nebulosus (173), Dissoleucas niveirostris (177), Platyrhinus resinosus (213), Platystomos albinus (291), Pseudeuparius sepicola (91), Rhaphitropis marchica (42), Tropideres albirostris (159)	Allandrus undulatus (4), Anthribus fasciatus (13), Araecerus fasciculatus (5), Bruchela rufipes (17), B. suturalis (8), Choragus horni (2), C. sheppardi (1), Gonotropis gibbosa (6), Noxius curtirostris (4), Opanthribus tessellatus (6), Phaeochrotes pudens (4), Pseudochoragus piceus (1), Rhaphitropis oxyacanthae (3), Ulorhinus bilineatus (7)

Curculionoidea	Curculionidae	Curculionidae	Anisandrus dispar (853), Carphoborus minimus (40), Cryphalus asperatus (432), C. intermedius (39), C. piceae (306), C. saltuarius (38), Crypturgus cinereus (106), C. hispidulus (151), C. pusillus (349), Dendroctonus micans (95), Dryocoetes alni (48), D. autographus (922), D. hectographus (114), D. villosus (144), Ernoporicus fagi (196), Ernoporus tiliae (41), Hylastes angustatus (109), H. ater (430), H. attenuatus (298), H. brunneus (122), H. cunicularius (1013), H. linearis (63), H. opacus (34), Hylastinus fankhauseri (38), H. obscurus (141), Hylesinus crenatus (69), H. toranio (165), H. varius (702), H. wachtli (94), Hylurgops glabratus (70), H. palliatus (594), Ips acuminatus (101), I. amitinus (157), I. cembrae (204), I. sexdentatus (85), I. typographus (805), Kissophagus vicinus (43), Lymantor coryli (39), Orthotomicus laricis (170), O. longicollis (45), Phloeosinus aubei (23), P. thujae (54), Phloeotribus rhododactylus (34), P. spinulosus (150), Pityogenes bidentatus (58), P. chalcographus (1279), P. conjunctus (312), P. quadridens (46), P. trepanatus (24), Pityokteines curvidens (115), P. spinidens (46), P. vorontzowi (25), Pityophthorus glabratus (40), P. henscheli (63), P. knoteki (32), P. lichtensteinii (28), P. pityographus (595), P. pubescens (74), Platypus cylindrus (135), Polygraphus grandiclava (91), P. poligraphus (306), Scolytus intricatus (307), S. laevis (25), S. mali (55), S. multistriatus (78), S. rugulosus (144), S. scolytus (32), Taphrorychus bicolor (753), T. villifrons (35), Tomicus minor (77), T. piniperda (186), Trypodendron domesticum (243), T. lineatum (629), T. signatum (194), Xyleborinus saxesenii (895), Xyleborus dryographus (237), X. monographus (223), Xylocleptes bispinus (222), Xylosandrus germanus (1576)	Coccotrypes dactyliperda (1), Crypturgus subcribrosus (4), Cyclorhipidion bodoanum (22), Dryocoetes himalayensis (4), Ernoporicus caucasicus (36), Gnathotrichus materiarius (17), Heteroborips cryptographus (12), Hylastes gergeri (1), Hylurgus ligniperda (1), Hypoborus ficus (17), Ips duplicatus (13), Kissophagus novaki (7), Orthotomicus erosus (3), O. proximus (16), O. suturalis (15), Pagiocerus frontalis (1), Phloeotribus scarabaeoides (24), Pityogenes bistridentatus (7), Pityophthorus buyssoni (2), P. carniolicus (1), P. exsculptus (9), Polygraphus subopacus (6), Pteleobius kraatzii (15), P. vittatus (15), Scolytus carpini (35), S. ensifer (1), S. kirschii (1), S. pygmaeus (30), S. ratzeburgii (13), Taphrorychus siculus (1), Thamnurgus kaltenbachii (2), Trypodendron laeve (1), Trypophloeus binodulus (18), Xyleborinus attenuatus (27), Xyleborus eurygraphus (2), X. pfeilii (1), Xylechinus pilosus (81), Xylosandrus crassiusculus (2)
Derodontoidea	Derodontidae	Rest	–	Derodontus macularis (2), Laricobius erichsonii (36)

Elateroidea	Cantharidae	Cantharidae	Malthinus balteatus (37), M. biguttatus (58), M. facialis (40), M. fasciatus (42), M. flaveolus (342), M. frontalis (20), M. glabellus (39), M. seriepunctatus (145), Malthodes aemulus (21), M. alpicola (35), M. brevicollis (30), M. dispar (150), M. flavoguttatus (83), M. fuscus (117), M. guttifer (42), M. hexacanthus (232), M. marginatus (261), M. maurus (216), M. minimus (51), M. misellus (25), M. mysticus (96), M. penninus (42), M. spathifer (50), M. spretus (77), M. trifurcatus (171)	Ancistronycha abdominalis (239), A. erichsonii (254), A. tigurina (348), Cantharis annularis (123), C. cryptica (134), C. decipiens (339), C. fibulata (52), C. figurata (185), C. flavilabris (57), C. fusca (306), C. gemina (69), C. lateralis (150), C. livida (866), C. montana (47), C. nigra (109), C. nigricans (720), C. obscura (101), C. pagana (7), C. pallida (186), C. paludosa (122), C. paradoxa (2), C. pellucida (481), C. pulicaria (24), C. quadripunctata (1), C. rufa (457), C. rustica (641), C. terminata (131), C. tristis (278), Cratosilis denticollis (72), C. distinguenda (53), C. laeta (184), Malthinus bilineatus (8), M. rubricollis (3), M. sordidus (10), Malthodes bertolinii (43), M. caudatus (9), M. crassicornis (1), M. cyphonurus (30), M. debilis (3), M. europaeus (13), M. facetus (12), M. fibulatus (3), M. holdhausi (4), M. icaricus (1), M. kahleni (2), M. lobatus (19), M. pumilus (19), M. setifer (18), M. siculus (16), M. stolzi (1), M. umbrosus (4), M. vincens (9), Metacantharis clypeata (180), M. discoidea (142), Podabrus alpinus (505), Podistra prolixa (143), P. rufotestacea (198), P. schoenherri (139), Rhagonycha atra (92), R. elongata (5), R. fugax (25), R. fulva (1014), R. fuscitibia (129), R. gallica (45), R. lignosa (778), R. lutea (380), R. maculicollis (45), R. nigriceps (104), R. nigripes (168), R. nigriventris (326), R. pedemontana (1), R. testacea (150), R. translucida (493), Silis ruficollis (48)
Elateroidea	Cerophytidae	Rest	–	Cerophytum elateroides (15)

Elateroidea	Elateridae	Elateridae	Ampedus balteatus (190), A. cinnabarinus (32), A. elongatulus (216), A. erythrogonus (172), A. nigerrimus (62), A. nigrinus aggr. (110), A. pomonae (93), A. pomorum aggr. (687), A. praeustus (122), A. quercicola (421), A. rufipennis (29), A. sanguineus (263), A. sanguinolentus (196), A. scrofa (264), A. sinuatus (99), Brachygonus megerlei (103), Calambus bipustulatus (117), Cardiophorus gramineus (75), C. nigerrimus (178), C. ruficollis (103), C. rufipes (307), Danosoma fasciata (94), Denticollis linearis (791), D. rubens (124), Diacanthous undulatus (61), Drapetes mordelloides (55), Hypoganus inunctus (91), Ischnodes sanguinicollis (46), Melanotus castanipes (567), M. crassicollis (248), M. villosus (870), Porthmidius austriacus (27), Procraerus tibialis (29), Stenagostus rhombeus (218), S. rufus (28)	Actenicerus sjaelandicus (275), Adrastus axillaris (57), A. lacertosus (74), A. limbatus (198), A. montanus (41), A. pallens (274), A. rachifer (185), A. sekerae (16), Agriotes acuminatus (325), A. brevis (12), A. gallicus (239), A. lineatus (207), A. litigiosus (90), A. obscurus (469), A. pallidulus (65), A. pilosellus (359), A. proximus (8), A. sputator (472), A. ustulatus (362), Agrypnus murinus (1685), Ampedus (4), A. brunnicornis (18), A. cardinalis (6), A. elegantulus (5), A. melanurus (12), A. nigroflavus (13), A. tristis (3), Anostirus castaneus (32), A. gracilicollis (95), A. purpureus (416), A. sulphuripennis (59), Aplotarsus angustulus (3), A. incanus (167), Athous bicolor (262), A. campyloides (6), A. emaciatus (142), A. flavipennis (46), A. haemorrhoidalis (2027), A. subfuscus (1293), A. tomentosus (5), A. vittatus (1130), A. zebei (201), Berninelsonius hyperboreus (14), Betarmon bisbimaculatus (72), Brachygonus bouyoni (3), B. ruficeps (29), Cardiophorus asellus (9), C. atramentarius (42), C. collaris (1), C. ebeninus (71), C. erichsoni (117), Cidnopus aeruginosus (131), C. pilosus (683), Crepidophorus mutilatus (7), Ctenicera cuprea (530), C. doderoi (13), C. heyeri (1), C. pectinicornis (332), C. virens (287), Dalopius marginatus (1245), Danosoma conspersa (7), Dicronychus cinereus (239), D. equiseti (14), D. equisetoides (2), Drasterius bimaculatus (56), Ectinus aterrimus (28), Elater ferrugineus (40), Fleutiauxellus maritimus (45), Hemicrepidius hirtus (562), H. niger (597), Hypnoidus consobrinus (20), H. riparius (117), H. rivularius (68), Idolus picipennis (225), Lacon lepidopterus (47), L. punctatus (7), L. querceus (7), Limoniscus violaceus (2), Limonius minutus aggr. (449), Liotrichus affinis (64), Megapenthes lugens (10), Melanotus brunnipes (3), M. dichrous (1), M. punctolineatus (267), M. tenebrosus (53), Negastrius pulchellus (10), N. sabulicola (5), Nothodes parvulus (693), Oedostethus tenuicornis (3), Paracardiophorus musculus (53), Paraphotistus impressus (70), P. nigricornis (17), Pheletes aeneoniger (124), P. quercus (323), Podeonius acuticornis (15), Prosternon tessellatum (500), Pseudanostirus globicollis (1), Quasimus minutissimus (55), Reitterelater dubius (2), Selatosomus aeneus (631), S. confluens (213), S. cruciatus (3), S. latus (110), S. melancholicus (36), Seri-

				cus brunneus aggr. (127), S. subaeneus (30), Synaptus filiformis (190), Zorochros boubersi (4), Z. demustoides (219), Z. dufouri (135), Z. flavipes (87), Z. meridionalis (179), Z. quadriguttatus (34), Z. trigonochirus (1)
Elateroidea	Eucnemidae	Rest	Dromaeolus barnabita (53), Eucnemis capucina (78), Hylis cariniceps (63), H. foveicollis (72), Melasis buprestoides (184), Microrhagus emyi (50), M. lepidus (83), M. pygmaeus (52)	Hylis olexai (70), H. procerulus (8), H. simonae (8), Isorhipis melasoides (37), Microrhagus pyrenaeus (2), Rhacopus sahlbergi (20), Xylophilus corticalis (1)
Elateroidea	Lampyridae	Rest	–	Lamprohiza splendidula (357), Lampyris noctiluca (3234), Luciola italica (173), L. lusitanica (4), Phosphaenus hemipterus (411)
Elateroidea	Lycidae	Rest	Dictyoptera aurora (93), Erotides cosnardi (49), Lopheros rubens (92), Lygistopterus sanguineus (130), Platycis minutus (81), Pyropterus nigroruber (47)	–
Elateroidea	Omalisidae	Rest	–	Omalisus fontisbellaquei (209), O. victoris (1)

Hydrophiloidea	Histeridae	Rest	Abraeus granulum (35), A. perpusillus (125), Dendrophilus punctatus (90), Eurosomides minor (31), Hololepta plana (84), Paromalus flavicornis (240), P. parallelepipedus (99), Platysoma compressum (44), P. elongatum (50), Plegaderus caesus (54)	Acritus homoeopathicus (11), A. komai (8), A. minutus (1), A. nigricornis (63), Atholus bimaculatus (69), A. corvinus (46), A. duodecimstriatus (176), Carcinops pumilio (119), Chaetabraeus globulus (2), Chalcionellus decemstriatus (4), Dendrophilus pygmaeus (53), Euspilotus ornatus (1), Gnathoncus buyssoni (159), G. communis (46), G. nannetensis (15), G. nidorum (6), G. rotundatus (57), Haeterius ferrugineus (19), Hister bissexstriatus (31), H. funestus (29), H. helluo (21), H. illigeri (20), H. quadrimaculatus (208), H. quadrinotatus (19), H. unicolor (386), Hypocaccus rugiceps (37), H. rugifrons (13), H. specularis (9), Margarinotus brunneus (96), M. carbonarius (96), M. ignobilis (41), M. marginatus (2), M. merdarius (86), M. neglectus (12), M. obscurus (103), M. punctiventer (16), M. purpurascens (138), M. ruficornis (3), M. striola (73), M. terricola (3), M. ventralis (112), Myrmetes paykulli (19), Onthophilus affinis (8), O. striatus (68), Pachylister inaequalis (16), Platysoma lineare (15), Plegaderus discisus (8), P. dissectus (10), P. saucius (23), P. vulneratus (16), Saprinus acuminatus (1), S. aegialius (2), S. aeneus (10), S. caerulescens (1), S. detersus (1), S. immundus (4), S. maculatus (1), S. planiusculus (25), S. politus (1), S. rugifer (2), S. semistriatus (93), S. subnitescens (51), S. tenuistrius (3), S. virescens (2), Teretrius fabricii (3)
Hydrophiloidea	Sphaeritidae	Rest	Sphaerites glabratus (41)	–
Lymexyloidea	Lymexylidae	Rest	Elateroides dermestoides (319), Lymexylon navale (55)	–
Scarabaeoidea	Lucanidae	Scarabaeoidea	Ceruchus chrysomelinus (62), Dorcus parallelipipedus (1870), Lucanus cervus (3406), Platycerus caprea (315), P. caraboides (457), Sinodendron cylindricum (261)	Aesalus scarabaeoides (1)
Scarabaeoidea	Scarabaeidae	Scarabaeoidea	Cetonia aurata (4664), Gnorimus nobilis (403), G. variabilis (95), Osmoderma eremita (63), Protaetia angustata (35), P. cuprea s.l. (1470), P. fieberi (42), P. marmorata (209), P. speciosissima (181), Trichius fasciatus (2743), T. gallicus (44), T. sexualis (80), Valgus hemipterus (938)	Oxythyrea funesta (1251), Protaetia affinis (18), P. morio (75), Tropinota hirta (659), T. squalida (1)

Scarabae-oidea	Trogidae	Scarabae-oidea	–	Trox hispidus (1), T. niger (52), T. perlatus (1), T. perrisii (8), T. sabulosus (36), T. scaber (391)
Tenebrio-noidea	Aderidae	Tenebrio-noidea	Aderus populneus (74), Anidorus nigrinus (64), Euglenes pygmaeus (34)	Cobososia pruinosa (10), Euglenes oculatus (66), Gompelia flaveola (1), Phytobaenus amabilis (1)
Tenebrionoidea	Ciidae	Tenebrionoidea	Cis bidentatus (22), C. boleti (328), C. comptus (35), C. dentatus (44), C. festivus (46), C. fusciclavis (51), C. glabratus (75), C. micans (191), C. punctulatus (26), C. rugulosus (31), C. villosulus (86), Ennearthron cornutum (169), Octotemnus glabriculus (261), O. mandibularis (17), Orthocis alni (75), Strigocis bicornis (28), Sulcacis fronticornis (67), S. nitidus (218)	Cis castaneus (86), C. chinensis (3), C. fagi (11), C. festivoides (7), C. fissicornis (8), C. hanseni (1), C. laminatus (1), C. lineatocribratus (2), C. pygmaeus (8), C. quadridens (30), C. striatulus (3), C. submicans (12), C. vestitus (15), Ennearthron pruinosulum (4), Orthocis coluber (1), O. linearis (3), O. lucasi (10), Ropalodontus novorossicus (17), R. perforatus (3), Sulcacis bidentulus (13), Wagaicis wagae (1), Xylographus bostrichoides (4)
Tenebrionoidea	Melandryidae	Tenebrionoidea	Conopalpus brevicollis (66), C. testaceus (34), Melandrya caraboides (183), Orchesia micans (52), O. minor (57), O. undulata (112), Osphya aeneipennis (27), Phloiotrya rufipes (31), P. tenuis (22), Serropalpus barbatus (113)	Abdera flexuosa (5), A. quadrifasciata (22), Anisoxya fuscula (22), Dircaea australis (1), Dolotarsus lividus (28), Hypulus quercinus (11), Marolia leseigneuri (8), M. variegata (2), Melandrya barbata (3), Orchesia blandula (3), O. fasciata (1), Osphya bipunctata (2), Rushia parreyssii (29), Wanachia triguttata (13), Xylita laevigata (9), Zilora obscura (2)
Tenebrio-noidea	Mycetopha-gidae	Tenebrio-noidea	Litargus connexus (365), Mycetophagus atomarius (125), M. multipunctatus (31), M. quadriguttatus (87), M. quadripustulatus (305), Triphyllus bicolor (32)	Berginus tamarisci (30), Litargus balteatus (12), Mycetophagus ater (2), M. decempunctatus (1), M. fulvicollis (1), M. piceus (44), M. populi (14), Typhaea stercorea (310)
Tenebrionoidea	Oedemeridae	Tenebrionoidea	Anogcodes fulvicollis (142), A. melanurus (91), A. rufiventris (827), Calopus serraticornis (43), Chrysanthia geniculata (88), C. viridissima (376), Ischnomera caerulea (65), I. cyanea (55), I. sanguinicollis (16), Nacerdes carniolica (697), Oedemera femoralis (304)	Anogcodes ruficollis (4), A. ustulatus (4), Ischnomera cinerascens (15), Nacerdes gracilis (8), Oedemera croceicollis (10), O. femorata (655), O. flavipes (308), O. lateralis (20), O. lurida (716), O. monticola (74), O. nobilis (667), O. podagrariae (459), O. pthysica (233), O. subrobusta (10), O. tristis (205), O. virescens (558)
Tenebrio-noidea	Prostomi-dae	Tenebrio-noidea	Prostomis mandibularis (46)	–
Tenebrio-noidea	Pyrochroi-dae	Tenebrio-noidea	Pyrochroa coccinea (470), P. serraticornis (673), Schizotus pectinicornis (184)	–

Tenebrio-noidea	Pythidae	Tenebrio-noidea	Pytho depressus (49)	–
Tenebrio-noidea	Salpingidae	Tenebrio-noidea	Lissodema cursor (35), L. denticollis (27), Salpingus planirostris (333), S. ruficollis (170), Sphaeriestes castaneus (41), Vincenzellus ruficollis (188)	Aglenus brunneus (11), Cariderus aeneus (3), Colposis mutilatus (6), Rabocerus foveolatus (11), R. gabrieli (6), Sphaeriestes aeratus (6), S. stockmanni (6)
Tenebrionoidea	Scraptiidae	Tenebrionoidea	Anaspis brunnipes (57), A. costai (111), A. fasciata (68), A. flava (103), A. frontalis (497), A. kiesenwetteri (34), A. latiuscula (94), A. lurida (141), A. maculata (340), A. pulicaria (138), A. quadrimaculata (43), A. regimbarti (25), A. ruficollis (85), A. rufilabris (215), A. thoracica (95), A. varians (93), Scraptia dubia (22), S. ferruginea (32)	Anaspis bohemica (3), A. garneysi (2), A. kriegeri (8), A. melanostoma (10), A. nigripes (11), A. palpalis (4), A. viennensis (2), Cyrtanaspis phalerata (22), Pentaria badia (12), Scraptia fuscula (37), S. testacea (7)
Tenebrionoidea	Tenebrionidae	Tenebrionoidea	Accanthopus velikensis (70), Allecula morio (57), Corticeus bicolor (58), C. linearis (48), C. longulus (54), C. unicolor (156), Diaclina fagi (23), Diaperis boleti (254), Eledona agricola (78), Gonodera luperus (287), Helops caeruleus (129), Hymenalia rufipes (265), Mycetochara humeralis (25), M. maura (140), Nalassus dryadophilus (30), N. ecoffeti (71), Palorus depressus (63), Platydema violacea (50), Prionychus ater (86), P. melanarius (148), Pseudocistela ceramboides (234), Scaphidema metallica (73), Stenomax aeneus (234), Tenebrio opacus (25)	Allecula rhenana (4), Alphitobius diaperinus (132), Alphitophagus bifasciatus (32), Asida sabulosa (68), Bius thoracicus (2), Blaps lethifera (40), B. mortisaga (2), B. mucronata (48), Bolitophagus reticulatus (12), Corticeus bicoloroides (2), C. fasciatus (11), C. fraxini (12), Cteniopus sulphureus (435), Dendarus coarcticollis (105), Gnatocerus cornutus (5), Gonocephalum granulatum (1), G. pygmaeum (5), Gonodera metallica (3), Hymenophorus doublieri (5), Isomira costessii (2), I. hypocrita (79), I. marcida (26), I. testacea (1), I. thoracica (437), Lagria atripes (50), L. hirta (753), L. rugosula (65), Lamprocrypticus alpinus (28), Latheticus oryzae (1), Melanimon tibialis (23), Menephilus cylindricus (15), Mycetochara quadrimaculata (6), Myrmechixenus picinus (1), M. subterraneus (29), M. vaporariorum (12), Nalassus convexus (191), N. dermestoides (2), N. laevioctostriatus (1), Neatus picipes (5), Omophlus lepturoides (43), O. pubescens (60), O. rugosicollis (3), Opatrum riparium (11), O. sabulosum (276), Pentaphyllus testaceus (24), Tenebrio molitor (285), T. obscurus (16), Tribolium castaneum (30), T. confusum (11), T. destructor (36), Uloma culinaris (47), U. rufa (12), Zophobas atratus (1)
Tenebrio-noidea	Tetratomi-dae	Tenebrio-noidea	Hallomenus binotatus (106)	Eustrophus dermestoides (5), Hallomenus axillaris (7), Mycetoma suturale (7), Tetratoma ancora (5), T. desmarestii (2), T. fungorum (18)

Tenebrionoidea	Zopheridae	Tenebrionoidea	Aulonium trisulcum (39), Bitoma crenata (336), Colobicus hirtus (54), Colydium elongatum (73), Coxelus pictus (324), Diodesma subterranea (63), Pycnomerus terebrans (31), Synchita humeralis (68), S. undata (31), S. variegata (40)	Langelandia anophthalma (121), L. excavata (1), Orthocerus clavicornis (22), Rhopalocerus rondanii (20), Synchita mediolanensis (11), S. separanda (10)

Supplementary Table 2 | Overview of butterfly species for which records were considered in the analyses. Here, we refer to butterflies as Papilionoidea as well as Zygaenidae moths. Species are grouped by family. For species in the column 'Analysed', occupancy-detection models were fitted to determine trajectories. Species in the column 'Not Analysed' were part of the analysed dataset that went into occupancy-detection models, but no models were fitted for these species. Either due to low sample sizes or due to imprecise taxonomic identification (aggregates). Numbers in brackets give the number of records that were included per species in the final dataset.

Family	Analysed	Not Analysed
Hesperiidae	Carcharodus alceae (2939), Carterocephalus palaemon (3628), Erynnis tages (8883), Hesperia comma (5532), Heteropterus morpheus (357), Muschampia floccifera (380), M. lavatherae (443), Ochlodes sylvanus (16663), Pyrgus accreta (305), P. alveus (1880), P. andromedae (381), P. armoricanus (1156), P. cacaliae (673), P. carlinae (506), P. carthami (939), P. cirsii (60), P. malvae (3741), P. malvoides (2196), P. onopordi (131), P. serratulae (1445), P. warrenensis (86), Spialia sertorius (5157), Thymelicus acteon (422), T. lineola (5875), T. sylvestris (8718)	Muschampia baeticus (2), Pyrgus alveus aggr. (288), P. malvae aggr. (18)
Lycaenidae	Agriades glandon (1265), A. optilete (873), A. orbitulus (1168), Aricia agestis (4006), A. artaxerxes (3212), A. eumedon (2365), A. nicias (333), Callophrys rubi (5191), Celastrina argiolus (6858), Cupido alcetas (3395), C. argiades (5332), C. minimus (9951), C. osiris (436), Cyaniris semiargus (16936), Favonius quercus (1263), Glaucopsyche alexis (1285), Iolana iolas (417), Kretania trappi (345), Lampides boeticus (326), Lycaena alciphron (1060), L. dispar (445), L. helle (816), L. hippothoe (4249), L. phlaeas (5744), L. tityrus (9882), L. virgaureae (4161), Lysandra bellargus (11991), L. coridon (9597), Phengaris alcon (1318), P. arion (4181), P. nausithous (2051), P. teleius (1273), Plebejus argus (1986), P. argyrognomon (632), P. idas (3021), Polyommatus amandus (584), P. damon (1867), P. daphnis (387), P. dorylas (2358), P. eros (1352), P. escheri (541), P. icarus (32004), P. thersites (1797), Pseudophilotes baton (828), Satyrium acaciae (246), S. ilicis (465), S. pruni (640), S. spini (914), S. w-album (1452), Scolitantides orion (1074), Thecla betulae (2155)	Aricia agestis aggr. (1489), Cacyreus marshalli (2), Leptotes pirithous (6)

Nymphalidae	Aglais io (17276), A. urticae (35313), Apatura ilia (975), A. iris (2536), Aphantopus hyperantus (17279), Araschnia levana (7053), Argynnis paphia (17145), Boloria aquilonaris (581), B. dia (5990), B. euphrosyne (7323), B. napaea (1238), B. pales (2627), B. selene (2635), B. thore (481), B. titania (4567), Brenthis daphne (2825), B. ino (5477), Brintesia circe (4046), Chazara briseis (120), Coenonympha arcania (2635), C. darwiniana (1047), C. gardetta (3989), C. glycerion (847), C. oedippus (42), C. pamphilus (28759), C. tullia (374), Erebia aethiops (6737), E. alberganus (2453), E. arvernensis (395), E. bubastis (61), E. christi (136), E. epiphron (1789), E. eriphyle (394), E. euryale (5045), E. flavofasciata (122), E. gorge (1321), E. ligea (6349), E. manto (1671), E. medusa (2984), E. melampus (4130), E. meolans (1395), E. mnestra (916), E. montanus (1951), E. nivalis (52), E. oeme (2008), E. pandrose (1550), E. pharte (1720), E. pluto (717), E. pronoe (1280), E. styx (131), E. sudetica (92), E. triarius (681), E. tyndarus (4041), Euphydryas aurinia (2968), E. cynthia (831), E. intermedia (256), Fabriciana adippe (5923), F. niobe (3947), Hipparchia fagi (439), H. genava (1023), H. semele (1524), H. statilinus (468), Hyponephele lycaon (1278), Issoria lathonia (8910), Lasiommata maera (8726), L. megera (15148), L. petropolitana (2111), Libythea celtis (97), Limenitis camilla (7636), L. populi (378), L. reducta (710), Lopinga achine (1120), Maniola jurtina (30800), Melanargia galathea (24005), Melitaea asteria (102), M. athalia aggr. (10766), M. aurelia (496), M. cinxia (2329), M. deione (281), M. diamina (6628), M. didyma (3653), M. parthenoides (2697), M. phoebe (2250), M. varia (778), Minois dryas (2599), Neptis rivularis (235), Nymphalis antiopa (3940), N. polychloros (3134), Oeneis glacialis (870), Pararge aegeria (19433), Polygonia c-album (13860), Pyronia tithonus (1532), Satyrus ferula (2035), Speyeria aglaja (8526), Vanessa atalanta (31460), V. cardui (20273)	Arethusana arethusa (1), Argynnis pandora (369), Boloria pales aggr. (648), Charaxes jasius (1), Coenonympha gardetta aggr. (615), C. hero (5), Erebia cassioides x tyndarus (2), Hipparchia fagi aggr. (23), Melitaea britomartis (48), Pyronia cecilia (1)
Papilio-nidae	Iphiclides podalirius (5051), Papilio machaon (21023), Parnassius apollo (6812), P. mnemosyne (1161), P. phoebus (1434)	Zerynthia polyxena (1)
Pieridae	Anthocharis cardamines (18999), Aporia crataegi (7658), Colias croceus (11187), C. hyale aggr. (15430), C. palaeno (2021), C. phicomone (3572), Euchloe simplonia (417), Gonepteryx rhamni (25114), Leptidea sinapis aggr. (16627), Pieris brassicae (14340), P. bryoniae (3045), P. mannii (2854), P. napi (24669), P. rapae (22712), Pontia callidice (1001), P. edusa (1005)	Anthocharis euphenoides (1), Gonepteryx cleopatra (1), Pieris napi aggr. (6325), P. rapae aggr. (7149)

Zygaenidae	Adscita alpina (524), A. dujardini (83), A. geryon (1129), A. mannii (37), A. statices (1293), Jordanita globulariae (201), J. notata (149), J. subsolana (70), Rhagades pruni (198), Zygaena carniolica (1379), Z. ephialtes (604), Z. exulans (1522), Z. fausta (499), Z. filipendulae (9092), Z. lonicerae (2952), Z. loti (3239), Z. minos (204), Z. osterodensis (297), Z. purpuralis (2617), Z. romeo (305), Z. transalpina (3959), Z. trifolii (172), Z. viciae (1915)	Adscita statices x alpina (38), Aglaope infausta (1), Jordanita chloros (1), J. notata/globulariae aggr. (8)

Supplementary Table 3 | Overview of variables of environmental change. For each variable, the type (continuous or factor), the range (including unit), the source of the raw data, the years for which data were available and used in the analyses, how data were edited and the calculation of the variables are given.

Variable	Type	Range	Raw data source	Years used	Data edits	Calculation
Temperature absolute	cont.	-0.312–2.04 °C	Reconstructed mean annual temperature from MeteoSwiss (https://www.meteoswiss.admin.ch) at a 1.25-degree minute grid (~2.3 km × 1.6 km)	1901–2021	Averaged per biozone, anomaly per biozone relative to the mean of 1901–2000	Linear regression intercept per eight-year interval (year centred)
Temperature change	cont.	-0.199–0.229 °C/yr				Linear regression slope per eight-year interval
Human population density change	cont.	-0.440–5.39 ind./km^2/yr	Data on inhabitants per canton[1] from cenuses[2]	1930, 1941, 1950, 1960, 1970–2021	Attributed cantonal data to biozones while accounting for location of buildings in the 1985 land-use statistics (Bundesamt für Statistik, 2015). Interpolated missing years. Data per biozone relative to total biozone area.	Linear regression slope per eight-year interval
Mechanisation absolute	cont.	0.00462–7.02 tractors/km^2	Data on the number of tractors per canton[1] from agricultural cenuses[3]	1929, 1939, 1946, 1950, 1955, 1960, 1965, 1969, 1975, 1980, 1985, 1990–2021	Attributed cantonal data to biozones while accounting for location of crop and fodder production areas in the 1985 land-use statistics (Bundesamt für Statistik, 2015). Interpolated missing years. Data per biozone relative to total biozone area.	Linear regression intercept per eight-year interval (year centred)
Mechanisation period	factor	*yes* or *no*				Graphically, intervals in which mechanisation (absolute) was changing most drastically were attributed to mechanisation periods (Supplementary Fig. 4)
Grassland area absolute	cont.	9.05–43.3 %	Data on the area of meadows and pastures per canton[1] from agricultural censuses[4]<br><br>Gridded data on summering pastures (hectare resolution) from land-use statistics (Bundesamt für Statistik, 2015)	1929[5], 1939, 1955, 1965, 1969, 1975, 1980, 1985, 1990, 1996–2021 (agr. censuses)<br><br>ca. 1982, ca. 1994, ca. 2006, ca. 2016 (land-use statistics)	Attributed cantonal census data of meadows and pastures to biozones while accounting for location of crop and fodder production areas in the 1985 land-use statistics (Bundesamt für Statistik, 2015). Interpolated missing years.<br>Aggregated gridded data of summering pastures to biozones. Interpolated and extrapolated missing years.<br>For extrapolation, we conservatively assumed no change (Supplementary Fig. 6).<br>Aggregated the two data sources by taking yearly sums per biozone. Data per biozone relative to total biozone area.	Linear regression intercept per eight-year interval (year centred)
Grassland area change	cont.	-0.356–0.190 %/yr				Linear regression slope per eight-year interval
Wood harvest intensity change	cont.	-0.378–0.491 m^3/ha/yr	Data on the area of forests and on harvested wood per canton[1] from silvicultural censuses[6]	1930–1934 1936–1950, 1951–1954 (wood harvest only), 1955, 1956–1957 (wood harvest only), 1958, 1959 (wood harvest only), 1960–2021	Attributed cantonal census data to biozones while accounting for location of forested areas in the 1985 land-use statistics (Bundesamt für Statistik, 2015). Interpolated forest area for years with only harvest data.<br><br>Wood harvest intensity was defined as harvested wood per forest area. For forest-	Linear regression slope of wood harvest intensity per eight-year interval after exclusion of storm years
Storm aftermath	factor	*yes* or *no*				Storm years were graphically identified as years with extraordinarily high wood harvests (Supplementary Fig. 5). Many instances could be confirmed with historical storm records. Intervals following storm years

					area variables, data per biozone was taken relative to the total biozone area.	were categorized as storm aftermath.
Forest area absolute	cont.	13.8–50.8 %				Linear regression intercept of forest area per eight-year interval (year centred)
Forest area change	cont.	-0.0316–2.25 %/yr				Linear regression slope of forest area per eight-year interval. Cube-root transformed to restrict effect of outliers (cf. Supplementary Fig. 7).

[1]The canton of Jura was only founded in 1979, before the territory belonged to the canton of Berne. For years without data for Jura, data from Berne were attributed to Jura based on proportions between the cantons after the separation.

[2]Census data from 1971 onwards available from the Federal Statistical Office through https://www.pxweb.bfs.admin.ch/pxweb/en/px-x-0102020000_101/-/px-x-0102020000_101.px/. Data before was taken from: Eidgenössisches Statistisches Amt. ‘Statistisches Jahrbuch Der Schweiz 1971’. Statistisches Jahrbuch der Schweiz. Bern, Switzerland, 1971. https://www.bfs.admin.ch/bfs/de/home/statistiken/kataloge-datenbanken/publikationen/uebersichtsdarstellungen/statistisches-jahrbuch.assetdetail.346211.html

[3]Census data from 1990 onwards available from the Federal Statistical Office through https://www.pxweb.bfs.admin.ch/pxweb/en/px-x-1103020100_143/-/px-x-1103020100_143.px/. Data before was gathered from:

Schweizerisches Bauernsekretariat. ‘Statistische Erhebungen Und Schätzungen Auf Dem Gebiete Der Landwirtschaft 1950’. Jahresheft. Brugg, Switzerland: Verlag des Schweizerischen Bauernsekretariates, 1950. https://www.sbv-usp.ch/fileadmin/sbvuspch/04_Medien/Publikationen/SES/Archiv/SES_1950-27.pdf.

Schweizerisches Bauernsekretariat. ‘Statistische Erhebungen Und Schätzungen Auf Dem Gebiete Der Landwirtschaft 1956’. Jahresheft. Brugg, Switzerland: Verlag des Schweizerischen Bauernsekretariates, 1956. https://www.sbv-usp.ch/fileadmin/sbvuspch/04_Medien/Publikationen/SES/Archiv/SES_1956-33.pdf.

Schweizerisches Bauernsekretariat. ‘Statistische Erhebungen Und Schätzungen Über Landwirtschaft Und Ernährung 1970’. Jahresheft. Brugg, Switzerland: Verlag des Schweizerischen Bauernsekretariates, 1970. https://www.sbv-usp.ch/fileadmin/sbvuspch/04_Medien/Publikationen/SES/Archiv/SES_1970-47.pdf.

Schweizerisches Bauernsekretariat. ‘Statistische Erhebungen Und Schätzungen Über Landwirtschaft Und Ernährung 1981’. Jahresheft. Brugg, Switzerland: Verlag des Schweizerischen Bauernsekretariates, 1981. https://www.sbv-usp.ch/fileadmin/sbvuspch/04_Medien/Publikationen/SES/Archiv/SES_1981-58.pdf.

Schweizerisches Bauernsekretariat. ‘Statistische Erhebungen Und Schätzungen Über Landwirtschaft Und Ernährung 1989’. Jahresheft. Brugg, Switzerland: Verlag des Schweizerischen Bauernsekretariates, 1989. https://www.sbv-usp.ch/fileadmin/sbvuspch/04_Medien/Publikationen/SES/Archiv/SES_1989-66.pdf.

[4]Census data from 1975 onwards available from the Federal Statistical Office through https://www.pxweb.bfs.admin.ch/pxweb/en/px-x-0702000000_102/-/px-x-0702000000_102.px/. Data before was gathered from:

Eidgenössisches Statistisches Amt. ‘Die Landwirtschaftsbetriebe in Der Schweiz. Tabellenteil. Band 6 Der Eidgenössischen Betriebszählung 1929’. Statistische Quellenwerke der Schweiz. Bern, Switzerland, 1933. https://www.bfs.admin.ch/bfs/de/home/statistiken/katalog.assetdetail.265893.html.

Eidgenössisches Statistisches Amt. ‘Bodenbenützung 1939 Und Ackerbau 1940/43 in Der Schweiz. Ergebnisse Der Eidgenössischen Betriebszählunh 1939 Und Der Anbauerhebungen 1940, 1941, 1942 Und 1943.’ Statistische Quellenwerke der Schweiz. Bern, Switzerland, 1943. https://www.bfs.admin.ch/bfs/de/home/statistiken/katalog.assetdetail.246537.html.

Eidgenössisches Statistisches Amt. ‘Bodenbenützung in Der Schweiz 1955’. Statistische Quellenwerke der Schweiz. Bern, Switzerland, 1959. https://www.bfs.admin.ch/bfs/de/home/statistiken/katalog.assetdetail.265277.html.

Eidgenössisches Statistisches Amt. ‘Bodenbenützung 1965’. Statistische Quellenwerke der Schweiz. Bern, Switzerland, 1967. https://www.bfs.admin.ch/bfs/de/home/statistiken/katalog.assetdetail.265151.html.

Eidgenössisches Statistisches Amt. ‘Bodenbenützung 1969’. Statistische Quellenwerke der Schweiz. Bern, Switzerland, 1970. https://www.bfs.admin.ch/bfs/de/home/statistiken/katalog.assetdetail.265161.html.

[5]No data on area of pastures available for 1929. Estimates from area of meadows based on odds in 1939.

[6]Census data from 1975 onwards available from the Federal Statistical Office through https://www.pxweb.bfs.admin.ch/pxweb/en/px-x-0703010000_101/px-x-0703010000_101/px-x-0703010000_101.px and https://www.pxweb.bfs.admin.ch/pxweb/en/px-x-0703010000_102/-/px-x-0703010000_102.px/. Data before was gathered from the statistical yearbooks 1931–1976, which are available from https://www.bfs.admin.ch/bfs/de/home/statistiken/kataloge-datenbanken/publikationen/uebersichtsdarstellungen/statistisches-jahrbuch.html. Data from all sources was divided by publicly and privately owned forests, but data on area and harvest from private forests was missing from years prior to 1950. For missing years, area and harvest from private forests were estimated from average odds of the years 1950–1955 for each biozone.

Supplementary Table 4 | Overview of model priors. Priors for the parameters of the occupancy-detection models and the regression models relating richness trends to environmental variables.

Model	Parameter	Meaning	Prior
occupancy-detection models	μ_o	global intercept (occ.)	$\mathrm{Normal}(0, 1.5^2)$
occupancy-detection models	$\sigma_{\gamma r}$	SD of year random effect (t > 1930/31) (occ.)	$\mathrm{Cauchy}(0,1)$
occupancy-detection models	$\gamma_{r,1}$	Two-year interval 1930/31 random effect (occ.)	$\mathrm{Normal}(0, 1.5^2)$
occupancy-detection models	σ_o	SD of random effects (occ.)	$\mathrm{Cauchy}(0,1)$
occupancy-detection models	β_o	slope of fixed effects (occ.)	$\mathrm{Normal}(0, 5^2)$
occupancy-detection models	μ_d	global intercept (det.)	$\mathrm{Normal}(0, 1.5^2)$
occupancy-detection models	σ_d	SD of random effects (det.)	$\mathrm{Cauchy}(0,1)$
occupancy-detection models	β_d	slope of fixed effects (det.)	$\mathrm{Normal}(0, 5^2)$
regression models	μ	global intercept	$\mathrm{Normal}(0, 5^2)$
regression models	β	slope fixed effects	$\mathrm{Normal}(0, 5^2)$
regression models	σ_r	SD of random effects	$\mathrm{Cauchy}(0,1)$
regression models	σ_y	scale parameter (Student's t-distribution)	$\mathrm{Cauchy}(0,25)$
regression models	ν_y	Degrees of freedom (Student's t-distribution)	$\mathrm{Gamma}(2,0.1)$

Supplementary Figures

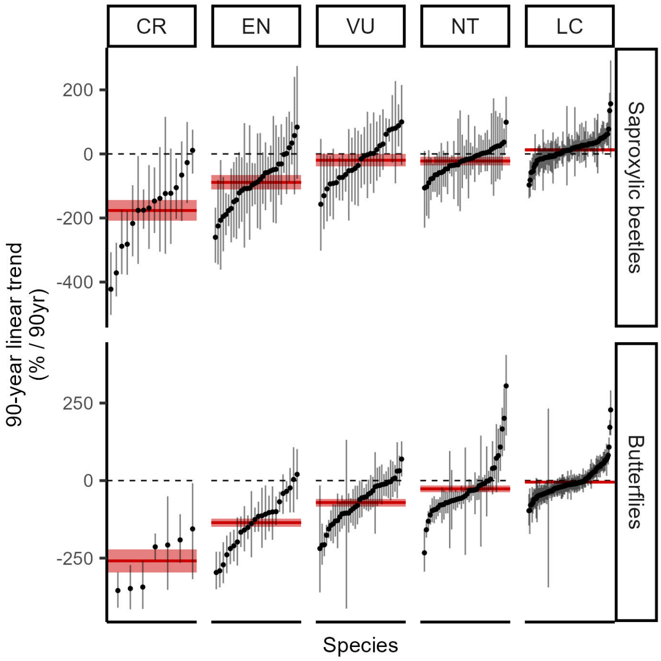

Supplementary Fig. 1 | Comparison of species-level occupancy trend estimates with Red List status. For 198 saproxylic beetle and 214 butterfly species, the 90-year trends in mean occupancy are shown, separated by their Red List status (Monnerat et al., 2016; Wermeille et al., 2014). Trend estimates are based on linear regressions of mean occupancy against years. Mean occupancy was scaled per species relative to the mean occupancy over the 90 study years (in %). The trend estimates denote the change in percentage of a species' distribution over the whole 90 years. Regressions were fitted at the level of MCMC iterations. Each point shows the mean across all iterations for one species and the vertical lines show the 95% credible intervals. The red lines and ribbons show the mean across all species of one status and group based on 5000 MCMC simulations (mean and 95% credible intervals). CR: critically endangered; EN: endangered; VU: vulnerable; NT: near threatened; LC: least concern.

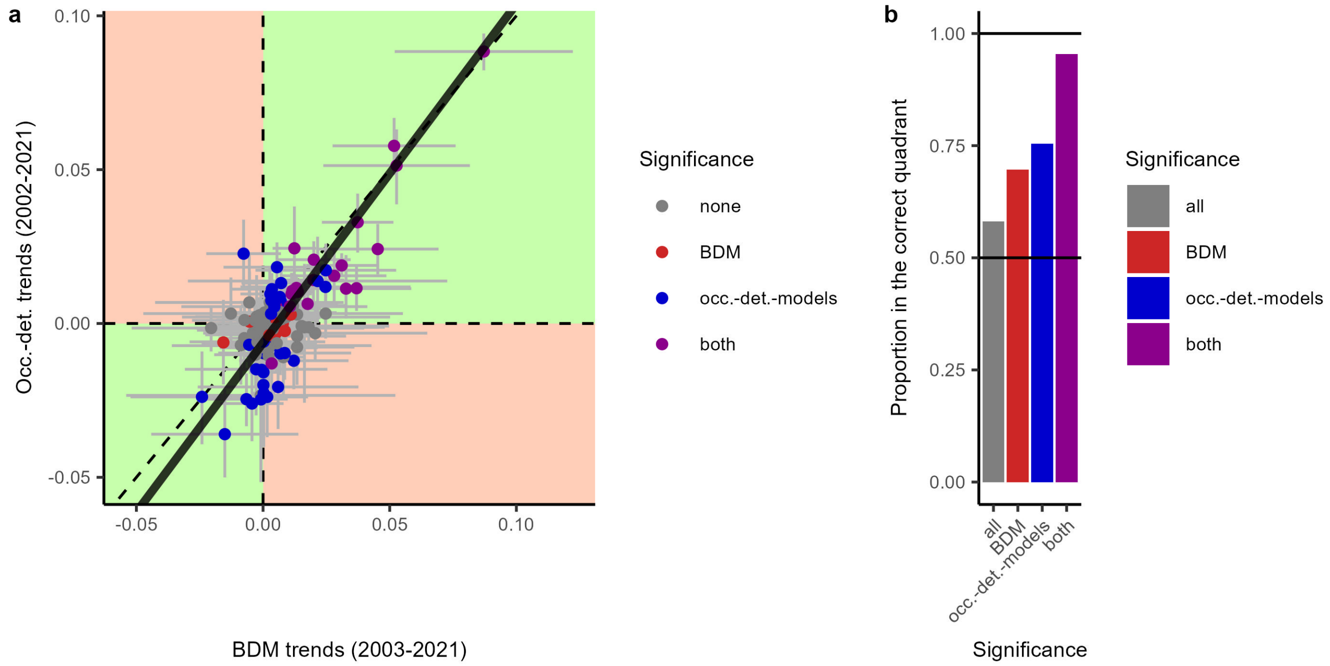

Supplementary Fig. 2 | Species-level comparison of butterfly trends from occupancy-detection models and from standardised sampling. First, linear trends were calculated from mean occupancy across Switzerland based on occupancy-detection models. Occupancy-detection models had the same structure as the models used for the main analyses but were restricted to the period 1990–2021 and did not include data from the Swiss biodiversity monitoring program (BDM; Koordinationsstelle BDM, 2004) to prevent circularity in the analyses. The species-level trends were based on linear model coefficients for the period 2002–2021 (2002 included because of two-year intervals). Second, linear trends were calculated from data collected in the standardised BDM scheme (Koordinationsstelle BDM, 2004), which started in 2003. Each species' distribution was calculated as the proportion of occupied monitoring squares (1 km × 1 km) in a given year. This distribution was analysed in linear models for the years 2003–2021. Only species covered in both datasets were included (129 species). To make trends comparable between datasets, they were calculated based on a species' distribution relative to its mean distribution across all included years (change in proportion per year). **a** Comparison of the two trends per species. Each point is a species, the colour denotes the significance (either significant in none of the datasets, in one of the two, or in both). Significance for the BDM trends was based on the model *P* value (alpha level of 0.05). For the occupancy trends, it was based on the 95%-credible intervals. Grey lines show the 95%-confidence intervals (BDM) and 95% credible intervals (occ.-det.). The solid line shows a trend line based on all observations from a Deming regression (Therneau, 2024). **b** Proportion of species that are in the correct quadrant in **a** (green quadrants; both datasets indicating either declines or increases), shown for different combinations of significance (all points, one of the two datasets significant, both datasets significant).

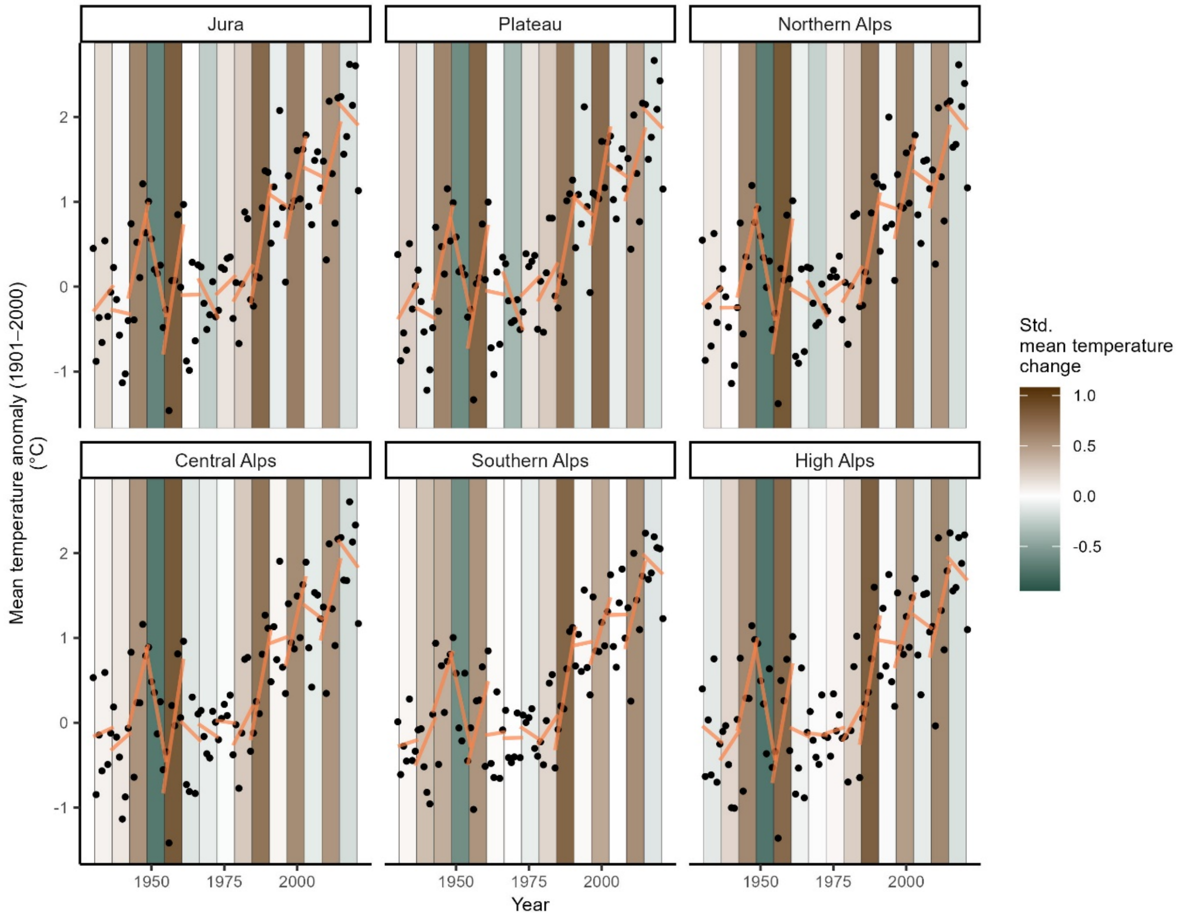

Supplementary Fig. 3 | Change in the anomaly of mean annual temperature between 1930 and 2021. For each of the six biozones, the average anomaly of the (reconstructed) mean annual temperature in relation to the mean of the years 1901–2000 is shown (points). Orange lines show the linear regression lines for the eight-year intervals (1930–1937, 1936–1943, etc.), based on which the variables *Temperature absolute* (centred intercept) and *Temperature change* (slope) were determined. The underlying shading indicates the mean temperature change (slope) per interval, standardised to a standard deviation of 0.5.

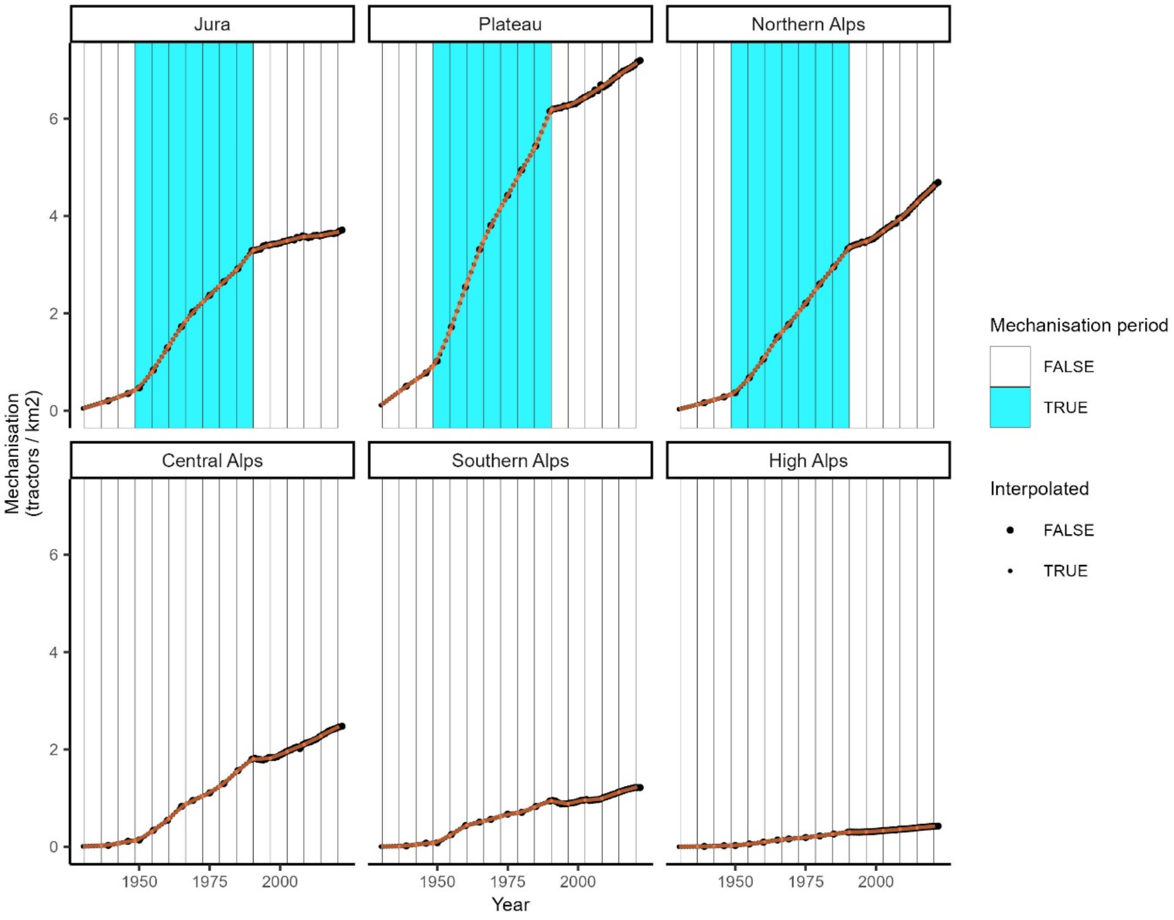

Supplementary Fig. 4 | Change in the agricultural mechanisation between 1930 and 2021. For each of the six biozones, the density of tractors is shown for the years 1930 to 2021 (points). Larger points are years, for which data could be retrieved. For years in between, data were interpolated (smaller points). Orange lines show the linear regression lines for the eight-year intervals (1930–1937, 1936–1943, etc.), based on which the variable *Mechanisation absolute* (centred intercept) was determined. The variable *Mechanisation period* was integrated as a factor variable denoting the intervals of highest mechanisation in the three biozones of highest mechanisation degree (turquoise shading).

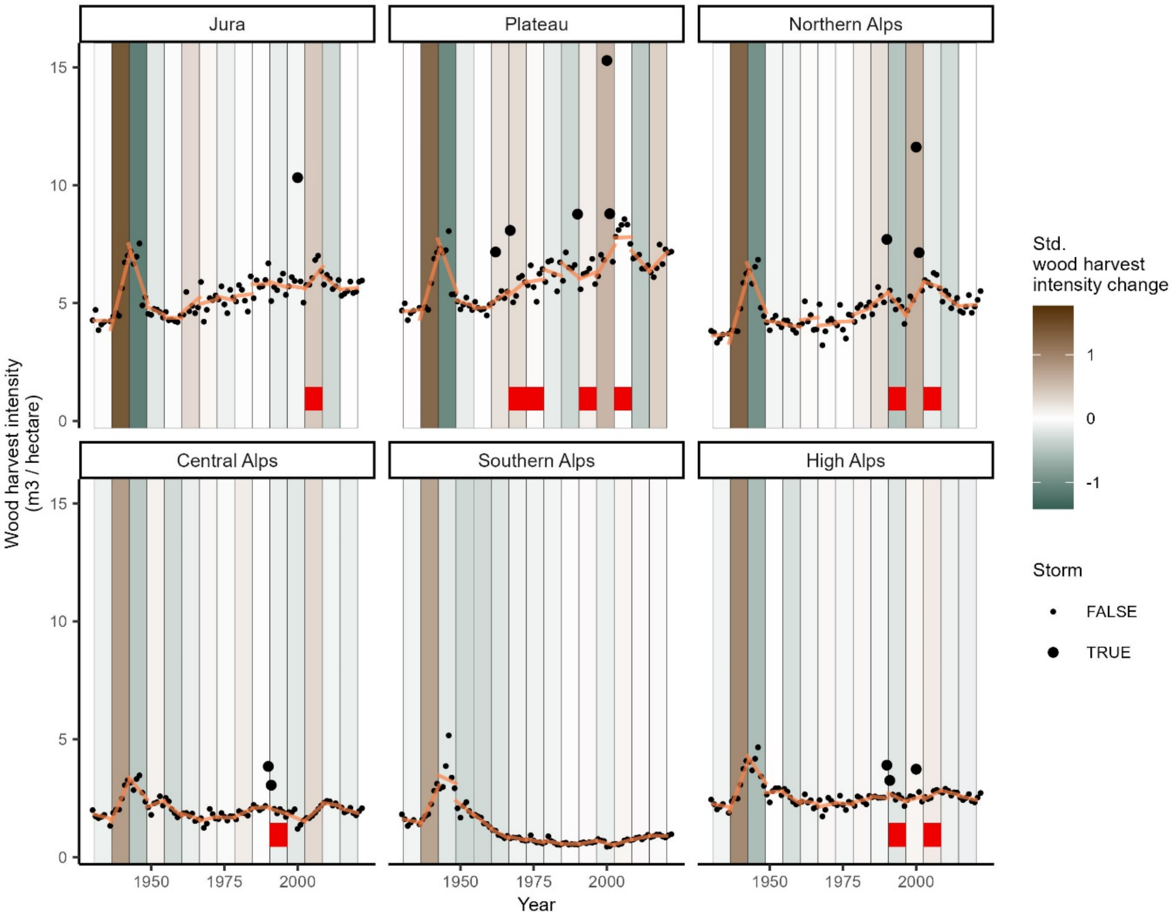

Supplementary Fig. 5 | Change in the wood harvest intensity and storm events between 1930 and 2021. For each of the six biozones, the wood harvest intensity (harvested wood per managed forest area) is shown for the years 1930 to 2021 (points). Larger points show outliers, which were indicative of large storm events. Orange lines show the linear regression lines for the eight-year intervals (1930–1937, 1936–1943, etc.), based on which the variable *Wood harvest intensity change* (slope) was determined (after exclusion of storm years). The underlying shading indicates the wood harvest intensity change (slope) per interval, standardised to a standard deviation of 0.5. Intervals following a storm year were characterised by the factor variable *Storm aftermath*, as indicated by the red shading at the bottom of the respective intervals. Data on forest area for the years 1951–1954, 1956, 1957 and 1959 were not available and were interpolated from adjacent years (cf. Supplementary Fig. 7).

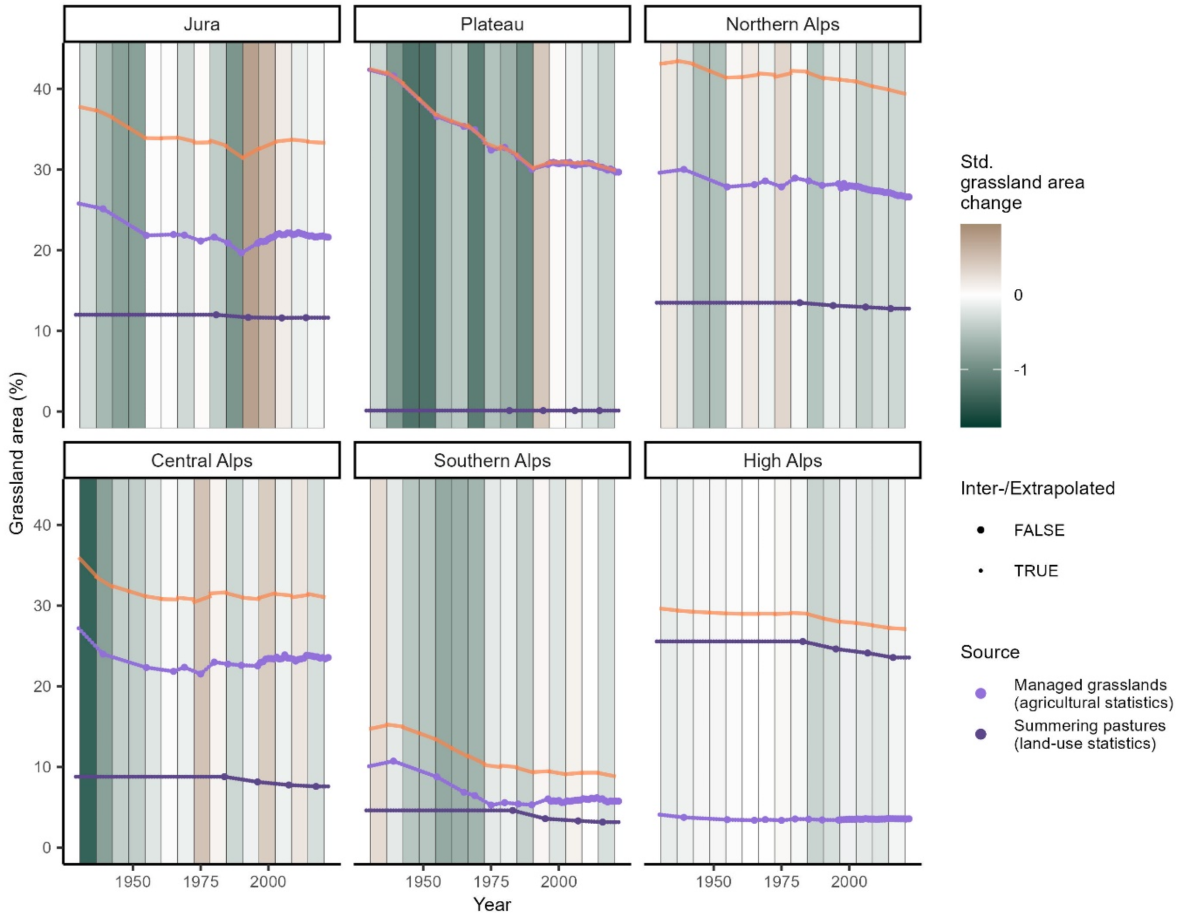

Supplementary Fig. 6 | Change in the grassland area share between 1930 and 2021. For each of the biozones, the share of grasslands of the total area is shown for the years 1930 to 2021. Grassland area data were retrieved from two sources. On the one hand, data from agricultural statistics on the amounts of managed grasslands (not including summering pastures) was collected (light purple points). On the other hand, data from land-use statistics ("Arealstatistik") on the amounts of summering pastures was collected (dark purple point). Data were only available for a subset of the study years, as indicated by point size. Smaller points denote years, for which data were inter- or extrapolated. Based on the sum of the two data sources, linear regressions were done for each eight-year interval (1930–1937, 1936–1943, etc.), shown by the orange lines. From these regressions, the variables *Grassland area absolute* (centred intercept) and *Grassland area change* (slope) were determined. The underlying shading indicates the grassland area change (slope) per interval, standardised to a standard deviation of 0.5.

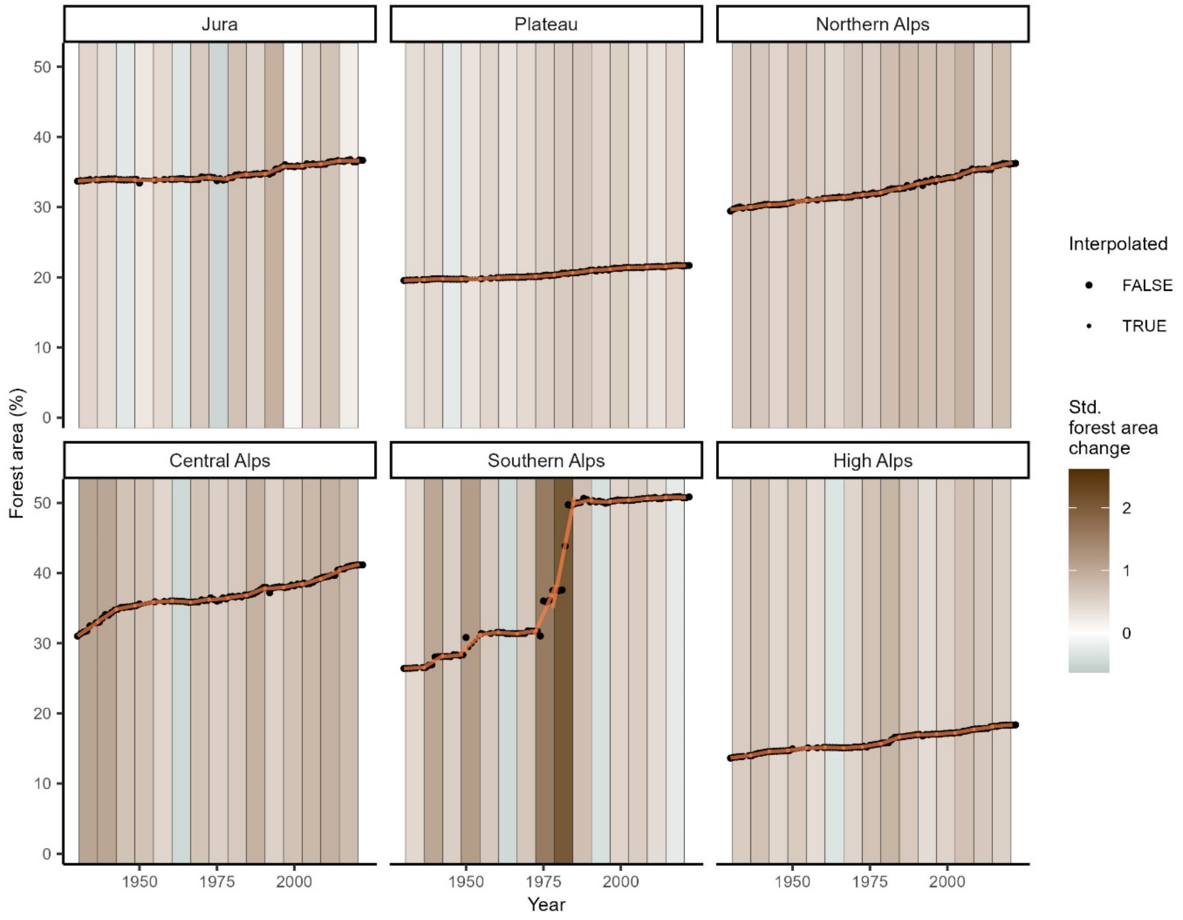

Supplementary Fig. 7 | Change in the forest area between 1930 and 2021. For each of the six biozones, the share of forests of the total area is shown for the years 1930 to 2021 (points). Smaller points show years, for which data were not available and thus interpolated from adjacent years (1951–1954, 1956, 1957 and 1959). Orange lines show the linear regression lines for the eight-year intervals (1930–1937, 1936–1943, etc.), based on which the variables *Forest area absolute* (centred intercept) and *Forest area change* (slope) were determined. Forest area change was further transformed by taking the cube root to reach a better distribution of the variable. The underlying shading indicates the transformed forest area change per interval, standardised to a standard deviation of 0.5.

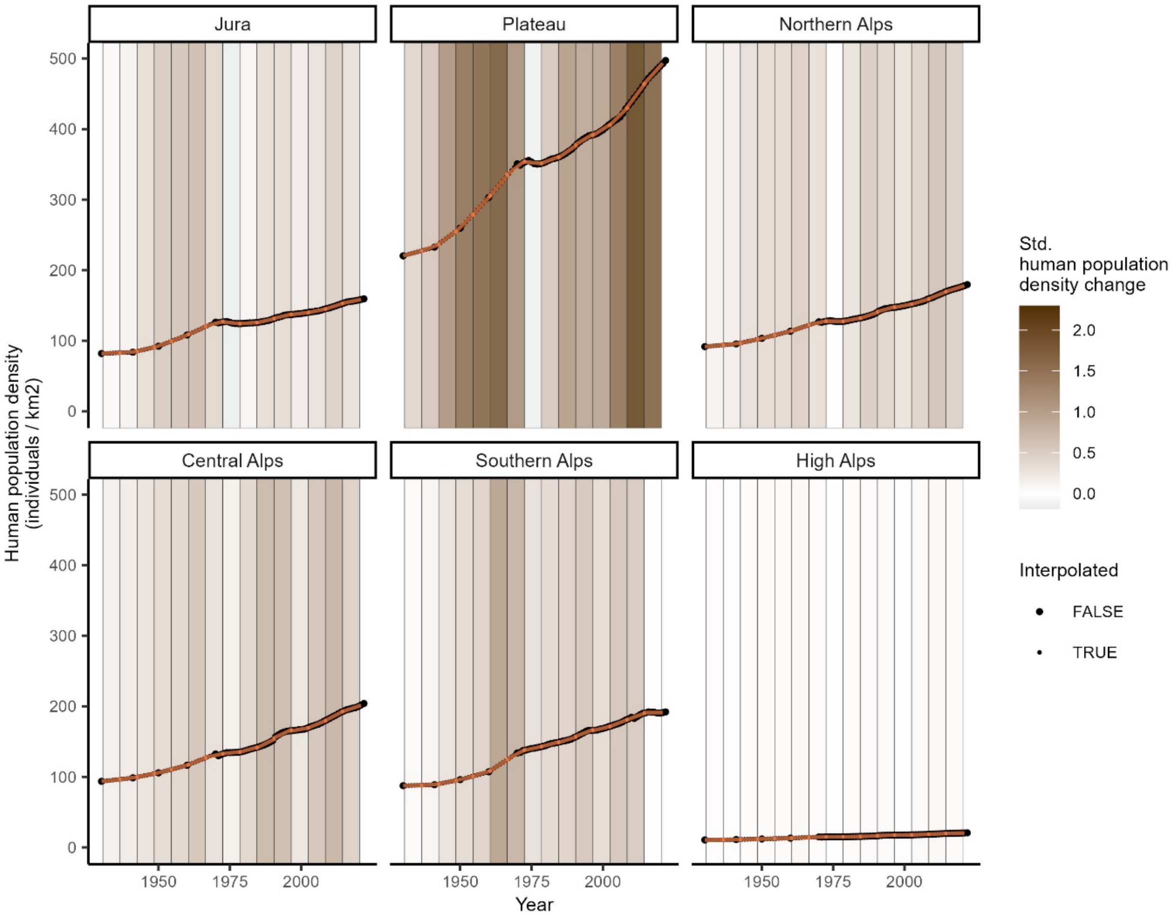

Supplementary Fig. 8 | Change in the human population density between 1930 and 2021. For each of the six biozones, the population density is shown for the years 1930 to 2021 (points). Larger points are years, for which data could be retrieved. For years in between, data were interpolated (smaller points). Orange lines show the linear regression lines for the eight-year intervals (1930–1937, 1936–1943, etc.), based on which the variable *Human population density change* (slope) was determined. The underlying shading indicates the human population density change (slope) per interval, standardised to a standard deviation of 0.5.

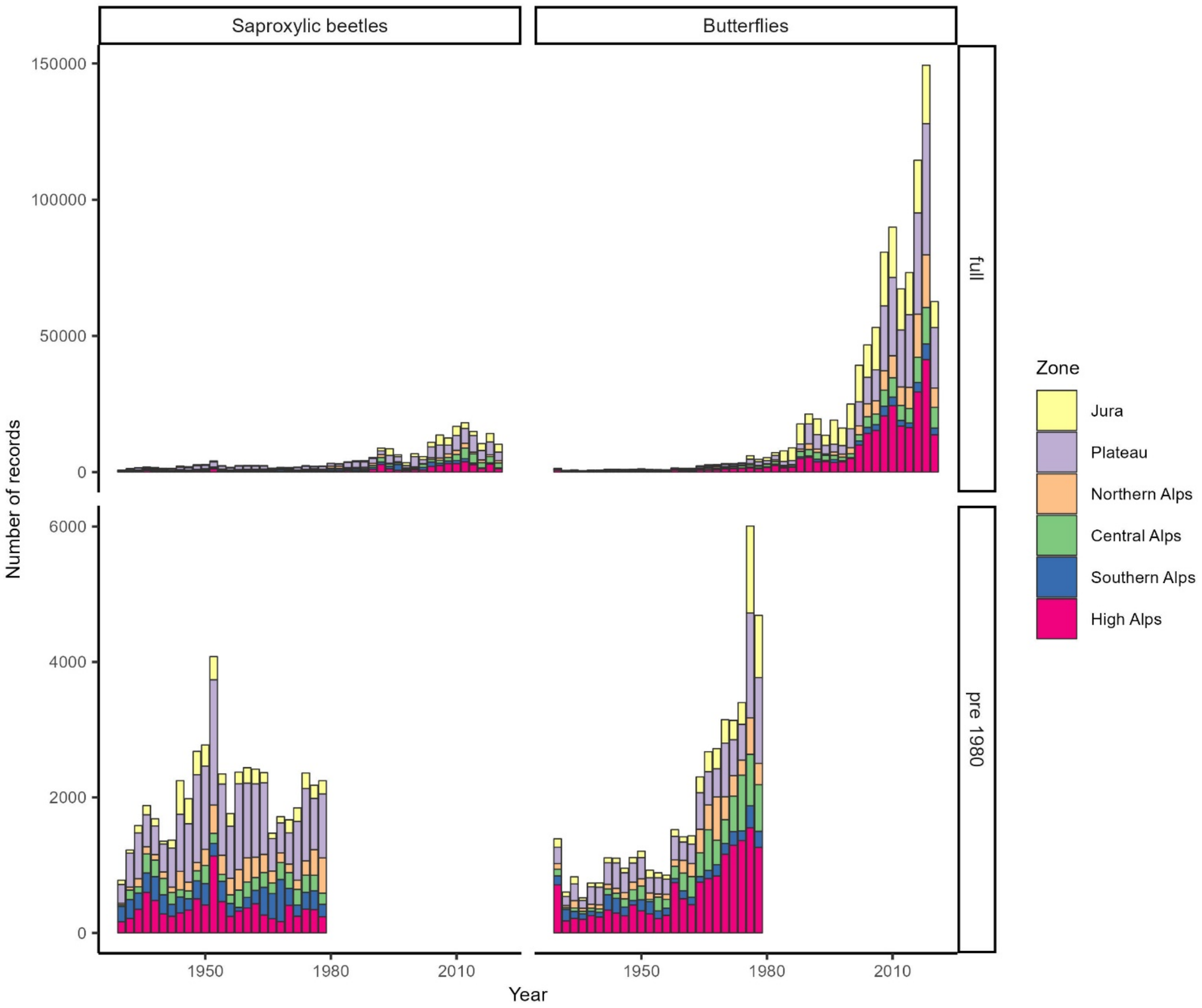

Supplementary Fig. 9 | Number of records that could be analysed per group. Number of records per two-year entity, which were part of the final dataset that was analysed in occupancy-detection models. The upper panels show the full range of years, the lower panels are a zoomed in version for the years prior to 1980. Colours indicate the biozone, from which the record originates.

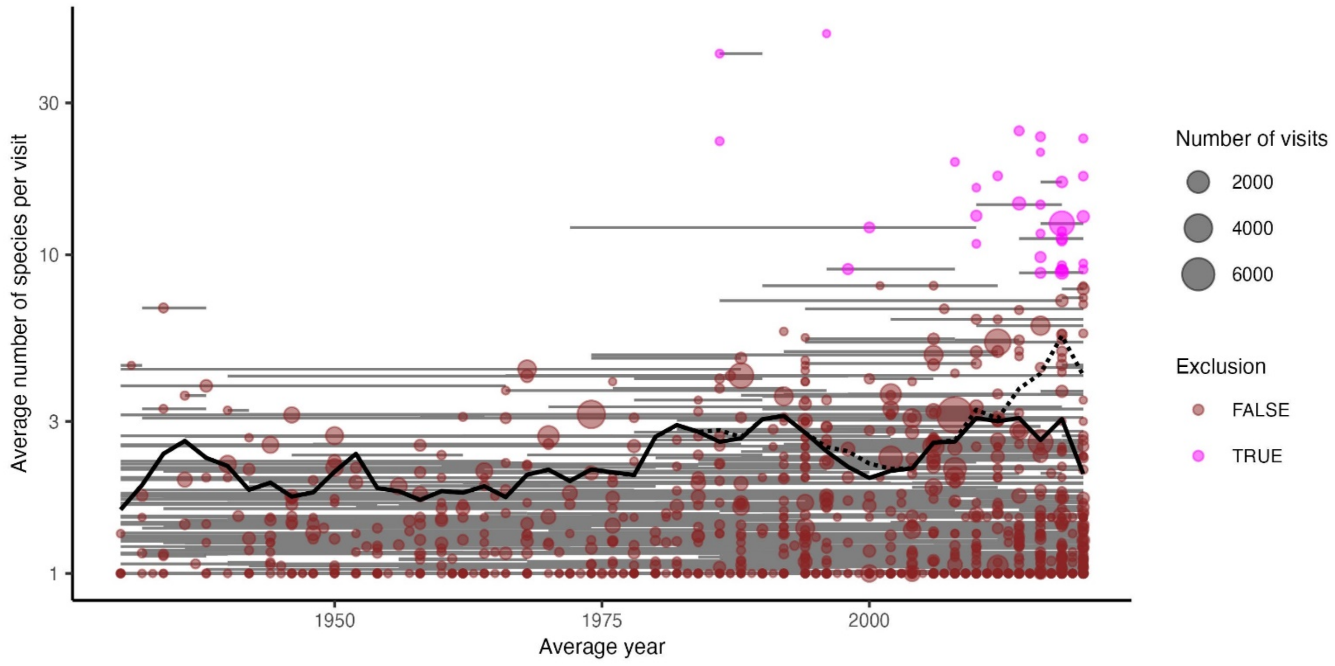

Supplementary Fig. 10 | Number of beetle species recorded in a visit per observer. Each point shows an observer (project or naturalist) and the average number of species this observer recorded per visit. The mean year of the observer's visits are on the *x* axis, the point size indicates the number of visits that were contributed by the observer. Grey horizontal lines show the range of years, for which records of an observer were available. Records from observers with an average number of species per visit above the 97.5% quantile (after exclusion of observers with only one visit) were excluded from the analyses (in magenta). The black lines show the average number of species per visit without (solid) and with (dotted) the excluded observer per year. Note that the *y* axis is on log scale.

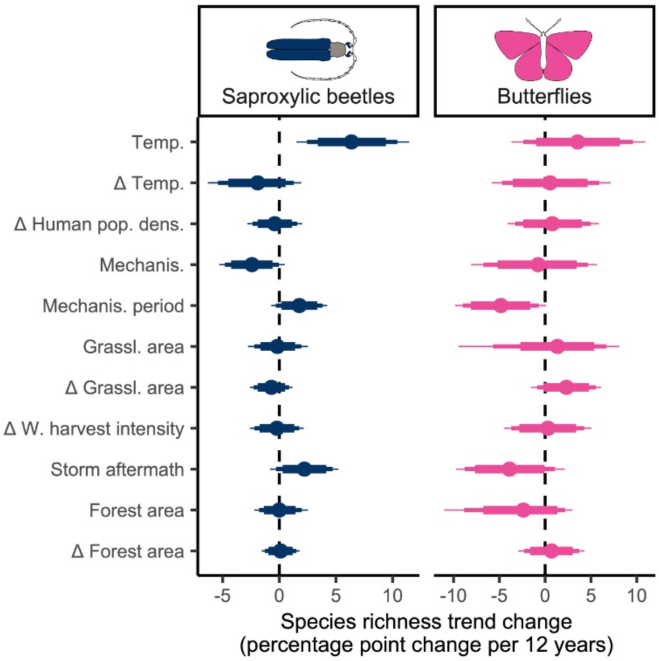

Supplementary Fig. 11 | Regression model results linking relative richness trends to (changes of) environmental conditions at twelve-year intervals. Posterior distributions of slope estimates from regression models relating the trend of relative richness in a biozone and twelve-year interval to a set of environmental variables for the same zone and interval. Slope estimates were transformed to represent the change in the species richness trend in a twelve-year interval (cf. Fig. 3). Separate models were fitted for saproxylic beetles (blue) and butterflies (pink). Continuous predictor variables were scaled to SD 0.5 prior to analyses. Points show means, segments of varying thickness show 80%-, 90%- and 95%-credible intervals. $n = 54$ observations (biozone-interval combinations) per model. Temp.: temperature absolute, Δ Temp.: temperature change; Δ Human pop. dens.: Human population density change, Mechanis: mechanisation absolute, Mechanis. period: Mechanisation period (factor), Grassl. area: grassland area absolute, Δ Grassl. Area: grassland area change, Δ W. harvest intensity: wood harvest intensity change.

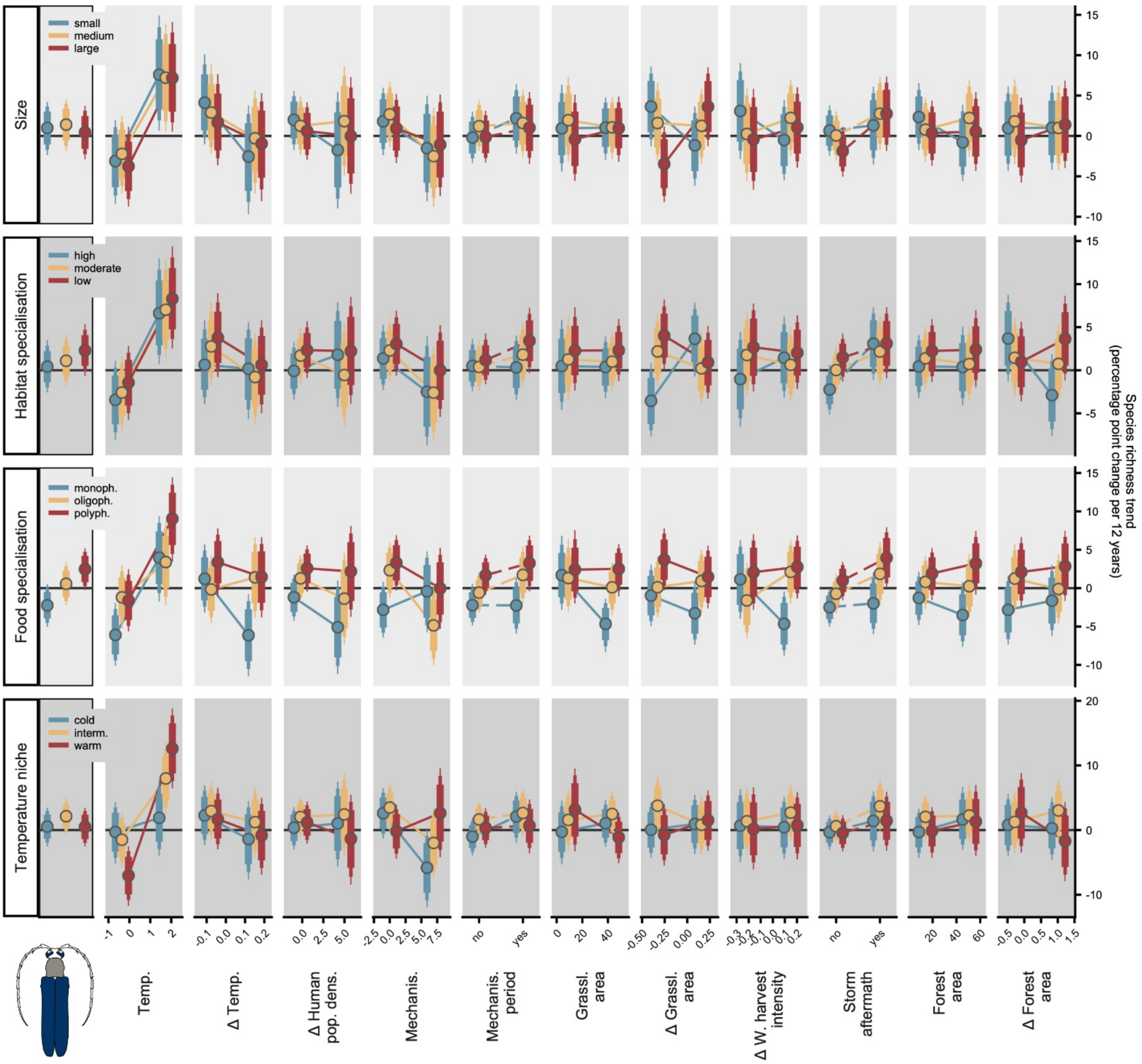

Supplementary Fig. 12 | Predictions from models relating saproxylic beetle occupancy changes to environmental variables and traits at twelve-year intervals. Regression model predictions for models relating the trend of relative richness of saproxylic beetles (separated by different trait values) in a biozone and twelve-year interval to the environmental conditions and changes therein in the same zone, twelve-year interval and to different trait values. Also, models included interactions between all environmental variables and the trait values. One model was fitted for each trait (rows). The predictions show the expected richness trend. The panels at the very left show the main effect of the different trait values, the remaining panels illustrate the interactive effects between trait value and environmental variables. Points show means, segments of varying thickness show 80%-, 90%- and 95%-credible intervals. *n* = 162 observations (biozone-interval-traitvalue combinations) per model. Temp.: temperature absolute, Δ Temp.: temperature change; Δ Human pop. dens.: Human population density change, Mechanis: mechanisation absolute, Mechanis. period: Mechanisation period (factor), Grassl. area: grassland area absolute, Δ Grassl. Area: grassland area change, Δ W. harvest intensity: wood harvest intensity change

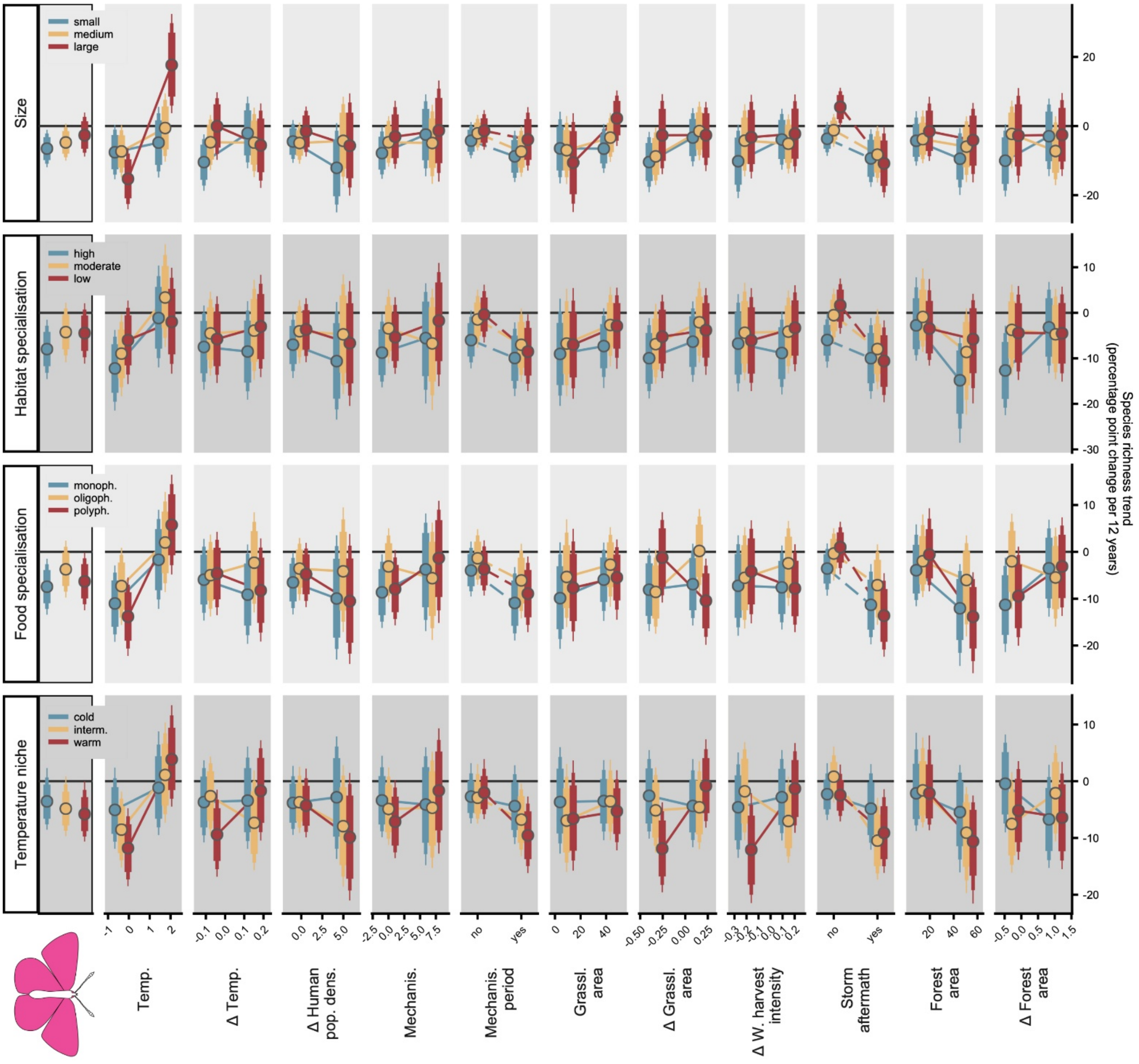

Supplementary Fig. 13 | Predictions from models relating butterfly occupancy changes to environmental variables and traits at twelve-year intervals. Regression model predictions for models relating the trend of relative richness of butterflies (separated by different trait values) in a biogeozone and twelve-year interval to the environmental conditions and changes therein in the same zone, twelve-year interval and to different trait values. Also, models included interactions between all environmental variables and the trait values. One model was fitted for each trait (rows). The predictions show the expected richness trend. The panels at the very left show the main effect of the different trait values, the remaining panels illustrate the interactive effects between trait value and environmental variables. Points show means, segments of varying thickness show 80%-, 90%- and 95%-credible intervals. n = 162 observations (biozone-interval-traitvalue combinations) per model. Temp.: temperature absolute, Δ Temp.: temperature change; Δ Human pop. dens.: Human population density change, Mechanis: mechanisation absolute, Mechanis. period: Mechanisation period (factor), Grassl. area: grassland area absolute, Δ Grassl. Area: grassland area change, Δ W. harvest intensity: wood harvest intensity change

Supplementary References

Bowler, D. E., Eichenberg, D., Conze, K.-J., Suhling, F., Baumann, K., Benken, T., Bönsel, A., Bittner, T., Drews, A., Günther, A., Isaac, N. J. B., Petzold, F., Seyring, M., Spengler, T., Trockur, B., Willigalla, C., Bruelheide, H., Jansen, F., & Bonn, A. (2021). Winners and losers over 35 years of dragonfly and damselfly distributional change in Germany. *Diversity and Distributions*, *27*(8), 1353–1366. https://doi.org/10.1111/ddi.13274

Bundesamt für Statistik. (2015). *Arealstatistik nach Nomenklatur 2004 – Bodennutzung (Land Use)*. Bundesamt für Statistik, Neuchâtel, Switzerland.

Bundesamt für Umwelt. (2022). *Die biogeografischen Regionen der Schweiz. 1. Aktualisierte Auflage 2022.* (No. 2214; Umwelt-Wissen, p. 28). Bundesamt für Umwelt, Bern, Switzerland.

Gabry, J., Češnovar, R., Bales, B., Morris, M., Popov, M., Lawrence, M., & Landau, W. M. (2021). *cmdstanr: R interface to "CmdStan". R package version 0.4.0* [Computer software]. https://mc-stan.org/cmdstanr/

Guo, J., Gabry, J., Goodrich, B., Weber, S., Lee, D., Sakredja, K., Martin, M., Trustees of Columbia University, Sklyar, O., The R Core Team, Oehlschlaegel-Akiyoshi, J., Maddock, J., Bristow, P., Agrawal, N., Kormanyos, C., & Steve, B. (2020). *rstan: R interface to Stan. R package version 2.21.2* [Computer software]. https://CRAN.R-project.org/package=rstan

Koordinationsstelle BDM. (2004). *Biodiversitätsmonitoring Schweiz BDM. Beschreibung der Methoden und Indikatoren* (No. 1410; Umwelt-Wissen, p. 104). Bundesamt für Umwelt. Bern, Switzerland.

Monnerat, C., Barbalat, S., Lachat, T., & Gonseth, Y. (2016). *Rote Liste der Prachtkäfer, Bockkäfer, Rosenkäfer und Schröter. Gefährdete Arten der Schweiz* (No. 1622; Umwelt- Vollzug, p. 118). Bundesamt für Umwelt / Info Fauna – CSCF / Eidg. Forschungsanstalt WSL, Bern / Neuchâtel / Birmensdorf, Switzerland.

Neff, F., Korner-Nievergelt, F., Rey, E., Albrecht, M., Bollmann, K., Cahenzli, F., Chittaro, Y., Gossner, M. M., Martínez-Núñez, C., Meier, E. S., Monnerat, C., Moretti, M., Roth, T., Herzog, F., & Knop, E. (2022). Different roles of concurring climate and regional land-use changes in past 40 years' insect trends. *Nature Communications*, *13*(1), 7611. https://doi.org/10.1038/s41467-022-35223-3

Outhwaite, C. L., Chandler, R. E., Powney, G. D., Collen, B., Gregory, R. D., & Isaac, N. J. B. (2018). Prior specification in Bayesian occupancy modelling improves analysis of species occurrence data. *Ecological Indicators*, *93*, 333–343. https://doi.org/10.1016/j.ecolind.2018.05.010

Outhwaite, C. L., Powney, G. D., August, T. A., Chandler, R. E., Rorke, S., Pescott, O. L., Harvey, M., Roy, H. E., Fox, R., Roy, D. B., Alexander, K., Ball, S., Bantock, T., Barber, T., Beckmann, B. C., Cook, T., Flanagan, J., Fowles, A., Hammond, P., … Isaac, N. J. B. (2019). Annual estimates of occupancy for bryophytes, lichens and invertebrates in the UK, 1970–2015. *Scientific Data*, *6*(1), 259. https://doi.org/10.1038/s41597-019-0269-1

Therneau, T. (2024). *deming: Deming, Theil-Sen, Passing-Bablock and Total Least Square. R package version 1.4-1* [Computer software]. https://CRAN.R-project.org/package=deming

Wermeille, E., Chittaro, Y., & Gonseth, Y. (2014). *Rote Liste Tagfalter und Widderchen. Gefährdete Arten der Schweiz, Stand 2012* (No. 1403; Umwelt-Vollzug, p. 97). Bundesamt für Umwelt / Schweizer Zentrum für die Kartografie der Fauna, Bern / Neuchâtel, Switzerland.
