# Supplementary material for: Ninety-year trends reveal sharpest insect declines in the mid-twentieth century
Source: Nat Ecol Evol. 2026 Jun 2;10(6):1103–13. doi: 10.1038/s41559-026-03074-6 (PMC13253333; doi:10.1038/s41559-026-03074-6)
Supplement: Supplementary file 2 — Reporting Summary [file 41559_2026_3074_MOESM2_ESM.pdf]

nature portfolio

Corresponding author(s): Felix Neff

Last updated by author(s): Mar 31, 2026

Reporting Summary

Nature Portfolio wishes to improve the reproducibility of the work that we publish. This form provides structure for consistency and transparency in reporting. For further information on Nature Portfolio policies, see our Editorial Policies and the Editorial Policy Checklist.

Statistics

For all statistical analyses, confirm that the following items are present in the figure legend, table legend, main text, or Methods section.

n/a	Confirmed	
☐	☒	The exact sample size (*n*) for each experimental group/condition, given as a discrete number and unit of measurement
☒	☐	A statement on whether measurements were taken from distinct samples or whether the same sample was measured repeatedly
☐	☒	The statistical test(s) used AND whether they are one- or two-sided<br>*Only common tests should be described solely by name; describe more complex techniques in the Methods section.*
☐	☒	A description of all covariates tested
☐	☒	A description of any assumptions or corrections, such as tests of normality and adjustment for multiple comparisons
☐	☒	A full description of the statistical parameters including central tendency (e.g. means) or other basic estimates (e.g. regression coefficient) AND variation (e.g. standard deviation) or associated estimates of uncertainty (e.g. confidence intervals)
☒	☐	For null hypothesis testing, the test statistic (e.g. *F*, *t*, *r*) with confidence intervals, effect sizes, degrees of freedom and *P* value noted<br>*Give P values as exact values whenever suitable.*
☐	☒	For Bayesian analysis, information on the choice of priors and Markov chain Monte Carlo settings
☐	☒	For hierarchical and complex designs, identification of the appropriate level for tests and full reporting of outcomes
☒	☐	Estimates of effect sizes (e.g. Cohen's *d*, Pearson's *r*), indicating how they were calculated

Our web collection on statistics for biologists contains articles on many of the points above.

Software and code

Policy information about availability of computer code

Data collection	For some steps during data collection, we used R version 4.3.3
Data analysis	All relevant codes used to complete this work are deposited in a GitHub repository (https://github.com/nefff1/insect_trends_1930) available from Zenodo (https://doi.org/10.5281/zenodo.17256301). All data analyses were done with R version 4.0.2 and higher and with Stan version 2.27.0 and higher. The following R packages were key: bayestestR (v. 1.8-12), brms (2.21.0), cmdstanr (v. 0.4.0), cowplot (v. 1.1.3), data.table (v. 1.15.4), DHARMa (v. 0.4.6), ggh4x (v. 0.2.8), ggpubr (v. 0.6.0), giscoR (v. 0.6.0), lubridate (v. 1.9.3), ncdf4 (v. 1.23), posterior (v. 0.1.5) , raster (v. 3.6-26), rstan (v. 2.21.2) , sf (v. 1.0-15), sfheaders (v. 0.4.4), stars (v. 0.6-6), tidyverse (v. 2.0.0), zoo (v. 1.8-12)

For manuscripts utilizing custom algorithms or software that are central to the research but not yet described in published literature, software must be made available to editors and reviewers. We strongly encourage code deposition in a community repository (e.g. GitHub). See the Nature Portfolio guidelines for submitting code & software for further information.

Data

Policy information about availability of data

All manuscripts must include a data availability statement. This statement should provide the following information, where applicable:

- Accession codes, unique identifiers, or web links for publicly available datasets
- A description of any restrictions on data availability
- For clinical datasets or third party data, please ensure that the statement adheres to our policy

The raw records data are protected by a code of conduct, which is common to all Swiss na-tional data centres. An anonymised version of the records data, which can be used to repro-duce the occupancy-detection models, is available from Zenodo (https://doi.org/10.5281/zenodo.17256301).). The average occupancy estimates per species, two-year interval and biogeographic zone are available from Zenodo (https://doi.org/10.5281/zenodo.17255265). Data on traits as well as on drivers and other data necessary to reproduce the main analyses are available from Zenodo (https://doi.org/10.5281/zenodo.17256301).

Research involving human participants, their data, or biological material

Policy information about studies with human participants or human data. See also policy information about sex, gender (identity/presentation), and sexual orientation and race, ethnicity and racism.

Reporting on sex and gender	*Use the terms sex (biological attribute) and gender (shaped by social and cultural circumstances) carefully in order to avoid confusing both terms. Indicate if findings apply to only one sex or gender; describe whether sex and gender were considered in study design; whether sex and/or gender was determined based on self-reporting or assigned and methods used. Provide in the source data disaggregated sex and gender data, where this information has been collected, and if consent has been obtained for sharing of individual-level data; provide overall numbers in this Reporting Summary. Please state if this information has not been collected. Report sex- and gender-based analyses where performed, justify reasons for lack of sex- and gender-based analysis.*
Reporting on race, ethnicity, or other socially relevant groupings	*Please specify the socially constructed or socially relevant categorization variable(s) used in your manuscript and explain why they were used. Please note that such variables should not be used as proxies for other socially constructed/relevant variables (for example, race or ethnicity should not be used as a proxy for socioeconomic status). Provide clear definitions of the relevant terms used, how they were provided (by the participants/respondents, the researchers, or third parties), and the method(s) used to classify people into the different categories (e.g. self-report, census or administrative data, social media data, etc.) Please provide details about how you controlled for confounding variables in your analyses.*
Population characteristics	*Describe the covariate-relevant population characteristics of the human research participants (e.g. age, genotypic information, past and current diagnosis and treatment categories). If you filled out the behavioural & social sciences study design questions and have nothing to add here, write "See above."*
Recruitment	*Describe how participants were recruited. Outline any potential self-selection bias or other biases that may be present and how these are likely to impact results.*
Ethics oversight	*Identify the organization(s) that approved the study protocol.*

Note that full information on the approval of the study protocol must also be provided in the manuscript.

Field-specific reporting

Please select the one below that is the best fit for your research. If you are not sure, read the appropriate sections before making your selection.

☐ Life sciences ☐ Behavioural & social sciences ☒ Ecological, evolutionary & environmental sciences

For a reference copy of the document with all sections, see nature.com/documents/nr-reporting-summary-flat.pdf

Ecological, evolutionary & environmental sciences study design

All studies must disclose on these points even when the disclosure is negative.

Study description	In this study, we determined mean bi-annual occupancy of 811 insect species (595 saproxylic beetle species, 216 butterfly species [Papilionoidea, incl. Zygaenidae moths]) for the period 1931–2021 in six biogeographic zones of Switzerland. These occupancy estimates were based on records data that originate from the info fauna database (www.infofauna.ch). To calculate mean occupancies, we divided our study region into 5 km × 5 km squares and determined occurrence probability for each square, two-year interval and species using occupancy-detection models (1,500 squares for saproxylic beetles, 1,719 squares for butterflies). Mean bi-annual occupancy estimates per species and biogeographic zone were used to reconstruct species richness trends per zone for the years 1931–2021 (sum of mean occupancies). Furthermore, we divided the study period into consecutive eight-year intervals and reconstructed a set of environmental variables for these intervals based on census and climate data. We related species richness trends in the same intervals to the environmental variables to analyse the main drivers of richness changes. Finally, we aggregated species richness estimates for different trait-based groups to analyse how temporal trends of species depend on their traits.

Research sample	All species records data originated from the curated records database hosted by info fauna (www.infofauna.ch). The two insect groups (saproxylic beetles, butterflies) were selected based on data availability and to cover different functional groups with different ecological requirements. The geographic range of the data (Switzerland) was set by the database and the availability of data on environmental conditions.
Sampling strategy	All records data available for the study period were used (after data curation). The selection of species, for which occupancy-detection models were fitted, was based on data availability (predefined threshold of being recorded in at least 25% of the analysed two-year intervals).
Data collection	Species records included in the info fauna database have various origins, including different projects (e.g. research, Red List inventories) and observations from species experts and amateur naturalists. Parts of the data originate from digitized museum collections. The database is curated by species experts and only contains records of high credibility.<br>To quantify environmental conditions, we used census data (e.g. agricultural censuses, see Table S3 for sources) and reconstructed climate data provided by MeteoSwiss (www.meteoswiss.admin.ch).<br>Species traits were calculated from several datasets (refs 20, 36, 75, 76, 77, 78, 79) and gaps were filled based on expert knowledge.
Timing and spatial scale	Species records from the years 1931–2021 and from whole of Switzerland were used.
Data exclusions	The species records database is curated by species records and does not contain records of low credibility. We further exlcluded:<br>- Species records with too low spatial resolution (could not be attributed to squares)<br>- Data from 5 km × 5 km squares with only records from a single two-year interval (per dataset) (no reliable occurrence probability estimated possible)<br>- Duplicated records (same observer, square, visit and species) (add no information to the analyses)<br>- Saproxylic beetle records from observers that reported extraordinarily high numbers of species (57,444 records, cf. Fig. S8) (to prevent a bias in recent decades, in which observations of these observers clustered)
Reproducibility	All codes necessary to reproduce the analyses are available from a public repository (https://doi.org/10.5281/zenodo.17256301).
Randomization	This study used observational data from a curated database and did not rely on standardised sampling. Thus, randomization was not possible. In the occupancy-detection models, we account for confounding factors in the observation process (detection probability model).
Blinding	This is a study based on observational data from a curated database. Blinding was not relevant in this context.

Did the study involve field work? ☐ Yes ☒ No

Reporting for specific materials, systems and methods

We require information from authors about some types of materials, experimental systems and methods used in many studies. Here, indicate whether each material, system or method listed is relevant to your study. If you are not sure if a list item applies to your research, read the appropriate section before selecting a response.

Materials & experimental systems

n/a	Involved in the study	
☒	☐	Antibodies
☒	☐	Eukaryotic cell lines
☒	☐	Palaeontology and archaeology
☒	☐	Animals and other organisms
☒	☐	Clinical data
☒	☐	Dual use research of concern
☒	☐	Plants

Methods

n/a	Involved in the study	
☒	☐	ChIP-seq
☒	☐	Flow cytometry
☒	☐	MRI-based neuroimaging

Plants

Seed stocks	*Report on the source of all seed stocks or other plant material used. If applicable, state the seed stock centre and catalogue number. If plant specimens were collected from the field, describe the collection location, date and sampling procedures.*
Novel plant genotypes	*Describe the methods by which all novel plant genotypes were produced. This includes those generated by transgenic approaches, gene editing, chemical/radiation-based mutagenesis and hybridization. For transgenic lines, describe the transformation method, the number of independent lines analyzed and the generation upon which experiments were performed. For gene-edited lines, describe the editor used, the endogenous sequence targeted for editing, the targeting guide RNA sequence (if applicable) and how the editor was applied.*
Authentication	*Describe any authentication procedures for each seed stock used or novel genotype generated. Describe any experiments used to assess the effect of a mutation and, where applicable, how potential secondary effects (e.g. second site T-DNA insertions, mosiacism, off-target gene editing) were examined.*
