# Supplementary material for: Ninety-year trends reveal sharpest insect declines in the mid-twentieth century
Source: Nat Ecol Evol. 2026 Jun 2;10(6):1103–13. doi: 10.1038/s41559-026-03074-6 (PMC13253333; doi:10.1038/s41559-026-03074-6)
Supplement: Supplementary file 3 — Peer Review File [file 41559_2026_3074_MOESM3_ESM.pdf]

nature portfolio

Peer Review File

Ninety-year trends reveal sharpest insect declines mid-20th century

Corresponding Author: Dr Felix Neff

Version 0:

Decision Letter:

7th January 2026

*Please ensure you delete the link to your author homepage in this e-mail if you wish to forward it to your co-authors.

Dear Felix,

Your manuscript entitled "Ninety-year trends reveal sharpest insect declines mid-20th century" has now been assessed by two reviewers. As you will see from the reports below, both reviewers find the work of high interest and are in principle supportive of its publication in Nature Ecology & Evolution after addressing some concerns, including on the occupancy modelling and their validation. On the basis of this feedback, we would like to see a revision of the manuscript along with a response to the reviewers.

We are committed to providing a fair and constructive peer-review process. Do not hesitate to contact us if there are specific requests from the reviewers that you believe are technically impossible or unlikely to yield a meaningful outcome.

When revising your manuscript:

* Please provide the manuscript in Microsoft Word format and highlight all changes in the same file or a copy.

* Include a “Response to reviewers” document detailing, point-by-point, how you addressed each reviewer comment. If no action was taken to address a point, you must provide a compelling argument. This response will be sent back to the reviewers along with the revised manuscript.

* If you have not done so already please begin to revise your manuscript so that it conforms to our Article format instructions at http://www.nature.com/natecolevol/info/final-submission. Refer also to any guidelines provided in this letter.

* Include a revised version of any required reporting checklist. It will be available to referees (and, potentially, statisticians) to aid in their evaluation if the manuscript goes back for peer review. A revised checklist is essential for re-review of the paper.

* Extended Data Figures - please ensure that any supplementary figures and tables that are crucial to the manuscript’s conclusions are converted into Extended Data figures and tables to increase visibility of these data. Extended Data figures and tables are online-only (present in the online PDF and full-text HTML versions of the paper), peer-reviewed display items that provide essential background to the article but are not included in the main article due to space constraints. A maximum of ten Extended Data display items (figures and tables) is permitted.

We hope to receive your revised manuscript within two months. If you cannot send it within this time, please let us know. We will be happy to consider your revision so long as nothing similar has been accepted for publication at Nature Ecology & Evolution or published elsewhere.

Once ready, please use the link below to submit your revised manuscript and related files:

Link Redacted

Note: This URL links to your confidential home page and associated information about manuscripts you may have submitted, or that you are reviewing for us. If you wish to forward this email to co-authors, please delete the link to your homepage.

Nature Ecology & Evolution is committed to improving transparency in authorship. As part of our efforts in this direction, we are now requesting that all authors identified as ‘corresponding author’ on published papers create and link their Open Researcher and Contributor Identifier (ORCID) with their account on the Manuscript Tracking System (MTS), prior to acceptance. ORCID helps the scientific community achieve unambiguous attribution of all scholarly contributions. You can create and link your ORCID from the home page of the MTS by clicking on ‘Modify my Springer Nature account’. For more information please visit please visit www.springernature.com/orcid.

Please do not hesitate to contact me if you have any questions. We look forward to seeing the revised manuscript.

[redacted]

Reviewers' comments:

Reviewer #1 (Remarks to the Author):

This study is timely and vital. The discoveries made here are important, in my view, and the methods and data are strong. The shifting baseline problem is profoundly important and has led to serious problems in present understanding of how urgent the biodiversity crisis actually has become. So, this study contributes something that I find truly helpful to help improve understanding, and I think the findings here will contribute broadly to improved interpretation of long-term trends for biodiversity analysis.

I have a few very small things only to suggest.

The first of these suggestions is simply to avoid the use of technical terms whenever possible. I had to look up the meaning of stenotopic, which I have never seen before. Please find a common language term to replace such terminology, or define it at its first use. This is a really minor point.

Another really minor point: in map figure 1a, there is a region in the southern 1/3 or so of Switzerland that is coloured as a shade of purple. This colour does not seem to be present in the legend beside the map, and it doesn't seem to be explained in the figure legend below either. Please add to the figure legend to make this clearer. I am wondering if this might have happened because some of the colours in the figure are perhaps not completely opaque in the figure, so their apparent colour to the viewer is actually a mix of what is underneath also, and there is a kind of pink colour going on in the background. In any event, it should be easily sorted.

Occupancy models have a checkered history, sometimes leading to absurdly overfit outcomes that are basically meaningless. Validation against direct observations is critical, in my view. The authors present an approach to validation that is based on a previous study of theirs, and the evidence that the occupancy detection models work is that there is a pretty good distribution of high correlations in occupancy patterns between mostly independent species between one study and the other. This is indeed encouraging. It is also possible that these models converge for any number of other reasons that are unobserved. I recommend that greater effort around model validation be made. Are the primary observation data not able to demonstrate similar overall trends (albeit with potentially much larger variability because of uneven sampling) that the occupancy detection models do? This concerns me and since the occupancy models here are the basic building block of everything in the study, I'd like to ask for greater emphasis on model tests and validation.

Overall, this is an excellent study that I believe is important to a wide audience.

Jeremy Kerr
Biology, University of Ottawa
Canada

Reviewer #2 (Remarks to the Author):

Dear authors

Congratulations on this fantastic analysis of butterfly and saproxylic beetle diversity over 9 decades in Switzerland. It includes overall richness trends, and analyses of traits and drivers. This sort of analysis is exactly what the world needs. I have very few comments, and most relate to clarifications. Overall, you did a great job on this research, and I fully endorse its publication.

Roel van Klink

Minor comments
Fig 3b: here you elaborate on how to interpret the numbers in fig 3a, if I understand correctly. If I understand it right, the estimates in 3a are the departures from the group average, which is a fine approach. It is basically the same approach as in Hallmann et al 2017. E.g. for the butterflies, the positive effect of grassland area should be interpreted as a less negative effect. In any case, I don't have the feeling that 3b really makes anything clearer. I'd advise to make the legend and the axis label in 3a clearer to reflect that these are departures from the group average, or put differently: these factors change the overall slope.
Abstract: the last sentence is largely reiterating the title and abstract. Surely you can come up with a more inspired conclusion
Refs 1-3: a paper from 2025 can hardly be said to have sparked research into temporal insect declines. Technically, it was only the Hallmann paper that really set this off, although there were plenty of papers before that (e.g. by Forister, by Schuch 2012, or by Thomas 2004). Also the extreme decline rates found by Hallmann et al have very rarely been found elsewhere.

L56-58. I think you're underselling the contribution of the Habel et al papers here. Although there is enough to comment on these papers, that they only investigate butterflies, is hardly ground to be dismissive. After all, you only added one group to what they did. It would be good to be less dismissive.

Results: I'd recommend to always include the sign of the change when referring to a % change. So a decline of 30% would be -30%, just to be consistent with the credible intervals

L116: 'lowest' -> 'smallest'
180-190: this paragraph is a bit convoluted. The 'one hand' and 'other hand' are spaced far apart and hard to follow. 'among others' is also not a proper English sentence, and I don't quite know what you mean here.

212: this is a strange statement. I think you mean that well-dispersing species are less affected by land use than poor dispersers, rather than that they really benefit.

L618: add which levels of CI you calculated and display
L627 & 700: How does the first 8 y interval end in 1937, and the next one start in 1936??? They're clearly not consecutive
L723: I understand that these rights should be respected, but for reproducibility's sake, it would be important to have the exact same dataset potentially available for reanalysis. Please make arrangements to make this possible for a number of years.
L725: please add what the resolution of the GBIF dataset is. The metadata there are very sparse

Reviewer #2 (Remarks on code availability):

I have looked at the code, but did not attempt to run it. The data and analyses are too complex for this, and would probably take a long time to run. I'm reasonably sure that I could run it if I tried

The code package contains a read me file which is detailed enough to rerun the analysis. All r files and data files are included in the zip file on Zenodo.
The r scripts are sparsely annotated, although this seems to differ between files. The main analysis code is particularly sparsely annotated, and it is generally unclear what each piece of code / or function does.

In general, it looks like it could be used to rerun the analysis, and is thus a useful resource

Version 1:

Decision Letter:

19th March 2026

Dear Felix,

Your revised manuscript "Ninety-year trends reveal sharpest insect declines mid-20th century" (NATECOLEVOL-25114041A) has now been assessed by the two reviewers. On the basis of their feedback (see the reports below), we will be happy in principle to publish your work in Nature Ecology & Evolution, pending minor revisions to satisfy the reviewers' final requests and to comply with our editorial and formatting guidelines.

We are now performing detailed checks on your paper and will send you a checklist detailing our editorial and formatting requirements in about a week. Please do not upload the final materials and make any revisions until you receive this additional information from us.

If you have any questions, please feel free to contact me.

[redacted]

****

Reviewer #1 (Remarks to the Author):

I greatly appreciate the work you have done to revise an already-strong manuscript. I especially appreciate the extra effort you have undertaken around validating models, which I think will improve the confidence of readers who are aware that some occupancy models have failed to detect even very large effects of environmental change (because of overfitting). I have confidence in the modelling work performed here.

Otherwise, I believe this work will engage a broad audience and add something uniquely valuable to scientific understanding.

Jeremy Kerr, Biology
University of Ottawa

Reviewer #1 (Remarks on code availability):

I have not reviewed the code, as the other author has done so already, and it is most unlikely that I would detect any challenges there (and the code would take too long to run in any event).

Reviewer #2 (Remarks to the Author):

Dear authors

I am happy with the changes you made, and the manuscript is now almost ready for publication. I really like the added sensitivity analyses, and have just a few small remarks:
- The added text referring to the figures of the new sensitivity analyses (L447 - 456) is now only included in the methods text. I would recommend adding 1 sentence to the main text, so that the average reader is also aware of these analyses. Something along the lines of "We compared these results to 1) red list trends and 2) the Swiss butterfly monitoring scheme, and found strong congruence (Fig S9, S10)".
- In the added text referring to the figures of the new sensitivity analyses (L447 - 456), the wording 'one one hand', 'on the other hand' is somewhat misplaced, as there is no disagreement between the comparisons. I'd recommend simply listing 'first' and ' second'

- L182 replace 'that', with ', which'. I think there may be more instances where 'that' and 'which' may have been swapped.

Roel van Klink

Open Access This Peer Review File is licensed under a Creative Commons Attribution 4.0 International License, which permits use, sharing, adaptation, distribution and reproduction in any medium or format, as long as you give appropriate credit to the original author(s) and the source, provide a link to the Creative Commons license, and indicate if changes were

made.
In cases where reviewers are anonymous, credit should be given to 'Anonymous Referee' and the source.
The images or other third party material in this Peer Review File are included in the article’s Creative Commons license, unless indicated otherwise in a credit line to the material. If material is not included in the article’s Creative Commons license and your intended use is not permitted by statutory regulation or exceeds the permitted use, you will need to obtain permission directly from the copyright holder.
To view a copy of this license, visit https://creativecommons.org/licenses/by/4.0/

Reviewer #1 (Remarks to the Author):

This study is timely and vital. The discoveries made here are important, in my view, and the methods and data are strong. The shifting baseline problem is profoundly important and has led to serious problems in present understanding of how urgent the biodiversity crisis actually has become. So, this study contributes something that I find truly helpful to help improve understanding, and I think the findings here will contribute broadly to improved interpretation of long-term trends for biodiversity analysis.

Thank you very much for the positive feedback on our study.

I have a few very small things only to suggest.

The first of these suggestions is simply to avoid the use of technical terms whenever possible. I had to look up the meaning of stenotopic, which I have never seen before. Please find a common language term to replace such terminology, or define it at its first use. This is a really minor point.

We changed the terms and now call it 'high', 'moderate' and 'low' (habitat) specialisation throughout. We still mention the original terminology in the methods for the readers more familiar with these terms (L473-475; L484). Adjustments to both text and figures were made.

Another really minor point: in map figure 1a, there is a region in the southern 1/3 or so of Switzerland that is coloured as a shade of purple. This colour does not seem to be present in the legend beside the map, and it doesn't seem to be explained in the figure legend below either. Please add to the figure legend to make this clearer. I am wondering if this might have happened because some of the colours in the figure are perhaps not completely opaque in the figure, so their apparent colour to the viewer is actually a mix of what is underneath also, and there is a kind of pink colour going on in the background. In any event, it should be easily sorted.

Thank you for pointing this out. We had a greyscale representing the elevation in the background of the map, which we did not clearly explain in the legend. We understand that this was rather confusing. To make the figure clearer, we removed the elevation information (it is still partly accessible through the map inlet of Europe).

Occupancy models have a checkered history, sometimes leading to absurdly overfit outcomes that are basically meaningless. Validation against direct observations is critical, in my view. The authors present an approach to validation that is based on a previous study of theirs, and the evidence that the occupancy detection models work is that there is a pretty good distribution of high correlations in occupancy patterns between mostly independent species between one study and the other. This is indeed encouraging. It is also possible that these models converge for any number of other reasons that are unobserved. I recommend that greater effort around model validation be made. Are the primary observation data not able to demonstrate similar overall trends (albeit with potentially much larger variability because of uneven sampling) that the occupancy detection models do? This concerns me and since the occupancy models here are the basic building block of everything in the study, I'd like to ask for greater emphasis on model tests and validation.

We are aware that occupancy-detection models might result in overly confident results. To cover periods of such length, they are however of great value, as they are one of the few options to analyse the rare and opportunistic datasets that exist over so many decades.

When developing the modeling approach, we deliberately chose our model specifications in alignment with previous studies that successfully used and as far as possible validated occupancy-detection models (Bowler et al. 2021 Diversity and Distributions; Neff et al. 2022 Nature Communications; Outhwaite et al. 2019 Scientific Data), in some cases also with rather small datasets per species. Still, we acknowledge that we should have put more emphasis on model validation in the present study. Analyses of primary observation data, as suggested, are not reasonably possible, given the large changes in observation efforts over the study period (cf. Fig. S7). To still validate our models, we now directly compare modelled occupancies per species to two additional datasets, which contain information on temporal changes in some of the study species and serve as additional validation analyses:

First, we compared species-level 90-year trends in average occupancy to the Red List status of these species assigned by the Red Lists of Switzerland. Red Lists exist for butterflies (214 of the studies species) and some saproxylic beetle families (198 of the studied species). If occupancy-detection model outcomes are reliable, we would expect a close alignment of long-term trends and Red List status, which is what we found for both butterflies and beetles (Fig. S9).

Second, a standardized, Switzerland-wide monitoring scheme exists for butterflies since 2003. We estimated species-level distribution trends based on this Biodiversity Monitoring (BDM) scheme for the years 2003–2021 and compared the trends to species-level distribution trends based on our occupancy-detection models for the years 2002–2021 (includes 2002 because of the aggregation at two-year intervals). Since the BDM data were also included in the records data that were used for the occupancy-detection models, we rerun the occupancy-detection models on a subset of the data not including these BDM data to prevent circularity in the analyses. We restricted these additional analyses to the years since 1990 to reduce computing time, while avoiding a bias due to different starting conditions. For the 129 butterfly species for which we could estimate trends based on both datasets, we found a very clear correlation between the two datasets (Fig. S10).

The two validation analyses did further strengthen the trust in our models and we are thus very confident that they reflect true large-scale and long-term distributional changes of the studied insects in Switzerland.

Besides the two additional supplementary figures, we added additional text covering these validation analyses in the methods. It reads: “While we based our modelling approach on approaches that were shown to be robust[24,69,72], we further validated the estimated occupancies with additional available data. On the one hand, we compared linear 90-year trends per species to their Red List status for species that were assessed in the available Swiss Red Lists[73,74] (198 saproxylic beetle and 214 butterfly species). We found Red List status and species trends to be clearly related (Fig. S9). On the other hand, we compared butterfly species trends based on occupancy-detection models to species trends based on a standardised, Switzerland-wide sampling scheme that is in place since 2003. For the overlapping 19 years, we found very high alignment between the trends based on the two different data sources (Fig. S10).» (L447–456)

Overall, this is an excellent study that I believe is important to a wide audience.

Jeremy Kerr
Biology, University of Ottawa
Canada

Thank you very much for your time to evaluate our study.

Reviewer #2 (Remarks to the Author):

Dear authors

Congratulations on this fantastic analysis of butterfly and saproxylic beetle diversity over 9 decades in Switzerland. It includes overall richness trends, and analyses of traits and drivers. This sort of analysis is exactly what the world needs. I have very few comments, and most relate to clarifications. Overall, you did a great job on this research, and I fully endorse its publication.

Roel van Klink

Thank you very much for the positive feedback on our study.

Minor comments

Fig 3b: here you elaborate on how to interpret the numbers in fig 3a, if I understand correctly. If I understand it right, the estimates in 3a are the departures from the group average, which is a fine approach. It is basically the same approach as in Hallmann et al 2017. E.g. for the butterflies, the positive effect of grassland area should be interpreted as a less negative effect. In any case, I don't have the feeling that 3b really makes anything clearer. I'd advise to make the legend and the axis label in 3a clearer to reflect that these are departures from the group average, or put differently: these factors change the overall slope.

Thank you for this point. Our intention behind 3b was to make the biological meaning of the model estimates clearer, but we agree that it somewhat complicates things. We removed Figure 3b and adjusted the figure legend of Figure 3 to include the information on the biological meaning behind the model estimates.

Abstract: the last sentence is largely reiterating the title and abstract. Surely you can come up with a more inspired conclusion

We agree and changed the last sentence of the abstract. It now reads: “To maintain and promote insect diversity, reducing adverse land use will be key, while shifted baselines and climate-change effects need to be acknowledged when setting restoration goals and priorities.” (L44–46)

Refs 1-3: a paper from 2025 can hardly be said to have sparked research into temporal insect declines. Technically, it was only the Hallmann paper that really set this off, although there were plenty of papers before that (e.g. by Forister, by Schuch 2012, or by Thomas 2004). Also the extreme decline rates found by Hallmann et al have very rarely been found elsewhere.

Thank you for pointing this out. We slightly adapted the text and changed the position of the later references. It now reads: “Alarming findings on a massive insect decline[1] have sparked research into temporal trends in insect diversity[2,3].” (L48–49)

L56-58. I think you're underselling the contribution of the Habel et al papers here. Although there is enough to comment on these papers, that they only investigate butterflies, is hardly ground to be dismissive. After all, you only added one group to what they did. It would be good to be less dismissive.

We certainly did not intend to be dismissive; rather, we wanted to highlight what our study can contribute to these previous publications, which are certainly very interesting and

important. While we largely kept the part that lists the limitations of previous studies, we changed the text following that part. As such, we hope that our message will no longer be perceived as dismissive. The part now reads: "The few studies that cover more than a few decades are often limited to single time points (e.g., data from the 20th compared to the 21st century[7]), to several discrete time windows over the 20th century[8], or to a small number of study sites or single taxonomic groups when analysing continuous data[9–12]. Most of these studies found support for long-term declines in insect diversity. A continuous long-term time series of insect diversity, covering a large spatial scale and different insect groups, is still lacking, but will help to better understand community changes over more than 40 years – including the time of large-scale agricultural intensification in the second half of the 20th century[13]." (L52–60)

Results: I'd recommend to always include the sign of the change when referring to a % change. So a decline of 30% would be -30%, just to be consistent with the credible intervals

Thank you for this suggestion, which we have implemented throughout.

L116: 'lowest' -> 'smallest'

Thank you, we changed the wording.

180-190: this paragraph is a bit convoluted. The 'one hand' and 'other hand' are spaced far apart and hard to follow. 'among others' is also not a proper English sentence, and I don't quite know what you mean here.

We apologize for not being very clear here. We adjusted the paragraph, it now reads: "[…] In forests, near-to-nature management became widely adopted, which for example resulted in an increase in deadwood that should have promoted many saproxylic beetles species[29]. Indeed, the change in forest management undoubtedly contributed to the recovery of saproxylic beetle richness in the second half of the study period (Fig. 2). In open lands, the introduction of agri-environmental measures in the 1990s and the subsequent increase in the amount and connectivity of suitable habitats in agricultural areas probably contributed to stabilising butterfly richness trends[34] (Fig. 2). […]" (L181–187)

212: this is a strange statement. I think you mean that well-dispersing species are less affected by land use than poor dispersers, rather than that they really benefit.

You are certainly right, we changed the wording. It now reads: "[…] global change drivers such as land-use intensification are known to harm species with lower dispersal ability disproportionately[40]." (L209–210)

L618: add which levels of CI you calculated and display

We specified the text to "95%-CIs".

L627 & 700: How does the first 8 y interval end in 1937, and the next one start in 1936??? They're clearly not consecutive

That is because we aggregated species records data into two-year intervals. Thus, the first eight-year interval based on two-year intervals ends in 1937 (two-year interval 1936/37). The consecutive eight-year interval starts with 1936/1937 to have fully consecutive intervals. For yearly data, such as many environmental data, this means that consecutive eight-year intervals overlap by two years.

We now make this clearer in the text. It reads:

"[…] we divided the study period into 15 consecutive eight-year intervals (1930–1937, 1936–1943, …, 2014–2021). The overlap of consecutive intervals is a consequence of the two-year intervals at which richness was estimated. For example, the first eight-year interval of richness estimates ends with 1936/37 and the second starts with 1936/37 to avoid gaps between consecutive intervals." (L513–517)

and

" [..] the 15 eight-year intervals (1930–1937, 1936–1943, …, 2014–2021; overlap because of richness estimates for two-year intervals) (n = 90)." (L588–590)

L723: I understand that these rights should be respected, but for reproducibility's sake, it would be important to have the exact same dataset potentially available for reanalysis. Please make arrangements to make this possible for a number of years.

We now make an anonymized version of the raw data available in the corresponding Zenodo repository. It allows to fully reproduce the occupancy-detection models. As these analyses analyses require a lot of resources (time, computational power), the aggregated occupancy means resulting from the occupancy-detection models remain available. These data may be of particular interest for future studies.

We updated the data availability statement accordingly.

L725: please add what the resolution of the GBIF dataset is. The metadata there are very sparse

This point is no longer relevant, as we now provide raw data directly (see previous point).

Reviewer #2 (Remarks on code availability):

I have looked at the code, but did not attempt to run it. The data and analyses are too complex for this, and would probably take a long time to run. I'm reasonably sure that I could run it if I tried

Thank you for checking the codes as well. Yes, analyses take long to run.

The code package contains a read me file which is detailed enough to rerun the analysis. All r files and data files are included in the zip file on Zenodo.
The r scripts are sparsely annotated, although this seems to differ between files. The main analysis code is particularly sparsely annotated, and it is generally unclear what each piece of code / or function does.

We now annotate the code more thoroughly. An updated version of the repository is available from Zenodo.

In general, it looks like it could be used to rerun the analysis, and is thus a useful resource

Thank you, we put great effort into making codes reproducible.

Response to reviewers

Reviewer #1 (Remarks to the Author):

I greatly appreciate the work you have done to revise an already-strong manuscript. I especially appreciate the extra effort you have undertaken around validating models, which I think will improve the confidence of readers who are aware that some occupancy models have failed to detect even very large effects of environmental change (because of overfitting). I have confidence in the modelling work performed here.

Otherwise, I believe this work will engage a broad audience and add something uniquely valuable to scientific understanding.

Jeremy Kerr, Biology
University of Ottawa

Thank you for taking the time to evaluate our study. We were pleased by the positive feedback on our work and the additional validation efforts.

Reviewer #1 (Remarks on code availability):

I have not reviewed the code, as the other author has done so already, and it is most unlikely that I would detect any challenges there (and the code would take too long to run in any event).

Reviewer #2 (Remarks to the Author):

Dear authors

I am happy with the changes you made, and the manuscript is now almost ready for publication. I really like the added sensitivity analyses, and have just a few small remarks:
- The added text referring to the figures of the new sensitivity analyses (L447 - 456) is now only included in the methods text. I would recommend adding 1 sentence to the main text, so that the average reader is also aware of these analyses. Something along the lines of "We compared these results to 1) red list trends and 2) the Swiss butterfly monitoring scheme, and found strong congruence (Fig S9, S10)".

Thank your for this suggestion. We added an extra sentence, it reads: “We compared the estimated temporal changes of species’ mean occupancies to red list status and a temporally restricted standardised butterfly monitoring scheme and found strong con-gruence (Figs S1–S2).” (L80–82). Also, we re-indexed all supplementary figures in the main text and the supplementary material.

- In the added text referring to the figures of the new sensitivity analyses (L447 - 456), the wording 'one one hand', 'on the other hand' is somewhat misplaced, as there is no disagreement between the comparisons. I'd recommend simply listing 'first' and ' second'

Thank you for pointing this out, we changed the text to “first” and “second”, as suggested. (L411–414).

- L182 replace 'that', with ', which'. I think there may be more instances where 'that' and 'which' may have been swapped.

We rechecked the whole text and adjusted it in several instances (e.g. L185-187, L210, L256–257, L265–266, L286, L315).

Roel van Klink

Thank your for taking the time to evaluate our study.
